# The role of rapid diagnostics in managing Ebola epidemics

**DOI:** 10.1038/nature16041

**Published:** 2015-12-03

**Authors:** Pierre Nouvellet, Tini Garske, Harriet L. Mills, Gemma Nedjati-Gilani, Wes Hinsley, Isobel M. Blake, Maria D. Van Kerkhove, Anne Cori, Ilaria Dorigatti, Thibaut Jombart, Steven Riley, Christophe Fraser, Christl A. Donnelly, Neil M. Ferguson

**Affiliations:** 1 MRC Centre for Outbreak Analysis and Modelling, Department of Infectious Disease Epidemiology, Faculty of Medicine, Imperial College London, Norfolk Place, London W2 1PG, UK; 2 Center for Global Health, Institut Pasteur, 25 rue du Docteur Roux, 75724 Paris Cedex 15, France

## Abstract

Ebola emerged in West Africa around December 2013 and swept through Guinea, Sierra Leone and Liberia, giving rise to 27,748 confirmed, probable and suspected cases reported by 29 July 2015. Case diagnoses during the epidemic have relied on polymerase chain reaction-based tests. Owing to limited laboratory capacity and local transport infrastructure, the delays from sample collection to test results being available have often been 2 days or more. Point-of-care rapid diagnostic tests offer the potential to substantially reduce these delays. We review Ebola rapid diagnostic tests approved by the World Health Organization and those currently in development. Such rapid diagnostic tests could allow early triaging of patients, thereby reducing the potential for nosocomial transmission. In addition, despite the lower test accuracy, rapid diagnostic test-based diagnosis may be beneficial in some contexts because of the reduced time spent by uninfected individuals in health-care settings where they may be at increased risk of infection; this also frees up hospital beds. We use mathematical modelling to explore the potential benefits of diagnostic testing strategies involving rapid diagnostic tests alone and in combination with polymerase chain reaction testing. Our analysis indicates that the use of rapid diagnostic tests with sensitivity and specificity comparable with those currently under development always enhances control, whether evaluated at a health-care-unit or population level. If such tests had been available throughout the recent epidemic, we estimate, for Sierra Leone, that their use in combination with confirmatory polymerase chain-reaction testing might have reduced the scale of the epidemic by over a third.

The unprecedented scale of the 2014–15 West African Ebola epidemic has posed major challenges for delivering rapid diagnosis — an essential component of controlling Ebola epidemics, given the non-specific nature of early clinical symptoms. Following World Health Organization (WHO) guidelines, testing has relied on reverse transcription polymerase chain reaction (RT–PCR) based methods, which detect viral RNA in serum or plasma^1^. However, these tests are slow and costly, taking 2–6 hours to process^2^ at around US$100 per test. Testing requires high levels of biosafety, and both samples and reagents must be kept cold or frozen^1,3–6^. Sustaining a cold-chain has been particularly challenging in the affected countries because of the limited infrastructure, frequent power cuts and hot climate. In addition, collecting venous blood for PCR testing requires specific medical training and poses significant risks for health-care workers^7^.

Furthermore, although the laboratory processing time for PCR testing can be under 6 hours, the time between sample collection and receiving the result has often been much longer during the epidemic, owing to limited laboratory capacity and logistical infrastructure^8^. This has been a crucial issue, because delays in testing lead to longer hospital stays for patients, increasing both bed demand and the likelihood of nosocomial transmission (a major issue in Ebola outbreaks^9^). At the peak of the epidemic in Sierra Leone (between October and November 2014) there were reports of delays in test results of up to 1 week^10^. Only in January 2015, following an effort to reduce delays in laboratory access^11^, did the WHO report substantially reduced average waiting times for test results of between 1.3 and 2.3 days, depending on the country^12^.

Recognizing the extraordinary circumstances of the epidemic, in November 2014 WHO issued a call for “rapid, sensitive, safe and simple Ebola diagnostic tests”^2^. An ideal rapid diagnostic test (RDT) would require minimal laboratory facilities and staff training, no cold chain and could be performed with a capillary blood sample collected through a finger prick. Such an RDT could be used at the point of care to allow faster triaging of people suspected of having Ebola, returning a test result in minutes rather than days.

As a result of the WHO initiative, four tests have already been approved, including the benchmark PCR test, Realstar^13^, and an RDT, ReEBOV^14–16^ (Table 1). In addition, the US Food and Drug Administration has authorized ten tests for emergency use^17^ and multiple alternative RDTs are in development (Supplementary Table 1).

Although recognizing that rapid and accurate diagnostics are crucial to successful containment of an Ebola outbreak, determining the best strategy and the impact of RDTs is not straightforward (Fig. 1). Poor diagnostic specificity risks patients without Ebola infection being admitted to Ebola treatment units (ETUs) — where they potentially acquire infection, and use a bed that could otherwise be used by a patient who does have Ebola. Conversely, poor diagnostic sensitivity can lead to infected individuals being discharged back to the community or sent to non-Ebola-specific wards, with the consequent risk of onward transmission. In this Review, we use mathematical models of Ebola transmission to explore how RDTs might best be used in future outbreaks, balancing the rapid availability of RDT results against the lower sensitivity and specificity of such tests. This trade-off makes optimization of testing strategies complex and context-dependent, from both a technical and ethical perspective.

## METHODS

We examine RDTs using three different metrics, characterizing their impact on individual patient outcomes (represented by the expected case fatality ratio (CFR) for a person suspected of having Ebola who is seeking care), the effectiveness with which health-care units reduce transmission (represented by the reproduction number for a person with a true Ebola infection seeking care), and the overall scale of an epidemic (represented by the total number of cases). We evaluate the first two metrics using a model that focuses on the impact of different testing strategies implemented in the context of a single health-care unit, and the third using a model of the transmission dynamics of Ebola in Sierra Leone as a whole.

### Potential impact of RDTs in a health-care unit

**Figure 1:**
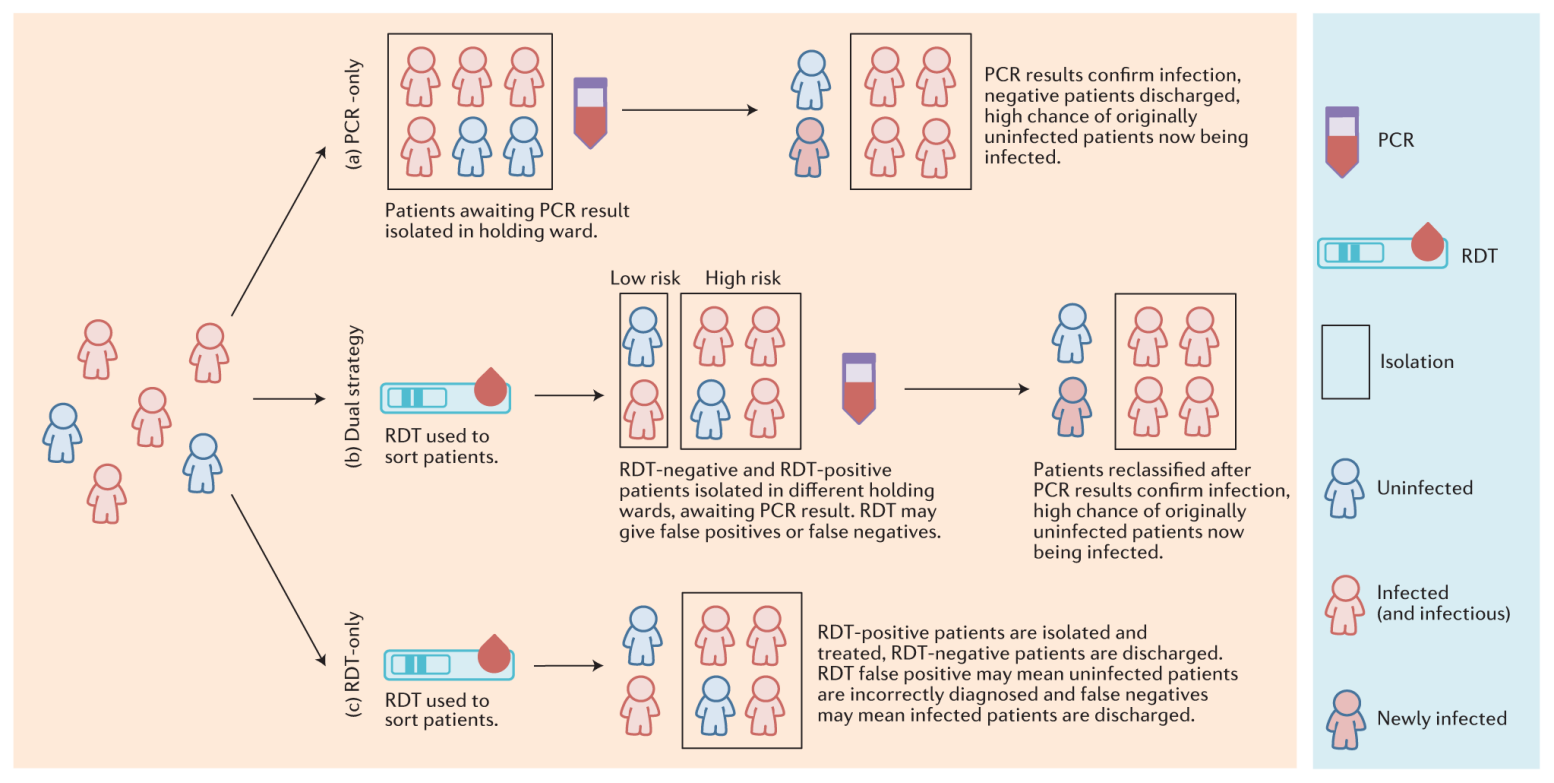
Three Ebola health-care-unit diagnostic testing and patient triage strategies. Patients seeking care are first admitted to a holding area where they wait to be either admitted to a confirmed ward or discharged back to the community. We examine three diagnostic testing strategies. **a**, Polymerase chain reaction (PCR)-only: patients await their test results in a single holding area. When PCR test results become available, individuals are either sent to a confirmed ward or discharged back to the community. **b**, Dual strategy (rapid diagnostic test (RDT) and PCR): based on initial RDT results, patients seeking care are kept in separate high- or low-risk wards. When PCR test results become available, individuals are either sent to a confirmed ward or discharged back to the community. **c**, RDT-only: the RDT result alone determines who is sent to a confirmed ward or discharged back to the community. Patients are either infected (red), uninfected (blue) or exposed within the holding area (infected, but not yet infectious, blue outline and red centre). The RDT is assumed to have a lower sensitivity and may give incorrect classifications (false positives and false negatives), which can result in nosocomial further transmission in the community if true Ebola cases are erroneously discharged.

Figure 1 (and Supplementary Information 2) illustrates the dynamics of patients within a health-care unit represented in the health-care-unit-level model that we developed to examine three diagnostic testing strategies: PCR-only, dual testing and RDT-only. Patients are tested within the holding area or areas to determine who should be sent to a confirmed ward and who should be discharged back to the community (with the daily number of patients seeking care and *p* the proportion who are infected with Ebola). During the time spent in holding, patients without Ebola have a daily risk of Ebola infection of *β*y, where *β* is the transmission rate and *y* is the prevalence of Ebola infection among other patients in that holding area. A similar risk affects patients mis-diagnosed with Ebola in the confirmed ward. At baseline we assume *β* = 0.15 per day.

For analytical tractability, we model a single generation of infection: people with suspected Ebola who enter the health-care unit uninfected, but then become infected and are assumed to be discharged back to the community or sent to the confirmed ward before they become infectious. When bed capacity (the total number of beds in the health-care unit) has been exceeded by demand, we assume that patients seeking care are turned away and return to the community.

For each testing strategy we use this health-care-unit-level model to evaluate how many infected patients seeking care are sent to the confirmed ward and how many are discharged back to the community (owing to bed shortage or false-negative diagnosis when using the RDT-only strategy). In addition, and again for each testing strategy, we determine how many patients without Ebola who seek care were infected during their stay in the hospital (in the holding and/or confirmed wards).

To evaluate the impact of a testing strategy from a patient perspective, we compare the CFR among people with suspected Ebola seeking care with the CFR among those same patients had they not sought care (community CFR). Assuming that care reduces the CFR, hospitalization improves the outcome for infected patients. However, patients without Ebola within a health-care unit are at risk of nosocomial transmission; hence hospitalization is not necessarily optimal for a patient with unknown Ebola infection status. We use our model to explore these trade-offs and to determine the epidemiological contexts in which the different testing strategies are optimal.

We assume a CFR of 60% among patients infected with Ebola throughout^18^. Patients without Ebola seeking care in health-care units are likely to present with similar symptoms to those with the infection, including high fever, vomiting, diarrhoea and haemorrhaging. These are symptoms that are typically observed among patients with severe Lassa fever, a disease highly prevalent in West Africa. Therefore, we assume a 20% CFR among patients without Ebola, which is comparable with the reported CFR for severe Lassa fever cases admitted to hospital^19^. Furthermore, we assume that all patients admitted to the confirmed ward (including those without Ebola) benefit from hospital care, with *r* being the relative CFR of a hospitalized patient with Ebola (*r* = 1 representing no benefit of hospitalization). All patients sent to the confirmed ward are assumed to stay in the health-care unit for an average of 7 days.

The second important role of health-care units in Ebola outbreaks is to reduce transmission by isolating cases. To evaluate the impact of testing strategies on transmission at the level of a single health-care unit, we use our health-care-unit-level model to calculate the reproduction number of Ebola-infected patients seeking care, *R*_HU_ (the average number of secondary infections generated by these patients). This reproduction number reflects the potential impact of health-care units in reducing transmission and depends on the testing strategy used. We assess the epidemiological contexts for which different testing strategies are optimal.

In examining both the patient and transmission perspectives, we explore likely scenarios at four different stages of the epidemic: in the early growth phase, at the peak, after the peak, and in the tail of the outbreak (reflecting the situation around June 2014, November 2014, January 2015 and May 2015, respectively). Over the course of the epidemic, we assume that the incidence of true cases increases, peaks and then declines; the number of patients without Ebola seeking care initially increases (for example, due to rising awareness as case numbers increase), and remains high as the epidemic wanes (meaning the prevalence of Ebola infection among suspected cases seeking care, *p*, wanes over time); the reproduction number among those not seeking care (community reproduction number) decreases, reflecting improved community control of transmission (for example, safer funeral practices); bed capacity increases and then plateaus once the epidemic starts to decline (see Table 2 for parameter values).

We implemented the health-care-unit-level model as an Excel spreadsheet and Java program (see Supplementary Information). This freely available software allows the reader to explore the impact of different model assumptions and parameter values on model outcomes.

### Potential impact of RDTs at the population level

To evaluate the potential impact of RDT use on the overall trajectory of an Ebola epidemic at the population level (compared with the impact at an individual- or health-care-unit level) we developed a susceptible–exposed–infectious–recovered (SEIR)-type transmission model, which was extended to include a highly infectious ‘near death’ stage reflecting increased transmission around the time of death and at funerals (see Supplementary Information 3.1.1). The model incorporates observed delays between key epidemiological events and is parameterized to reproduce the basic reproduction number, *R*_0_, observed during the Sierra Leone epidemic^18,20^ (model details are given in Supplementary Information 3).

We investigate the same diagnostic strategies as in the health-care-unit-level model (Fig. 1), but allow for full disease progression within each ward and in the community, with the flows between community and hospital wards being determined by the testing delays and test characteristics. As in the health-care-unit-level model, bed capacity and utilization are tracked such that once all beds are occupied, patients seeking care cannot be admitted and therefore remain in the community.

The model incorporates observed changes in the bed capacity available during the epidemic, making use of data on the number of ETU beds opened over time from the start of the outbreak to the end of May 2015. We include a constant number of 60 beds throughout to represent informal holding units and other health-care facilities that were available before the start of the epidemic and were used for Ebola patients before the scale-up of ETU beds. We vary the mean onset to hospitalization delay each month to match reported performance indicators^21^. At the start of the epidemic, the relative infectiousness of a non-hospitalized person with Ebola during the late stage of infection close to, and shortly after, death is assumed to be 16-fold higher than infectiousness earlier in disease progression, but this factor is allowed to decrease over time. Higher late-stage infectiousness allows the model to represent the enhanced risk of transmission to those caring for dying patients at home, preparing the corpse of a family member or undertaking funeral rites. We chose this value to yield a similar overall probability of onward transmission from fatalities as that of survivors despite the shorter infectious period of fatal cases. Thus, around half of community transmission from a non-hospitalized fatal case occurs around the time of death. We assume safe handling of corpses and burials for all deaths in confirmed wards of health-care units.

Assuming no use of RDTs, transmission parameters are calibrated to match the time series of observed incidence (confirmed and probable cases) and bed occupancy in Sierra Leone. The parameters calibrated include *R*_0_, the initial number of infectious cases present at the start of the simulation, the daily rate of hospitalizations of people without Ebola, the probability of care seeking for infected patients and the decrease in death-associated excess transmissibility in the community. These last two parameters were allowed to change at three time points: 1 October 2014, 1 November 2014 and 1 January 2015. The rate of people without Ebola seeking care is assumed to be proportional to the cumulative number of Ebola deaths up to that point.

For the baseline scenario in both health-care-unit-level and population-level models, we assume 100% sensitivity and specificity of PCR testing, and 92% sensitivity and 85% specificity for the RDT, matching the published performance of the ReEBOV test^15^. The average delays in obtaining RDT and PCR results are assumed to be 1 hour and 2 days, respectively.

We also examine the impact of reducing the delay in obtaining PCR test results from 2 days to 1 day, the minimum delay realistically achievable when laboratory facilities are located close to health-care units. Although current RDTs have limited sensitivity and specificity, the next generation of diagnostic tests may have substantially improved characteristics. We therefore also investigate the impact of using near-perfect RDTs, assuming sensitivity and specificity of 99% each.

Given the challenges faced during the epidemic such as sample collection, storage, transport and laboratory processing, it is unlikely that the high nominal sensitivity and specificity of PCR testing was achieved. Moreover, considerable variation in the sensitivity and specificity of ReEBOV RDT has been reported in the literature^14–16^. As a sensitivity analysis, we repeat the simulations using arguably more-realistic assumptions, regarding test sensitivity and specificity. We assume PCR test sensitivity and specificity of 85% and 95%, respectively, and RDT sensitivity and specificity of 82% and 80%, respectively, and recalibrate the remaining model parameters. The larger relative decreases in sensitivity and specificity of the PCR test compared with the RDT reflect the greater logistical constraints associated with the former.

## RESULTS

After calibrating the population-level transmission model, we estimate that 37% of people with Ebola sought health care at the start of the epidemic, increasing to 41%, 62% and 73% at the beginning of October 2014, November 2014 and January 2015, respectively. The relative risk of transmission associated with death is high up to the peak of the epidemic (16 until 30 September, and 15 until 31 October), and low after the epidemic peak (1.2 between 1 November and 31 December, and 1.1 from January onwards). The rate of patients without Ebola seeking care equates to around 120 patients per day at the end of the epidemic. The calibrated value of *R*_0_ is 1.7 (see Supplementary Table 11 and Supplementary Information 3.4 for sensitivity analyses with regards to the calibrated model parameters).

### Potential impact of RDTs in a health-care unit

**Figure 2:**
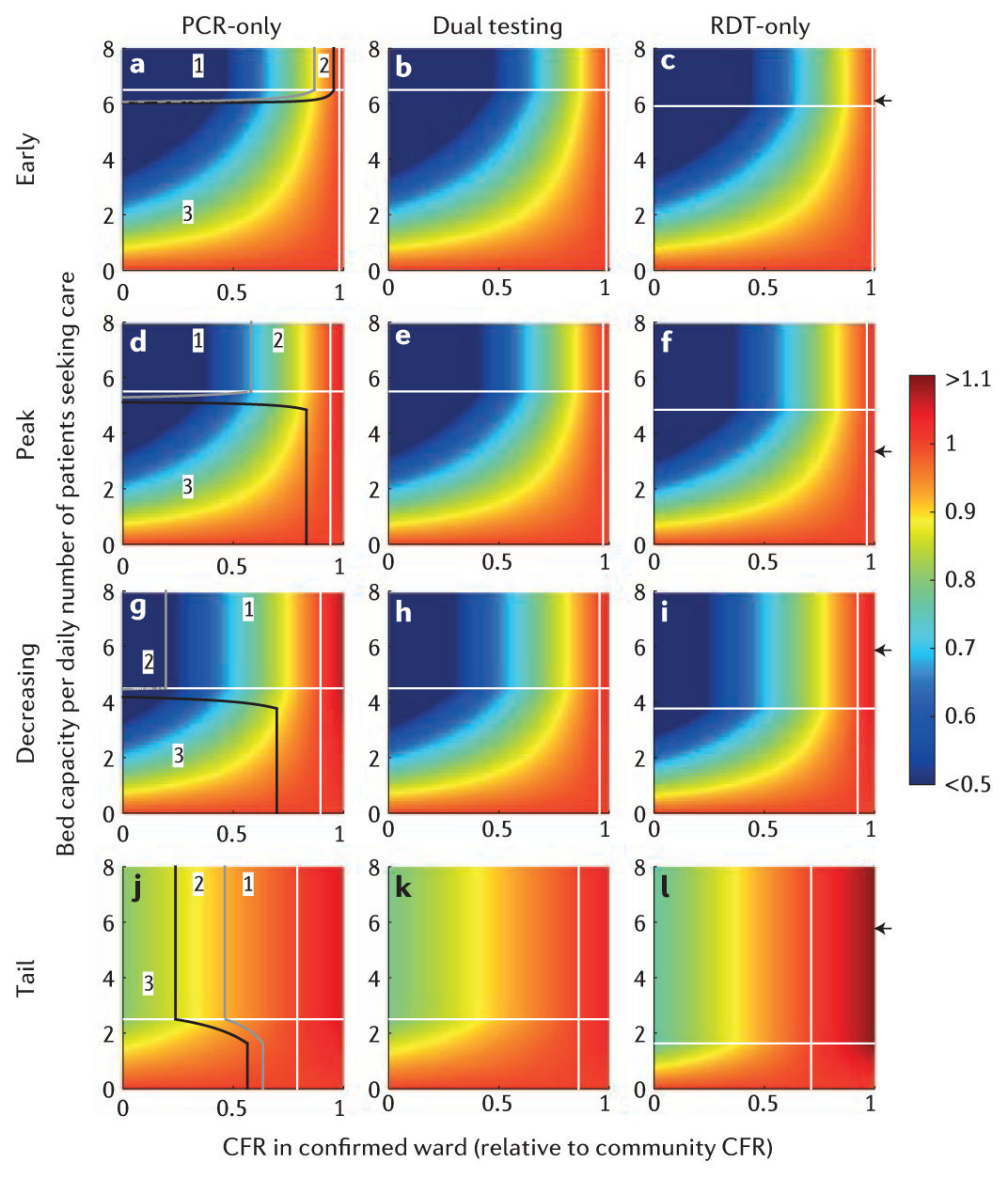
Case fatality ratio (CFR) of patients seeking care divided by the CFR if those same Ebola and non-Ebola cases had remained in the community. Each row represents a particular stage in the epidemic: from left to right: early (**a,b, c**), during the peak (**d,e,f**), shortly after the peak (**g,h,i**) and once the epidemic is tailing off(**j,k,l**). Each column reflects a testing strategy, namely polymerase chain reaction (PCR)-only (**a,d,g,j**), dual strategy (**b,e,h,k**) and rapid diagnostic test (RDT)-only (**c,f,i,l**). White horizontal lines show the threshold bed capacity below which demand cannot be met for PCR-only (same threshold as dual strategy) and RDT-only. Solid grey and black lines (left panels, **a,d,g,j**) indicate, respectively, where the outcomes of PCR-only and RDT-only are equivalent, and where the outcomes of dual (RDT and PCR) testing and RDT-only are equivalent. Those lines delimit parameter space where (1) dual strategy is best followed by PCR-only and then RDT-only, (2) dual strategy is best followed by RDT-only and then PCR-only and (3) RDT-only is best followed by dual strategy and then PCR-only. On the left of the white solid vertical line (specific for the testing the benefit of care is sufficient to decrease the average CFR among patients seeking care (unaware of their disease status, and assuming hospital infection control has not improved over the course of the epidemic). The black arrows on the right *y*-axis of the RDT-only plots indicate the likely availability of beds at the corresponding stage of the epidemic (Table 2); however, this is likely to have varied between different health-care units.

From a patient perspective, hospitalization is not necessarily optimal and a trade-off exists between the risk of nosocomial infection and the benefits of treatment. At different stages of the epidemic and for the three testing strategies, Figure 2 shows the average CFR per person with suspected Ebola who seeks care as a function of the CFR reduction in the confirmed ward and of the ratio of bed capacity to patients seeking care per day. For hospitalization to be beneficial, the CFR among patients seeking care must be sufficiently reduced by treatment to compensate for the risk of nosocomial transmission to patients without Ebola (Fig. 2). When most patients seeking care are true Ebola cases, a small benefit of treatment is sufficient to reduce the average CFR per patient: at the peak (*p* = 0.7) using the dual strategy, CFR with treatment must fall below 97% of its community value (*r* = 0.97) to decrease the average CFR among patients seeking care (Table 2 and Fig. 2e). When most patients seeking care do not have Ebola, a higher benefit of treatment is required to reduce the average CFR per patient: at the tail of the outbreak (*p* = 0.1) using dual strategy, CFR with treatment must fall below 86% of its community value (*r* = 0.86) to decrease the average CFR among patients seeking care (Fig. 2k). We first consider the impact of testing strategies when the demand for beds does not exceed capacity (Fig. 2). When hospitalization has little impact on CFR, the dual strategy is preferred as it reduces nosocomial transmission. However, RDT-only may become preferable (for example, at the tail of an epidemic; Fig. 2j–l) if the health-care-unit CFR is sufficiently lower than the community CFR such that the higher level of nosocomial transmission in the holding wards seen for the dual strategy outweighs the impact of increased nosocomial transmission in the confirmed wards seen for RDT-only, given that the patients in the latter will benefit from care.

When demand for beds exceeds capacity (Fig. 2), the same reasoning applies and RDT-only is increasingly favoured as it allows a larger proportion of cases to be admitted compared with the other strategies.

For realistic parameter values, we estimate that the RDT-only strategy would be marginally optimal during the growing phase of an epidemic, whereas the dual-testing strategy would be marginally optimal in the later stage (Fig. 2, Table 2, Supplementary Table 3). For instance, during the peak of the epidemic, we assume 60 patients seek care daily and 200 beds are available (resulting in 3.3 beds per patient seeking care), a level of capacity that results in 40% of patients being turned away from the health-care units for the PCR-only (and dual) strategy, compared with 32% patients turned away using RDT-only. Furthermore, if we assume a 30% decrease in CFR for those patients sent to the confirmed ward (*r* = 0.7), the CFR among people with suspected Ebola seeking care (relative to community CFR) would be reduced by 15% under the RDT-only strategy and by 14% under the dual strategy (Table 2, Fig. 2, Supplementary Table 4 for further sensitivity analyses).

**Figure 3:**
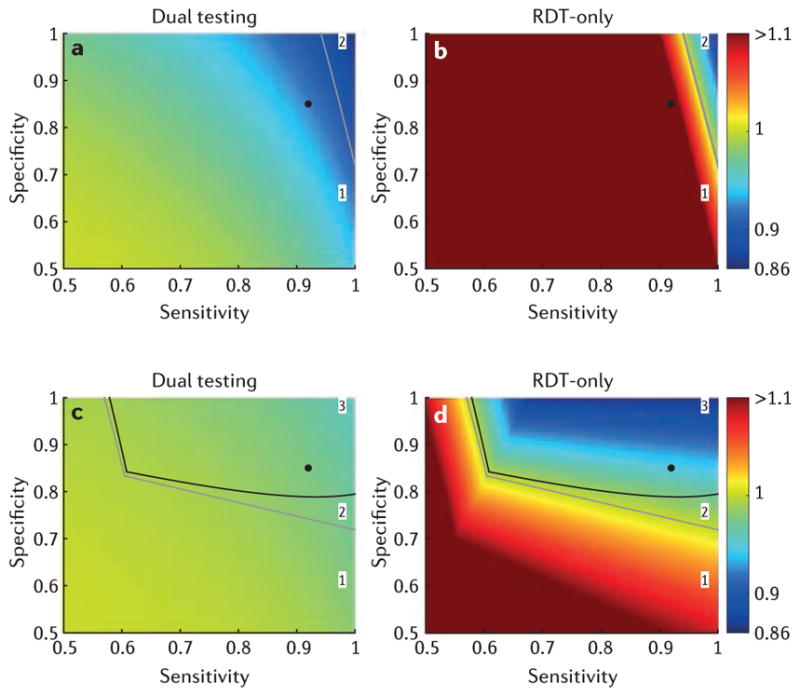
Relative reproduction number of patients with Ebola who are seeking care for dual strategy and rapid diagnostic test (RDT)-only, compared to polymerase chain reaction (PCR)-only, at the peak of the epidemic (see Table 2 for parameters). For **a,b** bed capacity is unlimited, whereas for **c,d** the health-care unit has 200 beds (Table 2). The outcome using a dual (RDT and PCR) strategy is shown in **a**,**c**, whereas **b,d** present the outcome using RDT-only. For the specified parameters and when bed capacity is unlimited, the reproduction number for the PCR-only strategy is 0.53 (0.99 when bed capacity is limited to 200). The PCR-only outcome is independent of the RDT’s sensitivity and specificity. Solid grey and black lines indicate, respectively, where the outcomes of PCR-only and RDT-only are equivalent, and where the outcomes of dual (RDT and PCR) testing and RDT-only are equivalent. Those lines delimit parameter space where (1) dual strategy is best followed by PCR-only and then RDT-only, (2) dual strategy is best followed by RDT-only and then PCR-only and (3) RDT-only is best followed by dual strategy and then PCR-only. The black circle indicates the World Health Organization reported sensitivity and specificity of the ReEBOV RDT (92% and 85%, respectively)^15^.

Figure 3 shows the impact of introducing RDTs on the transmission by a person with Ebola who seeks care for a scenario that is comparable with that in many affected areas at the peak of the recent West African epidemic (see Supplementary Figs 1–4 for results at other stages of the epidemic). We evaluate the dual testing (Fig. 3a) and RDT-only (Fig. 3b) strategies relative to the impact of PCR-only testing.

When bed capacity exceeds demand, the results illustrate some key conclusions about the impact of RDTs on Ebola transmission: transmission is always lowest for the dual strategy, with a reduction in the reproduction number of up to 14% compared with PCR-only (Fig. 3a, Table 2); depending on the RDT’s sensitivity and specificity, the RDT-only strategy may result in lower transmission than the PCR-only strategy (Fig. 3b, Table 2). Low RDT sensitivity worsens the predicted outcome of the RDT-only strategy much more than the outcome of the dual strategy (Fig. 3a,b).

When bed capacity cannot meet demand, the RDT-only strategy may give lower transmission than both the PCR-only and the dual strategies (Figs 3c, d, Table 2). Relying on RDTs alone is optimal during the peak of the epidemic owing to the strategy’s better use of bed capacity (Table 2, Fig. 3c,d, Supplementary Information 2.3, Supplementary Figs 2–5). The benefit of the RDT-alone strategy increases as the level of infection control in the health-care unit decreases (for example see Supplementary Table 6 for results with the rate of nosocomial transmission set at 0.1 and 0.2 per day). Although the reduction in the reproduction number may seem modest during the growing phase of the epidemic (under 11%; Table 2, Supplementary Fig. 1), a reduction of this size can have a considerable impact on cumulative case numbers.

### Potential impact of RDTs at the population level

**Figure 4:**
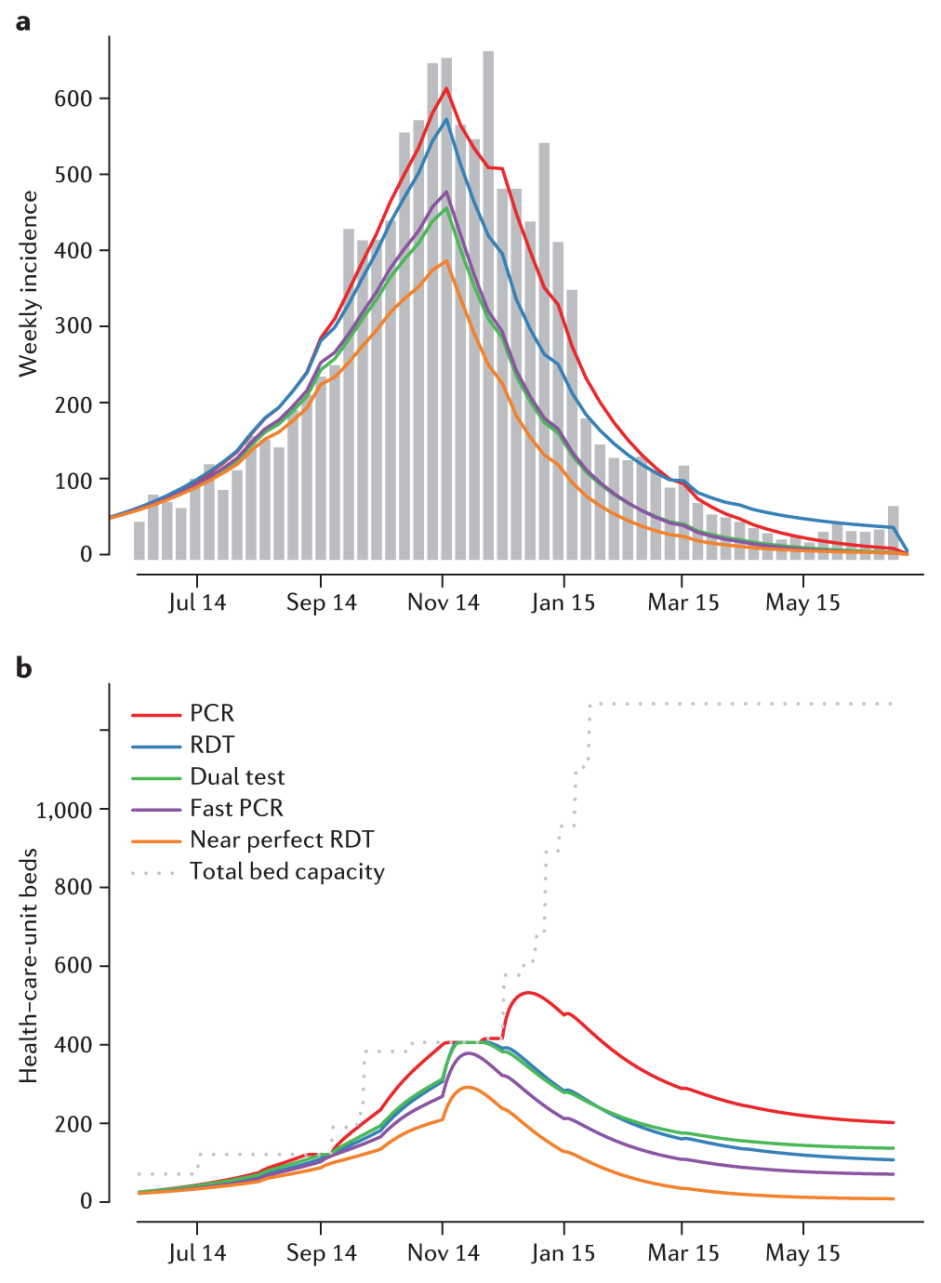
Ebola outbreak in Sierra Leone. **a**, Observed (grey bars) and expected (coloured lines) weekly incidence of confirmed and probable Ebola cases during the outbreak in Sierra Leone. The red line presents the expected incidence using the polymerase chain reaction (PCR)-only strategy on which the model was calibrated. Other lines present the estimated incidence under counterfactual scenarios: rapid diagnostic test (RDT)-only (blue), dual strategy (green), PCR-only with faster test results delivered within 1 day (purple) or RDT-only with near-perfect sensitivity and specificity of 99% each (orange). **b**, Bed capacity (grey) and usage (colours) throughout the epidemic in Sierra Leone for the same scenarios as in (**a**).

We achieve a good match between observed incidence in Sierra Leone and the population-level transmission model after calibration, assuming PCR-only testing (Fig. 4a). We then use the model to evaluate the potential impact of RDTs (Fig. 4, Table 3). We estimate that adopting an RDT-only strategy would have reduced the size of the epidemic by 6%, despite imperfect sensitivity leading some patients with Ebola to be discharged back to the community and continuing to transmit infection there, particularly in the tail of the outbreak. The number of patients with Ebola discharged from hospital is similar under both the PCR-only and RDT-only strategies; for the PCR-only strategy, these are nosocomial infections, whereas for the RDT-only strategy they are predominantly cases with false-negative test results. When demand for beds exceeds capacity, the quick turnaround time of the RDT considerably reduces the need for hospital beds, and consequently fewer patients are turned away from hospital (Fig. 4b and Supplementary Fig. 8). Thus, RDT-only might be the preferred testing strategy, particularly when bed demand is exceeded.

However, with the limited sensitivity and specificity of existing RDTs, adopting a dual strategy would have been more effective than relying on either PCR- or RDT-testing alone. We estimate that such a dual strategy could have reduced the size of the epidemic by 32% compared with the PCR-only strategy (Fig. 4, Table 3). Note that the precise reduction in case numbers achieved crucially depends on the risk of nosocomial transmission. The segregation of cases into high- and low-risk wards sufficiently reduces nosocomial transmission to substantially reduce the number of newly infected cases being discharged back to the community. This more efficient disruption of transmission within both health-care-unit and community settings leads to lower incidence early in the epidemic, and consequently a lower bed demand throughout, reducing the time periods when bed demand exceeds capacity (Fig. 4b, Supplementary Fig. 9).

Reducing the average delay in obtaining PCR test results from 2 days to 1 day could have reduced overall case numbers by 30% without recourse to RDTs (Fig. 4, Table 3). The impact of faster PCR testing is twofold: a reduction in the average stay in health-care units reduces demand for beds in the holding wards, and reduces nosocomial transmission.

If near-perfect RDTs were available (with both sensitivity and specificity at 99%), we estimate that the epidemic in Sierra Leone could have been reduced by 42% — substantially better than even 1-day-turnaround PCR testing could achieve. In this scenario, the duration of health-care-unit stay for people who are suspected of having, but are not infected with, Ebola is minimal, implying a considerably lower demand for beds (Fig. 4b). The near-perfect RDT generates a very small number of misdiagnoses, therefore reducing both the numbers of false-negative discharges and false-positive admissions to the confirmed ward — saving available beds for patients truly infected with Ebola (Supplementary Fig. 9).

Overall, model predictions of the epidemic size are highly sensitive to the assumed values of RDT sensitivity and specificity for the RDT-only strategy, ranging from a 42% reduction for a near perfect test to nearly a twofold increase if sensitivity and specificity are very poor. However, model predictions for the dual strategy are much less sensitive to the RDT characteristics, with the reduction in epidemic size only varying in the range 27% to 41% (see Supplementary Information 3.4).

Sensitivity analyses that assume lower PCR and RDT sensitivity and specificity (owing to challenges in sample-taking and transport) give qualitatively similar results (Table 3).

## DISCUSSION

Our results support the WHO advice on the use of RDTs, showing that RDTs could improve the control of Ebola epidemics, particularly in contexts in which laboratory or bed capacity is limited compared with demand, and infection control between patients in health-care units is imperfect. Implementing a dual testing strategy (whereby RDTs are used for early triage into high- and low-risk holding areas, followed by confirmation by PCR before admission to confirmed wards or patients are discharged) has the potential to decrease nosocomial transmission, leading to a sizeable reduction in the final epidemic size.

Crucial to the assessment of the benefits of RDTs in Ebola control is the ever-present risk of nosocomial transmission. Even when health-care-unit bed capacity can meet demand, a trade-off exists between using a slow PCR test and relying solely on RDTs. The longer wait for PCR test results increases the risk that suspected patients who are not actually infected with Ebola will become infected in holding areas. However, relying on RDTs alone has two potential negative consequences: false positives due to imperfect specificity, which leads to increased nosocomial transmission as non-Ebola patients are sent to the confirmed ward; and false negatives due to imperfect sensitivity, leading to discharge of infected individuals back to the community. When most people with suspected Ebola seeking care are infected (as might be expected in the growth phase of an epidemic), high sensitivity is the more important of these two factors to reduce the risks of discharging false negatives. When most people with suspected Ebola are not infected with Ebola (as expected in the tail of an epidemic) high specificity is needed to minimize the number of false positives. As nosocomial transmission risks increase (for example, due to high demand on services, overcrowding or limitations of infection control), so do the benefits of adopting RDTs in combination with PCR testing.

The prevalence of Ebola infection in patients seeking care and the risk of nosocomial transmission crucially determine the optimal testing strategy. Intermediate values of the underlying prevalence of Ebola infection among those seeking care give the highest risk of nosocomial transmission by maximizing contact between patients with and without Ebola.

Although the average bed occupancy per suspect case is the same for the PCR-only and dual strategies, the RDT-only strategy affects bed usage: less time is spent awaiting test results whereas the proportion of patients sent to the confirmed ward is determined by RDT sensitivity and specificity. Here, the combination of high sensitivity and specificity is of course optimal — a poor specificity of RDT may increase overall bed occupancy by causing increased numbers of patients without Ebola to be falsely confirmed with Ebola and admitted to the confirmed wards.

During the current epidemic there were periods in which bed demand exceeded capacity. In such circumstances, our analysis suggests that relying on RDTs alone could substantially improve utilization of bed capacity, despite less than perfect diagnostic sensitivity and specificity. We estimate that using the RDT-only strategy throughout the epidemic would have decreased case numbers by a modest 6% in Sierra Leone. This is because the better bed utilization under this strategy is counterweighed by the higher false-positive rate of current RDTs compared with PCR; this becomes more of an issue in the tail of an epidemic during which we expect the prevalence of true Ebola infection among people with suspected Ebola to decline. However, the RDT-only strategy would be a more attractive option if infection control in health-care units could be increased in the tail of an epidemic, mitigating this negative effect.

The dual strategy using both PCR and RDT was predicted to have the greatest impact on epidemic size, potentially reducing the size of the epidemic in Sierra Leone by a third. A similar reduction could have been achieved with PCR testing alone if results had been consistently reported back to health-care units within 24 hours. However, as the logistical challenges involved in setting up rapid, high-throughput PCR testing close to the point of care are substantial, RDTs are likely to be a useful addition to the diagnostic armoury for combatting future Ebola epidemics.

Our models inevitably simplify the complex processes underlying Ebola transmission dynamics and patient testing and treatment. However, they provide an important initial evaluation of the potential impact of RDT use on outbreak control. Our qualitative conclusions were robust to sensitivity analyses, and although the numerical predictions of reductions in epidemic size should be cautiously interpreted, the benefit of using RDTs in combination with PCR testing remained. In the baseline population level analysis, we assumed perfect sensitivity and specificity of PCR testing, but logistical challenges in field conditions might lead to substantially lower real-world performance^16,22^. However, we found that the predicted qualitative impact of RDTs was robust to possible suboptimal sensitivity and specificity of PCR tests (Table 3).

Furthermore, there is considerable uncertainty in RDT performance, with reported sensitivity and specificity for ReEBOV varying between 78% and 100%, and 85% and 92%, respectively^14–16^. Such differences would have substantial epidemiological and clinical consequences should an RDT-only testing strategy be adopted. This uncertainty also limits our ability to assess the potential impact of RDTs, particularly if used alone. The predicted impact of dual-testing strategies is less sensitive to uncertainty in RDT performance, with all reported values for ReEBOV resulting in a considerable reduction of the epidemic size (between 27% and 41%).

Finally, for the main analysis presented here, we conservatively assumed that RDTs would be less accurate than PCR tests. Although this is true for ReEBOV^14,15^ (Supplementary Table 1), some alternative RDTs in development seem to be as accurate as their PCR counterparts, even when field tested (for example, REVAMP^23^; J.-C. Manuguerra, personal communication; Supplementary Table 1). Such tests could have a dramatic impact on the control of future epidemics in which health care or laboratory capacity is limited (Table 3). Therefore as innovation in RDT development continues, RDTs could quickly replace PCR testing, achieving similar accuracy with the benefit of faster results, simpler logistics and safer handling of isolates. This article has evaluated a limited set of diagnostic testing strategies for Ebola and further work should evaluate alternative diagnostic methods tailored to field procedures. In Sierra Leone, the epidemic response was decentralized with each district hosting an Ebola response centre, which investigated local cases based on contact tracing, potential exposure and local knowledge. In such settings, an alternative testing strategy could see RDTs used in the community. This could reduce bed demand, costs, patient-transportation needs and risks of transmission. Furthermore, such community use of RDTs could potentially identify cases that would not have met clinical case definitions, and would send a positive message to the community that the testing is fast and transparent.

In the recent West African Ebola epidemic it has been difficult to obtain information about the changes over time in funeral practices, care-seeking behaviour, bed capacity and population mobility. In addition, the level of case ascertainment is uncertain, but is likely to have been poor at certain times in some areas^11,24,25^. These data gaps may be at least partly filled if priority is placed on collation of the wealth of local knowledge and data that may otherwise become lost as the people who contributed to the response resume other activities.

Updating standard diagnostic and triage procedures for Ebola to include the use of RDTs could offer substantial benefits both from a patient and health-care-unit perspective, and in improving overall control of an epidemic. Until now, Ebola outbreaks were thought to be easily controlled. The usual narratives surrounding the control of Ebola highlight the importance of safe funerals, prompt isolation and effective contact tracing. However, testing strategies can also have a crucial role in minimizing the opportunity for nosocomial transmission and maximizing bed utilization. The ease of use of RDTs, particularly in a resource-poor setting, makes them potentially powerful tools for rapid detection and containment of future outbreaks.

## Supplementary Material

Supp1

Supp2

Supp3

## Figures and Tables

**Table 1:** Characteristics of diagnostic tests that have been approved by the World Health Organization.

Test name	Detected	Sensitivity	Specificity	95% limit of detection	Time to result	Principal logistic challenge	Cost
RealStar Filovirus^3,13^*	Ebola-specific RNA	NR	NR	1,390 RNA cps ml^−1^ (95% CI = 690–5,320)	Hours	Kit is shipped on dry ice and should arrive frozen and be kept at −20 °C; needs equipment, including an appropriate PCR machine; needs special training; and needs high safety level in the laboratory	NR
ReEBOV Antigen Rapid Test^14–16^*	Ebola virus (EBOV) VP40 antigen	91.8% (95% CI = 84.5–96.8) compared with RealStar^15^	84.6% (95% CI = 78.8–89.4) compared to RealStar^15^	6.25E+02 ng ml^−1^ Ebola rVP40 antigen	15 minutes	Requires refrigeration at 2–8 °C, requires visual interpretation, should be used in biosafety level 4 facility or with full PPE and can use whole blood from finger prick or venipuncture	NR
Xpert^26,27^*	Ebola-specific RNA	For Ebola Mayinga RNA: 100%, (95% CI = 92.9–100.0) (PPA)	For Ebola Mayinga RNA: 100.0%, (95% CI = 92.9–100.0) (NPA)	232.4 RNA cps ml^−1^ (95% CI = 163.1– 301.6)	90 minutes	Requires refrigeration at 2–8 °C, optimally should be used in a class II safety cabinet or similar, needs special training, automated process and requires a minimum of 100 μl whole blood by venipuncture	US$19.80 per cartridge
LifeRiver^4,28^	Ebola-specific RNA	1 log10 lower limit of detection than RealStar	NR	23.9 RNA cps per reaction (95% CI = 13.4–405.9)	Results in 2 hours, total processing 4–6 hours	Reagents must be kept at −20 °C; needs equipment, including an appropriate PCR machine; needs special training; needs high safety level in the laboratory	NR

* Test is also US Food and Drug Administration (FDA) authorized. All sensitivity, specificity and limits of detection are reported for Zaire EBOV unless otherwise stated.

Cps, copies; NPA, negative per cent agreement; NR, not reported; PCR, polymerase chain reaction; PPA, positive per cent agreement; PPE, personal protective equipment.

**Table 2:** Illustration of health-care-unit model predictions for the CFR among patients seeking care (relative to the community CFR) and for the reproduction number of true Ebola patients seeking care for three testing strategies for levels of bed demand and true Ebola infection prevalence appropriate for four different stages of the current epidemic, as informed by the calibrated parameters of the population-level transmission model.

Test name	Early			During the peak			Decreasing			Going to zero		
	PCR-only	Dual	RDT-only	PCR-only	Dual	RDT-only	PCR-only	Dual	RDT-only	PCR-only	Dual	RDT-only
*v* (daily rate of arrival of patients)‡	5			60			60			60		
*p* (%, prevalence)‡	90			70			50			10		
Community reproduction number‡	1.7			1.7			0.85			0.85		
CFR among patients seeking care*	0.73	0.72§	0.74	0.79	0.76§	0.78	0.85	0.81§	0.84	0.98	0.96§	1.00
*R*_HU_*	0.48	0.47§	0.56	0.53	0.49§	0.59	0.36	0.28§	0.37	0.49	0.4§	0.75
Bed demand	33		30	330		290	270		226	150		97
Bed capacity†, ‡	30			200			350			350		
CFR among patients seeking care†	0.75	0.74	0.74§	0.87	0.86	0.85§	0.85	0.81§	0.84	0.98	0.96§	1.00
*R*_HU_†	0.57	0.56§	0.56	0.99	0.96	0.94§	0.36	0.28§	0.37	0.49	0.4§	0.75

* Results assuming bed capacity (total number of beds in health-care unit) exceeds demand.

† Results assuming health-care-unit bed numbers are limited. For the CFR results, we assume hospitalization decreases the CFR of patients admitted to the confirmed ward by a factor of 0.7.

‡ Assumed model parameters.

§ The optimal strategies. Further model parameters are fixed at their baseline values (Supplementary Tables 4 and 6 show equivalent results with lower and higher nosocomial transmission rate).

CFR, case fatality ratio; PCR, polymerase chain reaction; RDT, rapid diagnostic test.

**Table 3:** Summary statistics of the different testing scenarios considered. The upper rows assume test performance as in the baseline scenario, whereas the lower rows assume real-world use leads to a reduction in test performance as described in the methods.

Scenario		Total number of cases (%relative to PCR-only)	Peak weekly incidence	Total number of infected patients discharged	Total number of Ebola cases turned away from health- care units due to lack of beds
Perfect PCR and nominal RDT characteristics	PCR-only	11,600 (100)	620	780	490
	RDT-only	10,800 (94)	580	960	60
	Dual testing	7,900 (68)	460	280	40
	Fast PCR	8,100 (70)	480	320	0
	Near perfect RDT-only	6,700 (58)	390	70	0
‘Real world’ PCR and RDT characteristics	PCR-only	11,500 (100)	630	1,820	710
	RDT-only	10,700 (93)	600	1,760	260
	Dual testing	9,100 (79)	520	1,270	290
	Fast PCR	8,800 (77)	540	1,260	70

PCR, polymerase chain reaction; RDT, rapid diagnostic test
