# Supplementary material for: The role of rapid diagnostics in managing Ebola epidemics
Source: Nature. Author manuscript; Available in PMC 2016 Apr 6. (PMC4823022; doi:10.1038/nature16041)
Supplement: Supp1 [file NIHMS773804-supplement-Supp1.pdf]

Tools to assess the potential impact of RDTs on Ebola epidemic control at the Healthcare Unit level

Supplementary Information - Tools

Table of Contents

1 Introduction..2

2 Microsoft Excel-based tool..3

3 Java based tool 6

4 Parameter values and important caveats when using the tool..............11

1 Introduction

In this supplementary material, we present two operationalised tools reproducing the results of the 'Potential impact of RDTs on Ebola epidemic control at the Healthcare Unit level' section of the main text (both the patient and the transmission perspectives). The Excel and java-based tools may be used within ETUs and other healthcare settings to inform the choice of a 'local' testing strategy. The model requires specification of the rate of patients presenting for care (ν), their prevalence (p), typical time to obtain results (τ_1 and τ_2), which could be based on earlier monitoring, and the RDT sensitivity and specificity. Additionally, an assumption is required for the rate of nosocomial transmission (β, see section SI.2.4 below). For the patient perspective, the tools require specification of the Case Fatality Ratios (CFRs) among patients seeking care (–true Ebola as well as non-Ebola cases). For the transmission perspective, the tools require a reproduction number among true Ebola cases who do not seek care (Community reproduction number), an average duration of infectiousness, the average delay between symptom onset and hospitalisation and the average time spend in an HU for confirmed cases.

The model presented in Figure 1, and in more details in the SI.1, allows us to measure the outcomes of interest with three distinct diagnostic strategies:

1. PCR-only: patients await their test results in a single holding area. When test results are available, individuals are either sent to a confirmed ward or discharged back to the community.
2. Dual strategy (RDT & PCR): based upon initial RDT results, patients seeking care are kept in separate low- or high-risk wards. When PCR test results become available, they are then sent to a confirmed ward or discharged back to the community.
3. RDT-only: the RDT result alone determines who is sent to confirmed ward or discharged back to the community.

The 'patient perspective outcome' is measured as the Case Fatality Ratio (CFR) among patients seeking care. The 'transmission perspective outcome' is measured as the reproduction number among true Ebola patients seeking care.

2 Microsoft Excel-based tool

	Parameters	
	Average time of rapid test (in hours)	1
	Average time of PCR test (in days)	2
	Daily number of suspected Ebola cases seeking care	60
	Prevalence of infection among those cases (%)	70
	Rate of transmission (per day)	0.15
	Average duration from hospitalisation to discharge	7
	Sensitivity of rapid test	0.92
	Specificity of rapid test	0.85
	Bed capacity (total)	200
For patients perspective	CFR ebola	0.6
	CFR for non-Ebola illness mistaken as Ebola	0.2
	Reduction in CFR with care	0.7
For transmission perspective	Community reproduction number	1.7
	Average duration of infectiousness	15
	Delay between symptom onset to hospitalisation	4

Evaluation of simple HU model - patient perspective

	Testing strategy	CFR among patients seeking care	CFR relative to community	Number of patients dying: Among initally Ebola-infected: In confirmed ward	In community (due to bed limitations issues)	Iin community (due to imperfect sensitivity of RDT)	Among initially Ebola-uninfected (in bracket: from Ebola): In community after testing	In community (due to bed limitations issues)	In confirmed ward (due to imperfect RDT specificity)	In community after being discharged from confirmed ward (RDT specificity)	Total dying	Number dying if they stayed in the community
No limit in bed capacity	PCR-only	0.38	0.79	17.64	-	-	5.1 (1.5)	-	-	-	22.74	28.80
	RDT-only	0.38	0.78	16.23	-	2.02	3.09 (0.03)	-	0.45 (0.07)	0.77 (0.77)	22.56	
	Dual	0.37	0.76	17.64	-	-	4.28 (0.68)	-	-	-	21.92	
With limits in bed capacity	PCR-only	0.42	0.87	10.69	9.93	-	3.09 (0.91)	1.42 (0)	-	-	25.13	
	RDT-only	0.41	0.85	11.19	7.83	1.39	2.13 (0.02)	1.12 (0)	0.31 (0.31)	0.53 (0.53)	24.50	
	Dual	0.41	0.86	10.69	9.93	-	2.6 (0.41)	1.42 (0)	-	-	24.63	

Evaluation of simple HU model - transmission perspective

	Testing strategy	Reproduction number among patients seeking care	Reproduction number (relative to PCR)	Contributions to the reproduction number from admitted patients: Initially infected in the community - Infection pre-hospital	Nosocomial infection discharged to the community after testing	Nosocomial infection sent to confirmed ward (false positive)	Initally infected discharged to the community (false negative) - infection post-hospital	Contribution from infected that are never admitted	Proportion of patients admitted	Bed demand
No limit in bed capacity	PCR-only	0.53	1	0.45	0.07	-	-	0.00	1.00	330
	RDT-only	0.59	1.13	0.45	0.00	0.04	0.10	0.00	1.00	290
	Dual	0.49	0.92	0.45	0.03	-	-	0.00	1.00	330
With limits in bed capacity	PCR-only	0.99	1	0.27	0.05	-	-	0.67	0.61	330
	RDT-only	0.94	0.95	0.31	0.00	0.03	0.07	0.53	0.69	290
	Dual	0.96	0.98	0.27	0.02	-	-	0.67	0.61	330

parameter&results

The Excel-based tool requires Microsoft Excel to be installed.

Input Parameters:

The basic input parameters (which are common with the Java tool below) are entered in the grey cells on the left of the worksheet. They include the necessary parameters for the simple model:

- The average time waiting for RDT results, given in hours (equivalent to τ_1 in the main text).
- The average time waiting for PCR results, given in days (τ_2).
- The daily number of patients presenting for care (ν).
- The prevalence of true Ebola infection among those patients, given in % (p).

- The rate of transmission within the HU (β).
- The average delay from hospitalisation to final outcome (τ_3) (here final outcome is either discharge from the confirmed ward or death).
- The sensitivity and specificity of the RDT ($sens$ and $spec$).
- The bed capacity in the HU. Outputs are given assuming bed capacity is unlimited or limited at the specified level.

Additional parameters to obtain the outcome in the patient perspective:

- Both the CFR among true and non-Ebola patients (π_{Ebola} and π_u , respectively).
- The relative reduction of the CFR for patients sent to the confirmed ward and receiving care (r). If $r = 1$ hospitalisation has no impact on CFR, while if $r = 0$ every patient sent to the confirmed ward survives their condition.

Finally, additional parameters to obtain the outcome in the transmission perspective:

- The community reproduction number (R), which corresponds to the reproduction number among Ebola infected cases who do not seek care.
- The average duration of infectiousness (δ_I).
- The delay from symptom onset to hospitalisation (δ_0).

Results

As input parameters are modified, the outcomes (given in the tables on the right) are updated.

The outcome of interest for the patient perspective is the CFR among the patients seeking care and the CFR relative to the CFR among patients who did not seek care (i.e. community CFR, see the last column of the table). We also provide a break-down of the deaths among various categories of patients (e.g. true Ebola, or initially non-Ebola patients). The results are given for each testing strategy and with or without bed limitations. '-' indicates 'not applicable' due to model assumptions. For instance, PCR test is assumed here to be 100% sensitive and specific, therefore, there are no initially non-Ebola patients entering the confirmed ward and the columns N and O are marked as '-' for PCR-only and dual testing.

The outcome for the transmission perspective is the reproduction number among patients seeking care. We provide such reproduction numbers relative to the PCR-only strategy (i.e. the strategy used in the current outbreak). Again these reproduction numbers can be

compared to the reproduction number among patients who did not seek care (i.e. the community reproduction number). We also provide a break-down of contribution from patients who are or are not admitted to the HU for testing (depending on bed capacity); summing those adds up to the reproduction number. Finally, we provide the proportion of patients admitted for testing and the bed demand for the given set of parameters. The table reports the outcomes for each strategy, with and without limits in bed capacity.

For each bed limitation scenario, the best strategy (based on the outcome) is highlighted in green, the second best in yellow and the worst in red.

3 Java based tool

Requirements

The Java-based tool requires a Java 7 Runtime Environment or later, freely available from www.java.com. The software consists of a single small executable JAR file, which can usually be run by double-clicking on it, or from a terminal with the command:

java –jar EbolaModel.jar

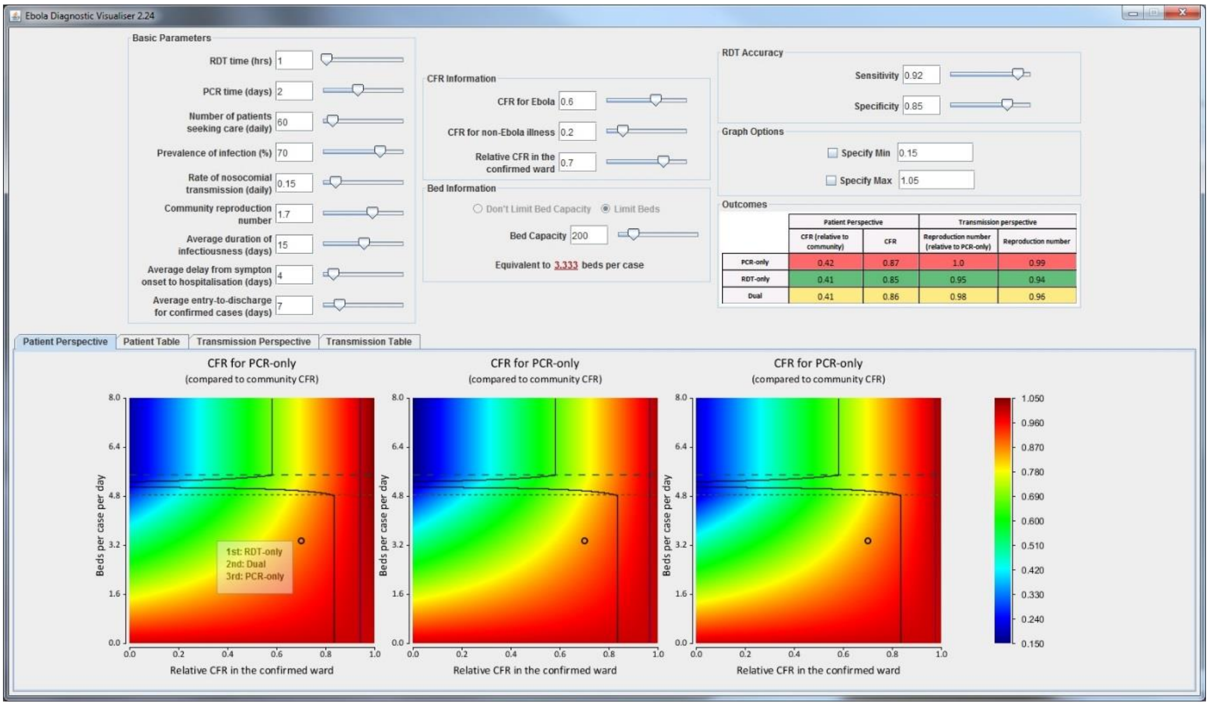

The top half of the interface allows changing input parameters for the model, either by typing values directly into the textboxes, or by using the sliders. The lower half of the interface shows the model outcomes for the given parameter set, either as a heatmap, or as a table much like the excel spreadsheet, for either the patient perspective, or the transmission perspective. When heat maps are being shown, they automatically rescale; this can however be overwritten by specifying a maximum and minimum value under “Graph Options”. We’ll now consider each page in turn.

Patient Perspective:

Here we see heat maps showing the CFR for each strategy (PCR, RDT or Dual), compared to the CFR if infected people stayed in the community and sought no treatment. On the x-axis, we vary the reduction in CFR for patients admitted to the confirmed ward. A value of one indicates the same CFR as in the community, while a value of 0 indicates every patient admitted to the confirmed ward survives their initial condition. When the surface equals 1 (blue vertical line), the CFR among patients seeking care (admitted to HU for testing or not) is the same as the CFR in the community. The black contour lines indicate where the ordering of strategies changes, in terms of which strategy reduces CFR most. Moving the cursor over the colour map shows the ordering of strategies. Left mouseclick to lock onto one place; right-click to unlock it.

As you move the cursor across the heat map, the sliders and values for Relative CFR in HU, and Bed Capacity will update automatically; similarly updating the sliders or text fields for those parameters will change the position of the highlighted point on the heat map. Note that the y-axis (beds per case per day), is the Bed Capacity divided by the daily number of patients seeking care, shown below the Bed Capacity slider. If you change the number of cases per day (third slider from the top left), the Bed Capacity remains the same, but the beds per case will change, as will the highlighted point on the heat map.

As the number of beds per case decreases, bed demand becomes greater than bed capacity. The threshold where bed capacity is reached is shown with the blue horizontal lines. The dashed blue line corresponds to the threshold for PCR-only and dual testing strategy, while the dotted line corresponds to the threshold for RDT-only. Typically, the RDT threshold is lower demonstrating the more efficient use of beds, however if RDT specificity is very low, this trend may be reversed (i.e. due to many uninfected patients being mistakenly sent to the confirmed ward).

Patient Table:

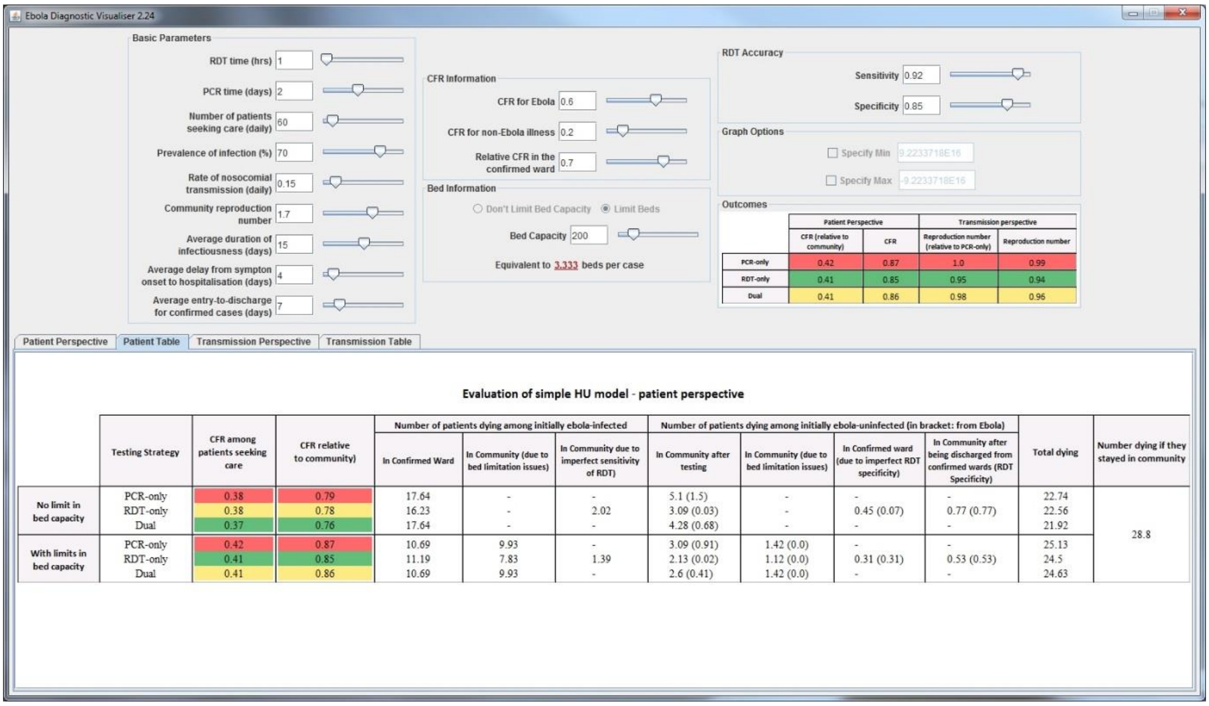

	Testing Strategy	CFR among patients seeking care	CFR relative to community)	Number of patients dying among initially ebola-infected			Number of patients dying among initially ebola-uninfected (in bracket: from Ebola)				Total dying	Number dying if they stayed in community
				In Confirmed Ward	In Community (due to bed limitation issues)	In Community due to imperfect sensitivity of RDT)	In Community after testing	In Community (due to bed limitation issues)	In Confirmed ward (due to imperfect RDT specificity)	In Community after being discharged from confirmed wards (RDT Specificity)		
No limit in bed capacity	PCR-only	0.38	0.79	17.64	-	-	5.1 (1.5)	-	-	-	22.74	28.8
	RDT-only	0.38	0.78	16.23	-	2.02	3.09 (0.03)	-	0.45 (0.07)	0.77 (0.77)	22.56	
	Dual	0.37	0.76	17.64	-	-	4.28 (0.68)	-	-	-	21.92	
With limits in bed capacity	PCR-only	0.42	0.87	10.69	9.93	-	3.09 (0.91)	1.42 (0.0)	-	-	25.13	
	RDT-only	0.41	0.85	11.19	7.83	1.39	2.13 (0.02)	1.12 (0.0)	0.31 (0.31)	0.53 (0.53)	24.5	
	Dual	0.41	0.86	10.69	9.93	-	2.6 (0.41)	1.42 (0.0)	-	-	24.63	

This page is very similar to the Excel Spreadsheet patient perspective page. The values update as you move the sliders, and the colour of the strategies updates to show the best (green), second best (yellow), and worst (red) strategy.

Transmission Perspective:

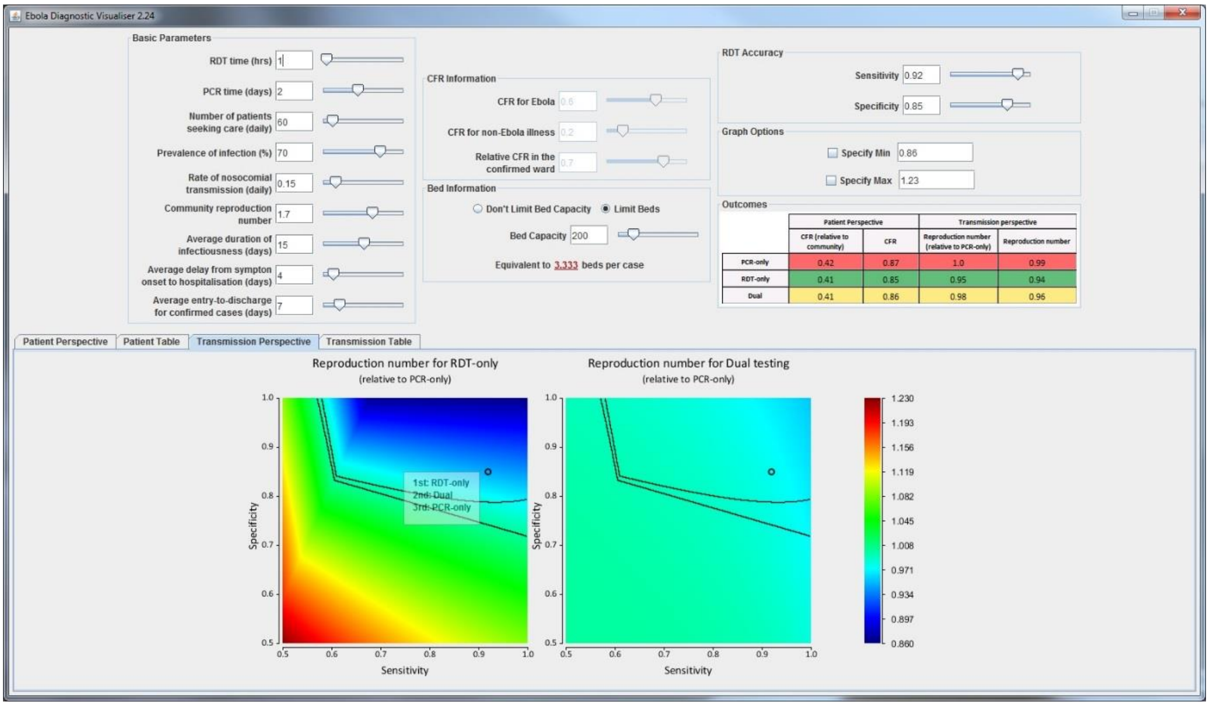

The third tab shows the transmission perspective heat maps. For the counterfactual strategies, RDT-only or Dual, the heat maps show the reproduction number, relative to that of the PCR-only strategy, as a function of RDT sensitivity and specificity. Note here that a value of one implies the same reproduction number as when using the PCR-only testing strategy. Also note that in the screenshot, we have forced the maximum value to be 1.1, as seen in some of the figures in the main paper.

The contour lines again show the boundaries where the ordering of best and second-best strategy changes, as shown as you move the mouse over the heat map.

On this page (and this page only), limits in bed capacity can be switched on or off above the bed capacity slider. Note that on the other pages, this function is disabled: for the patient perspective, the unlimited bed scenario corresponds to the areas above the horizontal blue lines; for both tables, the results are given with and without limits in bed capacity.

Moving the cursor over the image will cause the sensitivity and specificity sliders and text boxes to update to show the current position; again left-click will lock on to a position, and right-click will unlock it, allowing you to move freely around the grid. Changing the sensitive and specificity sliders or typing values will update the cursor position.

The CFR Information is not relevant for these graphs, hence that panel is disabled.

Transmission Table

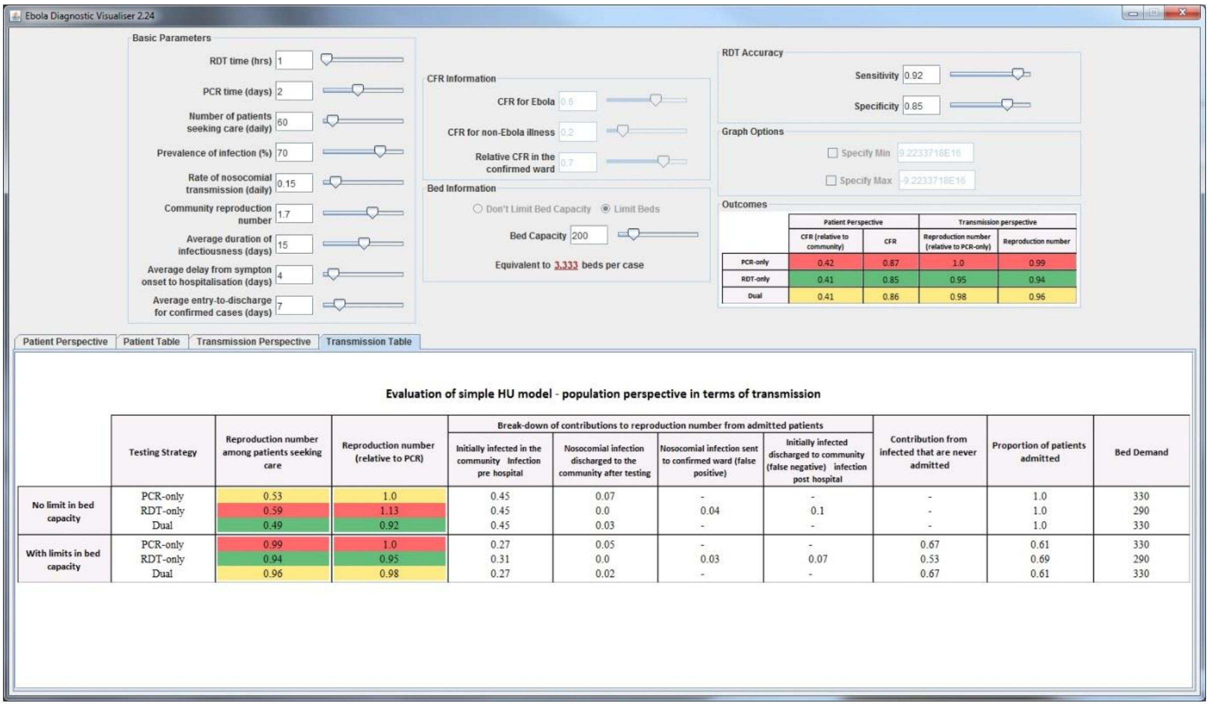

	Testing Strategy	Reproduction number among patients seeking care	Reproduction number (relative to PCR)	Break-down of contributions to reproduction number from admitted patients: Initially infected in the community Infection pre hospital	Nosocomial infection discharged to the community after testing	Nosocomial infection sent to confirmed ward (false positive)	Initially infected discharged to community (false negative) infection post hospital	Contribution from infected that are never admitted	Proportion of patients admitted	Bed Demand
No limit in bed capacity	PCR-only	0.53	1.0	0.45	0.07	-	-	-	1.0	330
	RDT-only	0.59	1.13	0.45	0.0	0.04	0.1	-	1.0	290
	Dual	0.49	0.92	0.45	0.03	-	-	-	1.0	330
With limits in bed capacity	PCR-only	0.99	1.0	0.27	0.05	-	-	0.67	0.61	330
	RDT-only	0.94	0.95	0.31	0.0	0.03	0.07	0.53	0.69	290
	Dual	0.96	0.98	0.27	0.02	-	-	0.67	0.61	330

Finally, the transmission table is again similar to the Excel spreadsheet, letting you see how the outcomes change as you move the sliders to update the values.

4 Parameter values and important caveats when using the tool

The assumed parameter values determine which strategy is preferred and must therefore be chosen carefully. Ideally a range of parameters should be tested to ensure the robustness of the results to the uncertainties in parameter values (and the tools can be used efficiently for that purpose).

The results given in the main text in Figures 2 and 3 and Table 2 assume likely parameter values at the peak of the epidemic, and are the default settings provided in the tools. An important parameter that is difficult to monitor is the rate of nosocomial transmission β. A rough estimate may be obtained from the basic reproduction number R and the average duration of infectiousness d_I, assuming $\beta = R_0 / d_I$ (~0.15 for the Sierra Leone epidemic). The level of infection control within the HU would determine whether β is higher or lower than this estimate. Therefore, some level of qualitative judgment must be made as to whether the local rate of nosocomial transmission is greater or lower than that in the community. Experience of nosocomial transmission in past outbreaks would suggest this value should not be reduced too much (i.e. should remain conservative) as HUs typically will host a large number of infectious patients in confined spaces.

When interpreting the output of the tools, it is important to remember the following limitations:

- The Model does not assess the onward transmission from infected patients who are discharged. For instance, they might have a disproportionally large impact early in the epidemic (see population model in the main text).
- In the patient perspective, the model outcomes rely only on the CFR. However, patients are likely to base their decision on various factors including, but not limited to, their likely CFR. For instance, safeguarding their relatives from infections would likely be part of their 'perspective'.
- In the patient perspective, the model assumes every uninfected patient arrives with the same condition (here severe Lassa fever). While the characteristics of the CFR may be reset using the tool, heterogeneities among patients in condition or in delay to final outcome are not considered.
- The model does not track disease progression while waiting for test results within HUs. However, some initially uninfected patients may progress to the infectious stage

(I) while waiting for test results, and additionally, some initially infected patients may die or recover during their wait for test results. Sensitivity analyses showed the two progressions, i.e. from exposed to infectious and infectious to death or recovery, balanced well and therefore the simple model assumptions (as used) are robust.

- The model does not account for imperfect sensitivity/specificity of PCR-based tests which may occur in field conditions. Therefore the results might be interpreted as relative to PCR sensitivity/specificity.
- The model assumes a total 7 days stay in the HU for all confirmed cases. If the non-Ebola cases were to stay more or less due to longer or quicker recovery, then the bed occupancy would be affected under the RTD-only strategy. This could have an impact especially when most patients seeking care are non-Ebola (i.e. in the tail of the outbreak).
