# Supplementary material for: The role of rapid diagnostics in managing Ebola epidemics
Source: Nature. Author manuscript; Available in PMC 2016 Apr 6. (PMC4823022; doi:10.1038/nature16041)
Supplement: Supp2 [file NIHMS773804-supplement-Supp2.pdf]

The role of rapid diagnostics in managing Ebola epidemics
Supplementary Information

Contents

1 Review of diagnostic tests **2**

2 Health-care-unit-level model **5**
2.1 Introduction and model formulation . . . 5
2.1.1 Equilibrium state of the system . . . 6
2.1.2 Introducing bed capacity limits . . . 8
2.2 Patient perspective . . . 9
2.2.1 PCR-only strategy: Calculating the CFR among patients seeking care . . . 10
2.2.2 Dual testing strategy: Calculating the CFR among patients seeking care . . . 10
2.2.3 RDT-only stragegy: Calculating the CFR among patients seeking care . . . 10
2.2.4 Model output . . . 12
2.3 Transmission perspective . . . 15
2.3.1 Reproduction number using the PCR-only strategy . . . 15
2.3.2 Reproduction number using the dual testing strategy . . . 15
2.3.3 Reproduction number using the RDT-only strategy . . . 16
2.3.4 Accounting for bed capacity . . . 16
2.3.5 Model output . . . 16
2.4 Tools to obtain outcomes: parameter values and important caveats . . . 23

3 Population level model **24**
3.1 Model structure . . . 24
3.1.1 Transmission model . . . 24
3.1.2 Model with hospitalisation: both rapid and PCR tests . . . 25
3.1.3 2-ward model for the PCR-only and RDT-only strategies . . . 30
3.2 Model calibration . . . 31
3.2.1 Calibration of the basic population level transmission model . . . 31
3.2.2 Calibration of the full transmission model with health-care unit structure . . . 33
3.3 Model output . . . 38
3.3.1 Assuming perfect PCR and lab-determined RDT performance . . . 38
3.3.2 Assuming lower PCR and RDT performance in the field . . . 40
3.4 Sensitivity Analyses . . . 43
3.4.1 Uncertainty in RDT characteristics . . . 43
3.4.2 Sensitivity to the duration of symptoms in patients uninfected with Ebola . . . 45
3.4.3 Sensitivity to parameter values . . . 45

1 Review of diagnostic tests

Table SI.1: Characteristics of diagnostic tests available and in development (extension of Table 1 in the main text) as of July 2015. * indicates that the test is also Food and Drug Administration (FDA) authorised. All sensitivity, specificity and limits of detection are reported for Zaire EBOV unless otherwise stated. POC=Point of Care. PFU = Plaque-Forming Units. PPE = Personal Protective Equipment. NR = Not Reported. PPA = Positive Percent Agreement. NPA = Negative Percent Agreement. While sensitivity, specificity and/or limits of detection were usually reported, details such as cost and time were less frequently given.

Test name	Detecting	Sensitivity	Specificity	95% limit of detection	Time to result	Principal logistic challenge	Cost	Reference
**WHO approved**								
**RealStar Filovirus***	Ebola specific RNA	NR	NR	1390 RNA copies/ml (95% CI: 690-5320)	Hours	Kit is shipped on dry ice and should arrive frozen and be kept at -20C; needs equipment, including an appropriate PCR machine; needs special training; needs high safety level in the laboratory	NR	WHO RealStar Report [1], RealStar Manual [2]
**ReEBOV Antigen Rapid Test***	Ebola virus (EBOV) VP40 antigen	91.8% (95% CI: 84.5-96.8) compared with RealStar[3]	84.6% (95% CI: 78.8-89.4) compared to RealStar[3]	$6.25x10^2$ ng/mL Ebola rVP40 Antigen	15 minutes	Requires refrigeration at 2 - 8C. Requires visual interpretation. Should be used in Biosafety Level 4 facility or with full PPE. Can use whole blood from finger prick or venipuncture.	NR	WHO ReEBOV Report [3], ReEBOV Manual [4], Broadhurst et al [5]
**Xpert***	Ebola specific RNA	For Ebola Mayinga RNA: 100.0% (50/50), (95% CI: 92.9-100.0) (PPA)	For Ebola Mayinga RNA: 100.0% (50/50), (95% CI: 92.9-100.0) (NPA)	232.4 RNA copies/ml (95% CI 163.1-301.6)	90 minutes	Requires refrigeration at 2 - 8C. Optimally should be used in a class II safety cabinet or similar. Needs special training. Automated process. Requires a minimum of 100μL whole blood by venipuncture.	$19.80 per cartridge	WHO Xpert Report [6], Xpert Manual [7]
**LifeRiver**	Ebola specific RNA	1 log10 lower limit of detection than RealStar	NR	23.9 RNA copies/reaction (95% CI: 13.4- 405.9)	Results in 2 hours, total processing 4-6 hours	Reagents must be kept at -20C; needs equipment, including an appropriate PCR machine; needs special training; needs high safety level in the laboratory	NR	WHO LifeRiver Report [8], LifeRiver Manual [9]
**FDA authorised for emergency use [10]**								
**EZ1 Real-time RT-PCR Assay**	Ebola specific RNA	Tested on two instruments (1) 100% (45/45) (95% CI: 92.13%-100%) (2) 86.70% (39/45) (95% CI: 73.21%-94.95%) (PPA)	On both instruments: 100% (100/100), (95% CI: 96.38%-100%) (NPA)	at least 1,000 PFU/mL	NR	Controls and kit components must be kept at -20C. Requires class II biosafety cabinet or similar and some specialist laboratory equipment. Needs whole blood or plasma. Needs trained staff.	NR	Ebola Zaire (EZ1) rRT-PCR (TaqMan) Assay Instruction Booklet [11]

Test name	Detecting	Sensitivity	Specificity	95% limit of detection	Time to result	Principal logistic challenge	Cost	Reference
**CDC Ebola Virus NP Real-time RT-PCR Assay**	Nucleoprotein (NP) sequences of EVD	100% (50/50), (95% CI: 93%-100%) (PPA)	100% (50/50), (95% CI: 93%-100%) (NPA)	30 $TCID_{50}$/ reaction	NR	Reagents need to be stored at -20C. Requires whole blood, serum, plasma or urine. Requires specialist laboratory equipment. Needs trained staff.	NR	Ebola Virus NP Real-Time RT-PCR Assay Instructions for use [12]
**CDC Ebola Virus VP40 Real-time RT-PCR Assay**	VP40 matrix protein sequences of EVD	For whole blood and urine: 100% (50/50), (95% CI: 93%-100%) (PPA)	For whole blood: 100% (50/50), (95% CI: 93%-100%) and for Urine: 93.9% (46/49), (95% CI: 83%-98%) (NPA)	30 $TCID_{50}$/ reaction	NR	Reagents need to be stored at -20C. Requires whole blood, serum, plasma or urine. Requires specialist laboratory equipment. Needs trained staff.	NR	Ebola Virus VP40 Real-Time RT-PCR Assay Instructions for use [13]
**BioFire Defense LLC FilmArray Biothreat-E**	Ebola Zaire RNA	For both whole blood and urine: 96.0% (24/25), (95% CI: 80.5%-99.3%) (PPA)	For both whole blood and urine: 100% (25/25), (95% CI: 86.7%-100%) (NPA)	With live virus: $6x10^3$ PFU/ml (blood) and $2x10^4$ PFU/mL (urine)	1 hour	Kit and reagents should be stored at room temperature (15-25C). Requires whole blood or urine. Requires FilmArray System and laptop - fully automated, enclosed system. Needs trained staff.	NR	FilmArray BioThreat-E Instructions for Use [14]
**BioFire Defense LLC FilmArray NGDS BT-E Assay**	Ebola Zaire RNA	Whole blood: 86.7% (39/45), (95% CI: 73.8%-93.7%), plasma and serum: 92% (23/25) (95% CI: 75.0%-97.8%) (PPA)	Whole blood: 100% (50/50), (95% CI: 92.9%-100%), plasma and serum: 100% (25/25) (95% CI: 86.7%-100%)	104 PFU/mL	1 hour	Kit and reagents should be stored at room temperature (15-25C). Requires whole blood, plasma or serum. Requires FilmArray System and laptop - fully automated, enclosed system. Needs trained staff.	NR	FilmArray NGDS BT-E Assay Instructions for Use [15]
**LightMix Ebola Zaire rRT-PCR Test**	Ebola Zaire RNA	97.8% (44/45), (95% CI: 88.4%-99.6%)	100% (100/100), (95% CI: 96.3%-100%)	4,781 PFU/mL	NR	Kit components must be stored at 4-24C and in the dark. Requires whole blood. Requires specialist laboratory equipment and should be conducted in Class II or higher BioSafety Cabinet or similar. Needs trained staff.	NR	LightMix Ebola Zaire rRT-PCR Test Instructions for Use [16]
**OraQuick RDT**	Ebola Virus Antigens	84.0% (21/25), (95% CI: 63.92%-95.46%) (PPA)	98.0% (49/50), (95% CI: 89.35%-99.95%) (NPA)	53,000pg/mL, or 1.06 ng/test (in spiked whole venous blood)	Up to 30 minutes	Can be used with venous or finger prick blood samples. Does not require refrigeration up to 40C, but advised to store at 2-30C. Results read just from number of lines on test stick. Minimal extra equipment required. Appropriate (full) PPE should be worn.		[17]

Test name	Detecting	Sensitivity	Specificity	95% limit of detection	Time to result	Principal logistic challenge	Cost	Reference
**Others reported in the literature**								
**DSTL EVD RDT**	EVD antigen	100% (95% CI: 78.2%-100%) compared with RealStar	96.6% (95% CI: 91.3%-99.1%) compared with RealStar	NR	20 minutes	Uses capillary blood from finger prick. Requires visual interpretation. Can be performed on the ward (with suitable PPE). Minimal training.	NR	Walker et al 2015 [18]
**Matrix Multiplexed Diagnostic (MMDx)**	EVD antigen	NR	NR	150 ng/ml	10 minutes	Requires visual interpretation. Screens for multiple pathogens.	NR	Yen et al 2015 [19], MIT news article [20]
**Ebola eZYSCREEN**	NR	NR	NR	NR	15 minutes	Storage at 4-30C. Does not require any additional lab equipment. Requires visual interpretation. Minimal training. Uses small samples of blood, serum or plasma.	NR	Fierce Diagnostics News Article [21], CEA article [22]
**Rapid Ebola Virus AMPlification (REVAMP)**	Ebola specific RNA	Field evaluation: 100% (95% CI: 96.0% - 100%)	Field evaluation: 100% (95% CI: 95.7%-100%)	NR	30min - 1hr	Currently field laboratory based assays and developing a point-of-care device to automate process. Requires minimal training. Kit can be stored at room temperature. Uses small sample of whole blood.	€3.80/assay for tube format	Targeting Ebola 2015 Abstract [23], Vanhomwegen et al, manuscript in preparation [24]
**Other tests available (not specific)**								
**ELISA antigen capture**	EVD antigen	Approximately 83% (95% CI: 65.3%-94.4%) compared with RT-PCR	In the range of 100% (95% CI: 89.9%-100%) compared with RT-PCR	NR	Hours	Components and samples must be kept cold; needs specific laboratory equipment; needs special training; needs high safety level in the laboratory	Variable	Leroy et al 2000 [25]
**Ebola specific IgM and IgG antibody detection**	EVD specific antibodies	Approximately 67% (95% CI: 47.2%-82.7%) compared with RT-PCR	In the range of 100% (95% CI 89.9-100) compared with RT-PCR	NR	Hours	Components and samples must be kept cold; needs specific laboratory equipment; needs special training; needs high safety level in the laboratory	Variable	Leroy et al 2000 [25]

2 Health-care-unit-level model

Here we present details of the health-care-unit-level model introduced in the main text and the two outcomes associated with the patient and transmission perspectives. We also include the analytical results on which the operationalised tools (**SI.tools**) rely. The schematic figure in Figure 1 of the main text may be translated as three flow diagrams illustrating the three diagnostic strategies explored (see Figure SI.1).

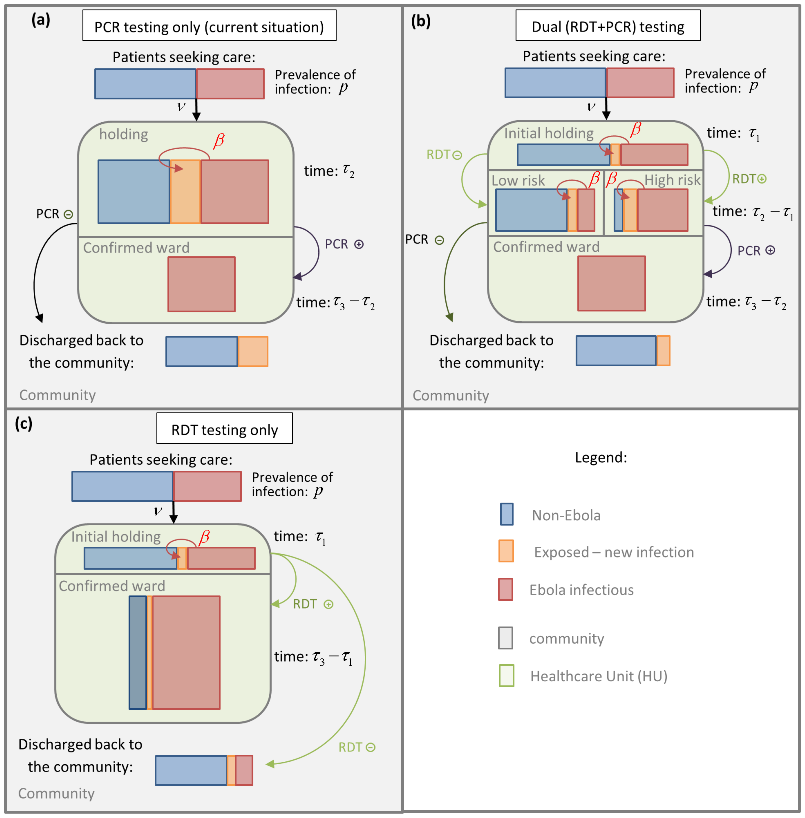

Figure SI.1: Three Ebola health-care unit diagnostic testing and patient triage strategies. Patients are either infected (red), uninfected (blue) or exposed within the holding area (infected but not yet infectious, orange). The height of the coloured rectangles reflects the numbers of beds required in each area, determined in part by the delays in obtaining test results. Curved arrows indicate patient flows and the diagnostic criteria used to move patients between areas.

2.1 Introduction and model formulation

First, we describe the dynamics within the health-care unit using a dual testing strategy. In this strategy the RDT result determines who is sent from the initial holding area to the low- and high-risk wards, while the PCR test is used as a confirmation test to assess whether patients are sent either to the confirmed ward or discharged back to the community.

In the initial holding area, patients seeking care arrive at rate ν, and among those, the prevalence of Ebola infection is p. They remain in this area until the RDT test results arrive (i.e. after $\tau_1 = 1/\eta_1$ on average). During their stay, patients without Ebola, who are susceptibles S, may become exposed (E) at a rate proportional to the rate of within-health-care unit infection (β) and the proportion of patients that are infected (I/N , where $N = S + E + I$) within the holding area. Therefore, like previously published Ebola models [26, 27], we assume frequency-dependent transmission. In this model, only initially infected patients (I_x, x = 'hold', 'high', 'low') are infectious.

Upon receipt of the RDT results, patients are then sent to either the high- or low-risk wards based on their initial status and the sensitivity (sens) and specificity (spec) of the RDT. Therefore some susceptibles may be sent to the high-risk ward, while some infected patients may be sent to the low-risk ward. During their stay, susceptible patients may become exposed by other patients in their ward. On average, patients remain in either ward for a period of $1/\eta_2$, with $\tau_2 = 1/\eta_1 + 1/\eta_2$ being the time from sample collection to result for the PCR confirmation test. Finally, patients initially infected (I_x) are sent to the confirmed ward, while patients initially uninfected with Ebola ($S_\mathrm{x} + E_\mathrm{x}$) are discharged from the low and high risk ward (x = 'high', 'low') back to the community. This implicitly assumes that PCR sensitivity and specificity are perfect.

Initially, we assume no constraint on bed capacity, and the dynamics are given by:

$$\frac{dS_\mathrm{hold}}{dt} = \nu\,(1-p) - \beta\frac{S_\mathrm{hold}I_\mathrm{hold}}{N_\mathrm{hold}} - \eta_1 S_\mathrm{hold} \tag{1}$$

$$\frac{dE_\mathrm{hold}}{dt} = \beta\frac{S_\mathrm{hold}I_\mathrm{hold}}{N_\mathrm{hold}} - \eta_1 E_\mathrm{hold} \tag{2}$$

$$\frac{dI_\mathrm{hold}}{dt} = \nu p - \eta_1 I_\mathrm{hold} \tag{3}$$

in the initial holding area,

$$\frac{dS_\mathrm{high}}{dt} = (1-\mathrm{spec})\,\eta_1 S_\mathrm{hold} - \beta\frac{S_\mathrm{high}I_\mathrm{high}}{N_\mathrm{high}} - \eta_2 S_\mathrm{high} \tag{4}$$

$$\frac{dE_\mathrm{high}}{dt} = (1-\mathrm{spec})\,\eta_1 E_\mathrm{hold} + \beta\frac{S_\mathrm{high}I_\mathrm{high}}{N_\mathrm{high}} - \eta_2 E_\mathrm{high} \tag{5}$$

$$\frac{dI_\mathrm{high}}{dt} = \mathrm{sens}\,\eta_1 I_\mathrm{hold} - \eta_2 I_\mathrm{high} \tag{6}$$

in the high-risk ward, and

$$\frac{dS_\mathrm{low}}{dt} = \mathrm{spec}\,\eta_1 S_\mathrm{hold} - \beta\frac{S_\mathrm{low}I_\mathrm{low}}{N_\mathrm{low}} - \eta_2 S_\mathrm{low} \tag{7}$$

$$\frac{dE_\mathrm{low}}{dt} = \mathrm{spec}\,\eta_1 E_\mathrm{hold} + \beta\frac{S_\mathrm{low}I_\mathrm{low}}{N_\mathrm{low}} - \eta_2 E_\mathrm{low} \tag{8}$$

$$\frac{dI_\mathrm{low}}{dt} = (1-\mathrm{sens})\,\eta_1 I_\mathrm{hold} - \eta_2 I_\mathrm{low} \tag{9}$$

in the low-risk ward.

The subscripts 'hold', 'high', and 'low' indicate the location of the patients as either initial holding, high- and low-risk wards respectively.

This is a simple within-health-care unit transmission model with no disease progression. Implicitly, we assume that those exposed, E, do not become infectious during their health-care unit stay and infectious individuals maintain a constant level of infectiousness and do not die or recover while waiting for their test results. In the population-level model (see Section 3), these assumptions are relaxed.

As we will see below, the outcome for all three different testing strategies can be inferred from the model formulation above.

2.1.1 Equilibrium state of the system

The system above reaches equilibrium very quickly for reasonable parameter values. We therefore focus our analysis on the equilibrium solutions in terms of the three testing strategies used.

At equilibrium (denoted '*'), we have:

$$S^*_{\text{hold}} = \frac{\nu\,(1-p)}{\eta_1 + \beta I^*_{\text{hold}}/N^*_{\text{hold}}} \tag{10}$$

$$E^*_{\text{hold}} = \frac{\beta S^*_{\text{hold}} I^*_{\text{hold}}}{\eta_1 N^*_{\text{hold}}} \tag{11}$$

$$I^*_{\text{hold}} = \frac{\nu p}{\eta_1} \tag{12}$$

in the initial holding area;

$$S^*_{\text{high}} = (1-\text{spec})\,\frac{\eta_1 S^*_{\text{hold}}}{\eta_2 + \beta I^*_{\text{high}}/N^*_{\text{high}}} \tag{13}$$

$$E^*_{\text{high}} = \frac{(1-\text{spec})\,\eta_1 E^*_{\text{hold}} + \beta S^*_{\text{high}} I^*_{\text{high}}/N^*_{\text{high}}}{\eta_2} \tag{14}$$

$$I^*_{\text{high}} = \text{sens}\frac{\eta_1 I^*_{\text{hold}}}{\eta_2} \tag{15}$$

in the high-risk ward;

$$S^*_{\text{low}} = \text{spec}\frac{\eta_1 S^*_{\text{hold}}}{\eta_2 + \beta I^*_{\text{low}}/N^*_{\text{low}}} \tag{16}$$

$$E^*_{\text{low}} = \frac{\text{spec}\,\eta_1 E^*_{\text{hold}} + \beta S^*_{\text{low}} I^*_{\text{low}}/N^*_{\text{low}}}{\eta_2} \tag{17}$$

$$I^*_{\text{low}} = (1-\text{sens})\,\frac{\eta_1 I^*_{\text{hold}}}{\eta_2} \tag{18}$$

in the low-risk ward, with

$$N^*_{\text{hold}} = \frac{\nu}{\eta_1} \tag{19}$$

$$N^*_{\text{high}} = \frac{\nu\,(1-p)\,(1-\text{spec}) + \nu p\,\text{sens}}{\eta_2} \tag{20}$$

$$N^*_{\text{low}} = \frac{\nu\,(1-p)\,\text{spec} + \nu p\,(1-\text{sens})}{\eta_2}\,. \tag{21}$$

Hence:

$$S^*_{\text{hold}} = \frac{\nu\,(1-p)}{\eta_1 + \beta p} \tag{22}$$

$$E^*_{\text{hold}} = \frac{\beta \nu p\,(1-p)}{\eta_1\,(\eta_1 + \beta p)} \tag{23}$$

$$I^*_{\text{hold}} = \frac{\nu p}{\eta_1}\,, \tag{24}$$

in the initial holding area;

$$S^*_{\text{high}} = \frac{(1-\text{spec})\,\eta_1 \nu\,(1-p)}{\left(\eta_2 + \frac{\beta p\,\text{sens}}{(1-p)(1-\text{spec})+p\,\text{sens}}\right)(\eta_1 + \beta p)} \tag{25}$$

$$E^*_{\text{high}} = \frac{1}{\eta_2}\,(1-\text{spec})\,\frac{\beta \nu p\,(1-p)}{\eta_1 + \beta p} A \tag{26}$$

$$I^*_{\text{high}} = \text{sens}\frac{\nu p}{\eta_2}\,, \tag{27}$$

in the high-risk ward;

$$S^*_{\text{low}} = \frac{\text{spec}\,\eta_1 \nu\,(1-p)}{\left(\eta_2 + \frac{\beta p\,(1-\text{sens})}{(1-p)\text{spec}+p\,(1-\text{sens})}\right)(\eta_1+\beta p)} \tag{28}$$

$$E^*_{\text{low}} = \frac{1}{\eta_2}\text{spec}\frac{\beta\nu p\,(1-p)}{\eta_1+\beta p}B \tag{29}$$

$$I^*_{\text{low}} = (1-\text{sens})\,\frac{\nu p}{\eta_2} \tag{30}$$

in the low-risk ward, with $A = \left[1 + \frac{\eta_1 \text{sens}}{\eta_2((1-p)(1-\text{spec})+p\,\text{sens})+\beta p\,\text{sens}}\right]$ and $B = \left[1 + \frac{\eta_1\,(1-\text{sens})}{\eta_2((1-p)\text{spec}+p\,(1-\text{sens}))+\beta p\,(1-\text{sens})}\right]$.

Given these equations, it is then trivial to determine the flow of patients in the health-care unit for the other strategies. For the PCR-only strategy, the flow is as described above with sens and spec set to 50%. For the RDT-only strategy, patients described above as being sent to the high-risk ward are in fact sent straight to the confirmed ward, while patients described above as being sent to the low risk ward are immediately discharged back to the community.

The baseline parameters for the model are given in Table SI.2.

We can then obtain for each testing strategy an outcome reflecting either the patient or transmission perspective.

Table SI.2: Baseline parameters for the model and outcomes.

Parameters	Symbol	Value
For the model		
Average time of rapid test (in hours)	τ_1	1
Average time of PCR test (in days)	τ_2	2
Rate of transmission (per day)	β	0.15
Sensitivity of RDT	sens	0.92
Specificity of RDT	spec	0.85
Average duration from hospitalisation to discharge (days)	τ_3	7
Specific to the patient perspective		
CFR for individuals infected with Ebola not admitted to health-care unit	π_{Ebola}	0.6
CFR for individuals without Ebola not admitted to health-care unit	π_u	0.2
Reduction in CFR with care	r	0.7
Specific to the transmission perspective		
Reproduction number	R	1.7
Average duration of infectiousness	d_I	15
Delay between symptom onset to hospitalisation	δ_0	4

CFR = Case Fatality Ratio.

2.1.2 Introducing bed capacity limits

When bed capacity is limited, we must correct the rate of patients seeking care, thereby defining a lower effective rate of patients being admitted while the remainder are discharged back to the community (i.e. they effectively stay in the community).

We define the maximum testing capacity as $N^{\text{max}}_{\text{tot}}$. Patients discharged back to the community following a negative test (that could be a false negative with RDT-only testing strategy) remain in the health-care unit for a total τ_1 for the RDT-only and τ_2 for both PCR-only and dual testing strategies. The patients sent to the confirmed ward remain in the health-care unit for a total of τ_3 days on average. For the PCR-only and dual testing strateguies all the initially Ebola-infected patients (and only those) are sent to the

confirmed ward. For the RDT-only strategy the patients sent to the confirmed ward include some (but not all) patients initially infected with Ebola and some (but not all) patients initially not infected with Ebola; and the proportion among those two groups depends on the accuracy (sensitivity and specificity) of the RDT. Therefore, we expect the total number of beds in use to be

$$N_{\text{tot}} = \nu \left((1-p)\left(1/\eta_1 \; + 1/\eta_2 \;\right) + p\,\tau_3 \right) \tag{31}$$

for the dual and PCR-only testing strategies; and

$$N_{\text{tot}} = \nu \left[1/\eta_1 \; + \left((1-p)\,(1-\text{spec}) + p\,\text{sens} \right) \left(\tau_3 - 1/\eta_1 \; \right) \right] \tag{32}$$

for the RDT-only testing strategy, with τ_3 the average delay from admission to death/discharge.

Given the bed capacity, the maximum rate of patients seeking care that can be admitted is

$$\nu_{\max} = N_{\text{tot}}^{\max} / \left((1-p)\left(1/\eta_1 \; + 1/\eta_2 \;\right) + p\,\tau_3 \right) \tag{33}$$

for the dual and PCR-only testing strategies; and

$$\nu_{\max} = N_{\text{tot}}^{\max} / \left[1/\eta_1 \; + \left((1-p)\,(1-\text{spec}) + p\,\text{sens} \right) \left(\tau_3 - 1/\eta_1 \; \right) \right] \tag{34}$$

for the RDT-only testing strategy.

The effective rate of patients being admitted becomes $\nu_{\text{eff}} = \min(\nu, \nu_{\max})$, and the system of differential equations (and equilibrium states) above still holds after replacing ν by ν_{eff}.

The outcomes that will be measured must account for the proportion of patients that are not admitted, whose outcomes will be equivalent to the outcomes of individuals that did not seek care. For instance, if there is no bed capacity (i.e. $N_{\text{tot}}^{\max} = 0$), the outcomes calculated below are the same as the outcomes among those who did not seek care (later defined as the community outcomes).

2.2 Patient perspective

For the patient perspective, we introduce some new parameters: the Case Fatality Ratio (CFR) associated with Ebola when the patient does not receive care, π_{Ebola} (community CFR), and the CFR among patients without Ebola seeking care, π_u.

Using the health-care-unit-level model, for each testing strategy, we are able to determine how many infected patients seeking care are sent to the confirmed ward and how many are discharged back to the community (due to bed shortages or false negative diagnosis when using the RDT-only strategy).

Additionally, and again for each testing strategy, we are able to determine how many patients initially without Ebola seeking care were infected during their health-care unit stay and later discharged back to the community. Finally, for the RDT-only strategy, we can also determine the number of patients uninfected with Ebola sent to the confirmed ward where a greater proportion may become infected (i.e. as in the population perspective, see later). Importantly, we assume here that patients discharged after testing will not seek further care from a health-care unit.

We note that hospitalisation in a confirmed ward reduces the CFR by r relative to the CFR of an individual not seeking care (referred to as community CFR). When the infection that patients present with is not Ebola virus, their CFR is assumed to be 20% (as in, e.g., severe Lassa Fever). Only patients in the confirmed ward are assumed to benefit from the relative CFR decrease and this relative decrease applies equally to all patients in the confirmed wards (whether or not they are infected with Ebola). Finally, among patients without Ebola that are sent to the confirmed ward and survive their initial illness, some will become Ebola-infected. If they are infected sufficiently early, they will test positive for Ebola and stay in the confirmed ward (benefitting from reduced CFR) but if they are infected late, they will test negative for Ebola while being in the latent stage of the infection and will be discharged to the community (and have community Ebola CFR).

Given the parameters (including the RDT's sensitivity/specificity, the relative decrease in CFR due to health-care r and the bed capacity $N_{\text{tot}}^{\max}$), we can determine the CFR among those seeking care (infected or not). Therefore, we can consider the patient perspective by calculating the CFR among those seeking care (unaware of their status) compared to the CFR if they did not seek care.

We first define the proportion of patients admitted as a function of the time spent in a health-care unit, the number of patients seeking care and bed capacity. Formally, we have this proportion defined as:

$p_{\text{admitted}} = \frac{\min(\nu, N_{\text{bed}}/\tau_3\ )}{\nu}$, with N_{bed} the bed capacity and τ_3 the average time spend in a health-care unit. Importantly, τ_3 is the same for PCR-only and dual strategies, and is typically shorter for the RDT-only strategy and dependent on RDT sensitivity and specificity, therefore $p^{\text{RDT}}_{\text{admitted}} > p^{\text{PCR \& Dual}}_{\text{admitted}}$ (unless RDT sensitivity and specificity are very poor).

2.2.1 PCR-only strategy: Calculating the CFR among patients seeking care

Among patients seeking care, we derive the number of patients dying. First among those initially infected, the number dying from Ebola will be:

$$D_I = \nu p \times \pi_{\text{Ebola}} \left(r \times p_{\text{admitted}} + (1 - p_{\text{admitted}})\right) . \tag{35}$$

Among those initially not infected with Ebola, the number dying from their initial infection or from Ebola is:

$$D_U = \nu \left(1 - p\right) \pi_u + \left(1 - \pi_u\right) \left[\eta_2 \left(E^*_{\text{low}} + E^*_{\text{high}}\right)\Big|_{\text{spec}=0.5,\,\text{sens}=0.5} \times \pi_{\text{Ebola}}\right] \tag{36}$$

(note the number of nosocomial infections $\eta_2 \left(E^*_{\text{low}} + E^*_{\text{high}}\right)\Big|_{\text{spec}=0.5,\,\text{sens}=0.5}$ accounts for the potential bed limitation).

Therefore the number with unknown infection status dying is $D_I + D_U$ which can be compared to that in the community ($\frac{D_I + D_U}{\nu[p\pi_{\text{Ebola}} - (1-p)\pi_u]}$).

2.2.2 Dual testing strategy: Calculating the CFR among patients seeking care

Among patients seeking care, we derive the number of patients dying. First among those initially infected, the number dying from Ebola will be:

$$D_I = \nu p \times \pi_{\text{Ebola}} \left(r \times p_{\text{admitted}} + (1 - p_{\text{admitted}})\right) . \tag{37}$$

Among those initially not infected with Ebola, the number dying from their initial infection or from Ebola is:

$$D_U = \nu \left(1 - p\right) \pi_u + \left(1 - \pi_u\right) \left[\eta_2 \left(E^*_{\text{low}} + E^*_{\text{high}}\right) \times \pi_{\text{Ebola}}\right] . \tag{38}$$

Again the number with unknown infection status dying is $D_I + D_U$ (both D_I and D_U depend on RDT sensitivity and specificity) which can be compared to the CFR in the community ($\frac{D_I + D_U}{\nu[p\pi_{\text{Ebola}} - (1-p)\pi_u]}$).

2.2.3 RDT-only stragegy: Calculating the CFR among patients seeking care

Among those initially infected seeking care, some will be admitted and correctly sent to the confirmed ward: those will benefit from a potential reduction r in CFR (a proportion $r\pi_{\text{Ebola}}$ will die). As before, some will not be admitted and a proportion π_{Ebola} will die. Additionally, among those admitted, a proportion $1 - \text{sens}$ will be discharged back to the community and a proportion π_{Ebola} will die. The number of infected patients dying from Ebola is:

$$D_I = \pi_{\text{Ebola}} \left(\nu p \left(1 - p_{\text{admitted}}\right) + \left(1 - \text{sens}\right) \eta_1 I^*_h + \text{sens}\, \eta_1 I^*_h\, r\right) . \tag{39}$$

Among those initially not infected with Ebola, the number dying from their initial infection or from Ebola is more complex and is composed of six parts, D^1_U - D^6_U. Among those admitted that do not suffer from nosocomial infection of Ebola, are correctly discharged to the community, but die from their original infection; we have:

$$D^1_U = \text{spec} \times \eta_1 S^*_h \pi_u . \tag{40}$$

Then among those becoming Ebola infected in the holding area and discharged to the community who then die either from their original infection or from Ebola, we have:

$$D_U^2 = \text{spec} \times \eta_1 E_h^* \times [\pi_u + \pi_{\text{Ebola}} (1 - \pi_u)] \ . \tag{41}$$

Among those becoming Ebola infected in the holding area and sent to the confirmed ward who then die either from their original infection or from Ebola, we have:

$$D_U^3 = (1 - \text{spec}) \, \eta_1 E_h^* \times [r\pi_u + r\pi_{\text{Ebola}} (1 - r\pi_u)] \ . \tag{42}$$

Finally, among those patients without Ebola wrongly sent to the confirmed ward, they can:

- Die from their initial condition (4),
- Survive their initial condition and become infected with Ebola, and:
  - Stay in the confirmed ward (5) if, once they have recovered from their initial condition, their 2nd PCR test (for discharge) is performed after the onset of their Ebola symptoms (thus they test positive),
  - Be discharged back to the community (6), if, once they have recovered from their initial condition, their 2nd PCR test (for discharge) is performed before the onset of their Ebola symptoms.

The probability of each situation occurring is:

- $p_4 = r\pi_u$,
- $p_5 = (1 - r\pi_u)(1 - \exp(-\beta p' [\tau_3 - 2 - \delta_0]))$ with δ_0 the incubation period and therefore $[\tau_3 - 2 - \delta_0]$ the last time at which infection can occur for the patient to remain in the confirmed ward,
- $p_6 = (1 - r\pi_u)(\exp(-\beta p' \tau_3)[\exp(\beta p' [2 + \delta_0]) - 1])$.

Additionally, patients without Ebola may survive their initial condition and be discharged to the community uninfected with Ebola. The probability of this occurring is:

$$q = (1 - r\pi_u)\exp(-\beta p' \tau_3)$$

with $p' = \text{sens}\, p / (\text{sens}\, p + (1 - \text{spec})(1 - p))$ the proportion of infected and infectious patients in the confirmed ward.

We can verify

$$q + p_5 + p_6 \, (1 - r\pi_u) \begin{bmatrix} \exp(-\beta p' \tau_3) + \\ 1 - \exp(-\beta p' [\tau_3 - 2 - \delta_0]) + \\ \exp(-\beta p' \tau_3)[\exp(\beta p' [2 + \delta_0]) - 1 \end{bmatrix} = (1 - r\pi_u) \ ,$$

such that $q + \sum_{i=4}^{6} p_i = 1$.

Therefore among those patients without Ebola admitted to a health-care unit, the number dying is $D_U = D_I + \sum_{i=1}^{6} D_U^i$:

$$\begin{aligned} D_U = {} & \nu (1 - p)(1 - p_{\text{admitted}}) \pi_u + \text{spec} \times \eta_1 S_h^* \pi_u + \text{spec} \times \eta_1 E_h^* \times [\pi_u + \pi_{\text{Ebola}} (1 - \pi_u)] \\ & + (1 - \text{spec}) \, \eta_1 E_h^* \times r \, [\pi_u + \pi_{\text{Ebola}} (1 - \pi_u)] \\ & + (1 - \text{spec}) \, \eta_1 S_h^* \times \begin{bmatrix} r\pi_u + (1 - r\pi_u)\exp(-\beta p' \tau_3) \times 0 + \\ r\pi_{\text{Ebola}} (1 - r\pi_u)(1 - \exp(-\beta p' [\tau_3 - 2 - \delta_{\text{inc}}])) + \\ \pi_{\text{Ebola}} (1 - r\pi_u)(\exp(-\beta p' \tau_3)[\exp(\beta p' [2 + \delta_{\text{inc}}]) - 1]) \end{bmatrix} \ . \end{aligned} \tag{43}$$

Additionally, among those not admitted the number dying from Ebola or their original infection is:

$$D_U = D_U^{\text{admitted}} + (\nu - \nu_{\text{eff}})(1 - p)\pi_u$$

Again the number with unknown infection status dying is: $D_I + D_U$ (both D_I and D_U depend on RDT sensitivity and specificity) and can be compared to the CFR in the community ($\frac{D_I + D_U}{\nu [p \pi_{\text{Ebola}} - (1-p)\pi_u]}$).

2.2.4 Model output

If hospitalisation reduces the CFR by a factor $r < 1$, it is preferred among patients infected with Ebola. However, seeking care comes with a cost for patients without Ebola: the risk of nosocomial infection. Thus for individuals without Ebola hospitalisation may increase their CFR, meaning that hospitalisation is not necessarily good for a patient unaware of their infection status. When hospitalisation is not optimal then the relative CFR is greater than one (i.e. a potential patient is more likely to die due to Ebola by seeking care than if he/she had not sought care). Additionally, different testing strategies will incur different costs from a patient perspective and again an optimal testing strategy can be found.

Figure 3 of the main text plots the CFR among patients seeking care along gradients in bed capacity and in impact of hospitalisation on CFR. The outcome measured is the CFR relative to the CFR if no-one had sought care. When health-care unit bed capacity is not reached the CFR among patients seeking care does not depend on bed capacity, however, once there is a shortage of beds, the hospitalised CFR tends toward the community CFR. We evaluated such outcome during four distinct periods of the epidemic using either testing strategy: early, at the peak, shortly after the peak, and when the epidemic is tailing-off (reflecting the situation around June 2014, November 2014, January 2015 and May 2015, respectively). In these scenarios, we assumed that (a) the incidence of patients with Ebola infection increases, peaks and then tails off; (b) the number of patients not infected with Ebola seeking care increases (e.g. due to increasing awareness), and remains high as the epidemic wanes (meaning prevalence of Ebola infection among patients seeking care, p, wanes over time); (c) the reproduction number among those not seeking care (community reproduction number) decreases reflecting improved control measures (e.g. safer funeral practices); (d) bed capacity increases and then plateaus as the epidemic unfolds. These assumptions are in good agreement with our population-level model (see below). These scenarios were used in the analyses using both the patient and transmission perspectives. Specific parameter values can be found in Table SI.3 and SI.5.

In Table SI.3 below we provide detailed results for the four scenarios, by breaking down the contribution to the CFR of patients in the different wards and in community. While CFR between different testing strategies remains relatively stable, the contributions vary. In particular, when the epidemic is tailing off, RDT-only incurs a lower cost due to nosocomial infections discharged to the community, which balanced the nosocomial transmission in the confirmed ward. High specificity of the RDT would reduce such costs.

We evaluated the outcome for each strategy and each period while varying the rate at which infectious patients transmit the virus in health-care unit settings and allowing RDT sensitivity and specificity to vary within the bounds reported in the literature (Table SI.4). The benefit of health-care unit care on patients with (and without) Ebola is unclear and likely differs between health-care units, therefore we also explored the CFR from a patient perspective assuming hospitalisation decreases the CFR by either 30% (baseline, as presented in Table SI.2) or 10%.

	Bed-limits	Testing strategy	ν	p	Bed capacity	CFR (among patients seeking care)	CFR relative to community	Number of patients dying: Among initially Ebola infected: In confirmed ward	In community (bed limitations)	In community (imperfect RDT sensitivity)	Among initially Ebola uninfected (from Ebola): In community (after tested)	In community (bed limitations)	In confirmed ward (imperfect RDT sensitivity)	In community (after discharged from confirmed ward)	Total dying	Number dying if they stayed in the community
Early	No	PCR	5	0.9	-	0.41	0.73	1.89	-	-	0.15 (0.05)	-	-	-	2.04	2.80
		RDT				0.42	0.74	1.74	-	0.22	0.09 (0)	-	0.01 (0)	0.02 (0.02)	2.08	
		Dual				0.40	0.72	1.89	-	-	0.13 (0.03)	-	-	-	2.02	
	Yes	PCR	5	0.9	30	0.42	0.75	1.74	0.21	-	0.14 (0.05)	0.01 (0)	-	-	2.10	
		RDT				0.42	0.74	1.74	0.00	0.22	0.09 (0)	0 (0)	0.01 (0.01)	0.02 (0.02)	2.08	
		Dual				0.42	0.74	1.74	0.21	-	0.12 (0.03)	0.01 (0)	-	-	2.08	
Peak of the epidemic	No	PCR	60	0.7	-	0.38	0.79	17.64	-	-	5.1 (1.5)	-	-	-	22.74	28.80
		RDT				0.38	0.78	16.23	-	2.02	3.09 (0.03)	-	0.45 (0.07)	0.77 (0.77)	22.56	
		Dual				0.37	0.76	17.64	-	-	4.28 (0.68)	-	-	-	21.92	
	Yes	PCR	60	0.7	200	0.42	0.87	10.69	9.93	-	3.09 (0.91)	1.42 (0)	-	-	25.13	
		RDT				0.41	0.85	11.19	7.83	1.39	2.13 (0.02)	1.12 (0)	0.31 (0.31)	0.53 (0.53)	24.50	
		Dual				0.41	0.86	10.69	9.93	-	2.6 (0.41)	1.42 (0)	-	-	24.63	
Decreasing	No	PCR	60	0.5	-	0.34	0.85	12.60	-	-	7.88 (1.88)	-	-	-	20.48	24.00
		RDT				0.34	0.84	11.59	-	1.44	5.14 (0.04)	-	0.74 (0.11)	1.23 (1.23)	20.14	
		Dual				0.32	0.81	12.60	-	-	6.78 (0.78)	-	-	-	19.38	
	Yes	PCR	60	0.5	350	0.34	0.85	12.60	0.00	-	7.88 (1.88)	0 (0)	-	-	20.48	
		RDT				0.34	0.84	11.59	0.00	1.44	5.14 (0.04)	0 (0)	0.74 (0.74)	1.23 (1.23)	20.14	
		Dual				0.32	0.81	12.60	0.00	-	6.78 (0.78)	0 (0)	-	-	19.38	
Tail	No	PCR	60	0.1	-	0.23	0.98	2.52	-	-	11.56 (0.76)	-	-	-	14.08	14.40
		RDT				0.24	1.00	2.32	-	0.29	9.19 (0.01)	-	1.22 (0.09)	1.32 (1.32)	14.35	
		Dual				0.23	0.96	2.52	-	-	11.3 (0.5)	-	-	-	13.82	
	Yes	PCR	60	0.1	350	0.23	0.98	2.52	0.00	-	11.56 (0.76)	0 (0)	-	-	14.08	
		RDT				0.24	1.00	2.32	0.00	0.29	9.19 (0.01)	0 (0)	1.22 (1.22)	1.32 (1.32)	14.35	
		Dual				0.23	0.96	2.52	0.00	-	11.3 (0.5)	0 (0)	-	-	13.82	

Table SI.3: Caption: CFR among patients seeking care at four stages of the epidemic according to testing strategy used. We present estimates of CFR and relative decrease compared to the CFR had they not seek care, as well as individual contributions among various classes of patients. ‘-‘ indicates not-applicable due to model structure. Hospitalisation is assumed to decrease patients’ CFR by a 0.7 factor.

		Bed limits	Early			Peak of the epidemic			Decreasing			Tail		
			PCR	Dual	RDT	PCR	Dual	RDT	PCR	Dual	RDT	PCR	Dual	RDT
sensitivity=92%, specificity=85%	$\beta = 0.15$	No	0.73 (0.92)	0.72 (0.92)	0.74 (0.92)	0.79 (0.96)	0.76 (0.94)	0.78 (0.95)	0.85 (1)	0.81 (0.96)	0.84 (0.98)	0.98 (1.03)	0.96 (1.01)	1 (1.06)
		Yes	0.75 (0.93)	0.74 (0.92)	0.74 (0.92)	0.87 (0.98)	0.86 (0.96)	0.85 (0.96)	0.85 (1)	0.81 (0.96)	0.84 (0.98)	0.98 (1.03)	0.96 (1.01)	1 (1.06)
	$\beta = 0.1$	No	0.72 (0.92)	0.72 (0.91)	0.74 (0.92)	0.77 (0.95)	0.75 (0.93)	0.78 (0.94)	0.83 (0.98)	0.8 (0.95)	0.83 (0.97)	0.96 (1.01)	0.95 (1)	0.97 (1.03)
		Yes	0.75 (0.92)	0.74 (0.92)	0.74 (0.92)	0.86 (0.97)	0.85 (0.96)	0.85 (0.96)	0.83 (0.98)	0.8 (0.95)	0.83 (0.97)	0.96 (1.01)	0.95 (1)	0.97 (1.03)
	$\beta = 0.2$	No	0.73 (0.93)	0.73 (0.92)	0.74 (0.92)	0.8 (0.98)	0.77 (0.94)	0.79 (0.95)	0.88 (1.03)	0.82 (0.97)	0.85 (0.99)	0.99 (1.04)	0.97 (1.02)	1.02 (1.09)
		Yes	0.75 (0.93)	0.75 (0.93)	0.74 (0.92)	0.88 (0.99)	0.86 (0.97)	0.85 (0.97)	0.88 (1.03)	0.82 (0.97)	0.85 (0.99)	0.99 (1.04)	0.97 (1.02)	1.02 (1.09)
sensitivity=100%, specificity=100%	$\beta = 0.15$	No	0.73 (0.92)	0.71 (0.91)	0.72 (0.91)	0.79 (0.96)	0.75 (0.92)	0.75 (0.93)	0.85 (1)	0.79 (0.94)	0.81 (0.96)	0.98 (1.03)	0.95 (1)	0.98 (1.05)
		Yes	0.75 (0.93)	0.74 (0.91)	0.73 (0.91)	0.87 (0.98)	0.85 (0.95)	0.84 (0.96)	0.85 (1)	0.79 (0.94)	0.81 (0.96)	0.98 (1.03)	0.95 (1)	0.98 (1.05)
	$\beta = 0.1$	No	0.72 (0.92)	0.71 (0.91)	0.71 (0.91)	0.77 (0.95)	0.74 (0.92)	0.75 (0.93)	0.83 (0.98)	0.79 (0.94)	0.8 (0.95)	0.96 (1.01)	0.94 (0.99)	0.96 (1.02)
		Yes	0.75 (0.92)	0.73 (0.91)	0.73 (0.91)	0.86 (0.97)	0.84 (0.95)	0.84 (0.95)	0.83 (0.98)	0.79 (0.94)	0.8 (0.95)	0.96 (1.01)	0.94 (0.99)	0.96 (1.02)
	$\beta = 0.2$	No	0.73 (0.93)	0.71 (0.91)	0.72 (0.91)	0.8 (0.98)	0.75 (0.92)	0.76 (0.94)	0.88 (1.03)	0.79 (0.94)	0.81 (0.97)	0.99 (1.04)	0.96 (1.01)	1 (1.06)
		Yes	0.75 (0.93)	0.74 (0.91)	0.73 (0.92)	0.88 (0.99)	0.85 (0.95)	0.84 (0.96)	0.88 (1.03)	0.79 (0.94)	0.81 (0.97)	0.99 (1.04)	0.96 (1.01)	1 (1.06)
sensitivity=80%, specificity=80%	$\beta = 0.15$	No	0.73 (0.92)	0.73 (0.92)	0.78 (0.93)	0.79 (0.96)	0.77 (0.95)	0.82 (0.97)	0.85 (1)	0.83 (0.98)	0.88 (1.01)	0.98 (1.03)	0.97 (1.02)	1 (1.07)
		Yes	0.75 (0.93)	0.75 (0.93)	0.78 (0.93)	0.87 (0.98)	0.86 (0.97)	0.86 (0.97)	0.85 (1)	0.83 (0.98)	0.88 (1.01)	0.98 (1.03)	0.97 (1.02)	1 (1.07)
	$\beta = 0.1$	No	0.72 (0.92)	0.72 (0.91)	0.78 (0.93)	0.77 (0.95)	0.76 (0.94)	0.81 (0.96)	0.83 (0.98)	0.81 (0.96)	0.86 (0.99)	0.96 (1.01)	0.95 (1)	0.97 (1.04)
		Yes	0.75 (0.92)	0.74 (0.92)	0.78 (0.93)	0.86 (0.97)	0.86 (0.96)	0.86 (0.97)	0.83 (0.98)	0.81 (0.96)	0.86 (0.99)	0.96 (1.01)	0.95 (1)	0.97 (1.04)
	$\beta = 0.2$	No	0.73 (0.93)	0.73 (0.92)	0.78 (0.94)	0.8 (0.98)	0.78 (0.96)	0.83 (0.97)	0.88 (1.03)	0.84 (0.99)	0.89 (1.02)	0.99 (1.04)	0.98 (1.03)	1.03 (1.09)
		Yes	0.75 (0.93)	0.75 (0.93)	0.78 (0.94)	0.88 (0.99)	0.87 (0.98)	0.87 (0.98)	0.88 (1.03)	0.84 (0.99)	0.89 (1.02)	0.99 (1.04)	0.98 (1.03)	1.03 (1.09)

Table SI.4: CFR among patients seeking care at four stages of the epidemic according to testing strategy used. We present estimates of CFR and relative decrease compare to the CFR had they not sought care. Outcomes are measured assuming various rate of transmission within hospital, RDT sensitivity and specificity. Outcomes are also presented assuming a decrease in patients' CFR (for those sent to the confirmed ward) by a 0.7 (and 0.9, in bracket) factor.

2.3 Transmission perspective

An important role of health-care units in Ebola settings is to control the epidemic by isolating patients with Ebola, thereby reducing the risk of onward transmission. Therefore we take the natural approach of calculating the reproduction number of infectious individuals seeking care, i.e. the number of new people they infect if they seek care in a health-care unit (R_{HU}). This outcome can be compared with the reproduction number in the community (R). If $R_{HU} < R$, the health-care unit is effectively reducing the population reproduction number; the reduction achieved might differ between diagnostic strategies. We therefore evaluate the testing strategies by their ability to decrease the health-care unit reproduction number relative to the community reproduction number. The reproduction number R_{HU} accounts for: (i) infections before hospitalisation during the delay from symptom onset to hospitalisation, (ii) nosocomial transmission occurring within the triage or confirmed wards, (iii) post-hospitalisation infections, occurring because Ebola-infected patients may be inadvertently discharged to the community due to imperfect sensitivity in the RDT-only strategy, and (iv) community infection from infected patients who presented for care but were turned away due to bed shortage during their infectious period.

2.3.1 Reproduction number using the PCR-only strategy

As using PCR-only is equivalent to using a dual strategy assuming 50% sensitivity and specificity for the RDT test, the contribution of nosocomial infection to the reproduction number is easily calculated as:

$$\left.\frac{\eta_2\left(E^*_{\text{low}}+E^*_{\text{high}}\right)}{\eta_2\left(I^*_{\text{low}}+I^*_{\text{high}}\right)}\right|_{\text{spec}=0.5,\,\text{sens}=0.5} = \frac{\beta\,(1-p)}{\eta_1+\beta p}\left[1+\frac{\eta_1}{\eta_2+\beta p}\right]. \tag{44}$$

In the next generation we must account for infections in the community prior to hospitalisation and nosocomial infections in the holding wards, while due to the perfect sensitivity and specificity of the PCR there will be no further infections in the confirmed ward.

Therefore, the R_{PCR} (for the PCR-only strategy) is:

$$R_{\text{PCR}} = \delta_0 R + \frac{\beta\,(1-p)}{\eta_1+\beta p}\left[1+\frac{\eta_1}{\eta_2+\beta p}\right]. \tag{45}$$

2.3.2 Reproduction number using the dual testing strategy

For the infected patients going through the high-risk ward, in the next generation we must account for infections in the community prior to hospitalisation and nosocomial infections in the initial holding ward and in the high-risk ward.

Therefore, their contribution is:

$$\begin{aligned}&\delta_0 R + \tfrac{E^*_h}{I^*_h} + \tfrac{\left(\eta_2 E^*_{\text{high}} - (1-\text{spec})\eta_1 E^*_h\right)}{\eta_2 I^*_{\text{high}}} = \\ &\qquad \delta_0 R + \tfrac{\beta(1-p)}{\eta_1+\beta p} + \tfrac{\beta(1-p)}{\text{sens}(\eta_1+\beta p)}\left[(1-\text{spec})\left(\tfrac{\eta_1\,\text{sens}}{\eta_2((1-p)(1-\text{spec})+p\,\text{sens})+\beta p\,\text{sens}}\right)\right]\end{aligned}. \tag{46}$$

For the infected patients going through the low-risk ward (due to imperfect RDT sensitivity), in the next generation we must account for infections in the community prior to hospitalisation and nosocomial infections in the initial holding ward and in the low risk ward. Therefore their contribution is:

$$\begin{aligned}&\delta_0 R + \tfrac{E^*_h}{I^*_h} + \tfrac{(\eta_2 E^*_{\text{low}} - (\text{spec})\eta_1 E^*_h)}{\eta_2 I^*_{\text{low}}} = \\ &\qquad \delta_0 R + \tfrac{\beta(1-p)}{\eta_1+\beta p} + \tfrac{\beta(1-p)}{(1-\text{sens})(\eta_1+\beta p)}\left[\text{spec}\left(\tfrac{\eta_1(1-\text{sens})}{\eta_2((1-p)\text{spec}+p\,(1-\text{sens}))+\beta p\,(1-\text{sens})}\right)\right]\end{aligned}. \tag{47}$$

Weighting those by their respective occurrence (i.e. (sens) and $(1-\text{sens})$ respectively), we obtain the R_{Dual} for dual test strategy as:

$$R_{\text{Dual}} = \delta_0 R + \frac{\beta\,(1-p)}{(\eta_1+\beta p)}\left[\begin{array}{c}\left(1+\tfrac{\eta_1(1-\text{sens})\,\text{spec}}{\eta_2((1-p)\text{spec}+p\,(1-\text{sens}))+\beta p\,(1-\text{sens})}\right)+\\ \left(\tfrac{\eta_1\,\text{sens}(1-\text{spec})}{\eta_2((1-p)(1-\text{spec})+p\,\text{sens})+\beta p\,\text{sens}}\right)\end{array}\right]. \tag{48}$$

2.3.3 Reproduction number using the RDT-only strategy

Considering the infected patients who are sent to the confirmed ward, in the next generation we must account for infections in the community prior to hospitalisation and nosocomial infections in the initial holding ward and the confirmed ward. In the confirmed ward, again we assume $p' = \text{sens}\, p/(\text{sens}\, p + (1-\text{spec})\,(1-p))$ are infected and infectious patients, therefore among those patients without Ebola entering the confirmed ward (i.e. $\eta_1\,(1-\text{spec})\,S_h^*$), a proportion $1-\exp\left(-\beta p'\tau_3\right)$ will become infected.

Therefore, the contribution of infected patients who are sent to the confirmed ward is:

$$\begin{aligned} \delta_0 R + \tfrac{E_h^*}{I_h^*} + \tfrac{(1-\text{spec})S_h^*\left[1-\exp\left(-\beta p'\tau_3\right)\right]}{\text{sens}\,I_h^*} = \\ \delta_0 R + \tfrac{\beta(1-p)}{\eta_1+\beta p} + \tfrac{(1-\text{spec})\eta_1(1-p)\left[1-\exp\left(-\beta p'\tau_3\right)\right]}{\text{sens}\,p(\eta_1+\beta p)} \end{aligned} . \quad (49)$$

Alternatively, some infected individuals are discharged back to the community (due to imperfect RDT sensitivity), and in the next generation we must account for infections in the community prior to hospitalisation, nosocomial infections in the initial holding ward and infection back in the community for the remainder of their infectiousness (we represent this period by δ_{RDT}). Therefore their contribution is:

$$\delta_0 R + \frac{E_h^*}{I_h^*} + \delta_{\text{RDT}} R = \delta_0 R + \frac{\beta\,(1-p)}{\eta_1+\beta p} + \delta_{\text{RDT}} R. \quad (50)$$

Again, weighting those by their respective occurrence (i.e. (sens) and $(1-\text{sens})$ respectively), the R_{RDT} for the RDT-only strategy is:

$$R_{\text{RDT}} = \delta_0 R + \frac{\beta\,(1-p)}{\eta_1+\beta p} + \frac{(1-\text{spec})\,\eta_1\,(1-p)\left[1-\exp\left(-\beta p'\tau_3\right)\right]}{p\,(\eta_1+\beta p)} + (1-\text{sens})\,\delta_{\text{RDT}} R. \quad (51)$$

2.3.4 Accounting for bed capacity

Bed capacity is accounted for as before: once it is reached, additional patients are unable to be admitted and are sent back to the community. In the population perspective, it means that the R_{HU}^{admitted} for infected patients admitted is as above, while the $R_{HU}^{\text{not admitted}}$ for infected patients not admitted is the 'community' reproduction number R.

For simplicity, we report the change in reproduction number among infected patients seeking care relative to PCR testing (i.e. $r_{\text{Dual}} = \frac{R_{\text{Dual}}}{R_{\text{PCR}}} - 1$ and $r_{\text{RDT}} = \frac{R_{\text{RDT}}}{R_{\text{PCR}}} - 1$).

2.3.5 Model output

When bed capacity is not limiting, the dual strategy is always preferred. However, if RDT sensitivity and specificity are high, RDT-only may be preferred over PCR-only. When the majority of patients seeking care are infected, RDT sensitivity is key to reduce the discharge of false negatives, while when the majority of patients are not infected with Ebola, RDT specificity is key to reduce the number of false positives (patients without Ebola sent to the confirmed ward).

When bed capacity is limiting, the fast nature of RDT allows more patients to be admitted, dramatically reducing the number of infected patients not being admitted. Therefore RDT-only may become preferred over PCR-only and dual testing.

Figures SI.2 to SI.5 outline the results during four stages of the epidemic (equivalent of Figure 4 in the main text). Note that in these figures the value of R_{HU} for the PCR-only strategy is obtained in the dual strategy if RDT sensitivity and specificity are both set to 50%, meaning a random outcome of the RDT which results in equal prevalence of infection in the low- and high-risk wards. The R_{HU} of the PCR-only strategy is of course independent of the RDT performance.

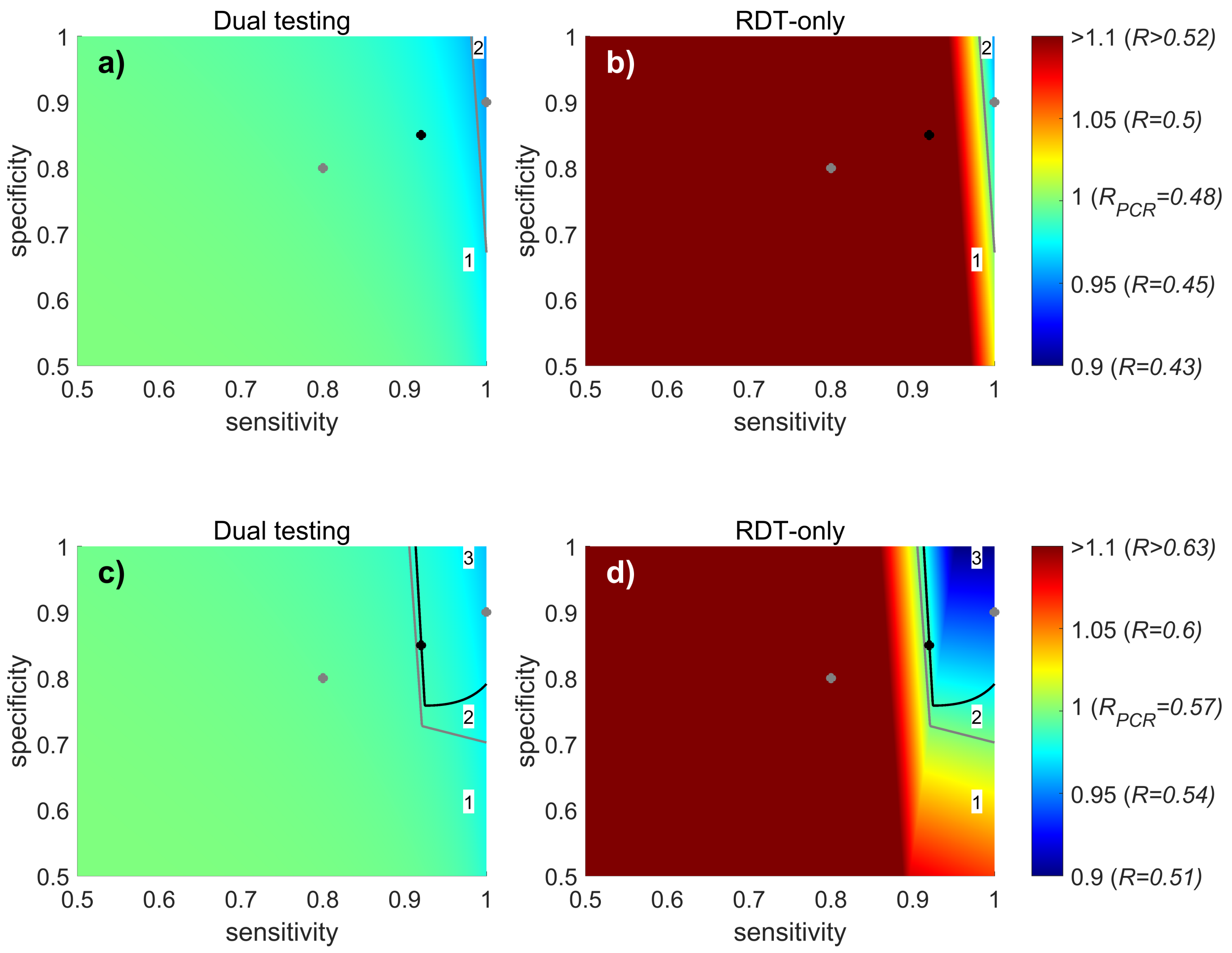

Figure SI.2: Outcomes early during the epidemic in terms of the reproduction number of patients infected with Ebola seeking care for the dual and RDT-only strategies, relative to the PCR-only strategy. For (a & b) bed capacity is unlimited, while for (c & d) the health-care unit has a limit of 60 beds. (a & c) for the dual (RDT+PCR) strategy, (b & d) for the RDT-only strategy. The outcome in the PCR-only strategy is independent of the RDT's sensitivity and specificity. Solid grey and black lines indicate respectively where the outcomes of PCR-only and RDT-only are equivalent, and where the outcomes of dual (RDT+PCR) testing and RDT-only are equivalent. Those lines delimit parameter space where [1] the dual strategy is best followed by the PCR-only and then the RDT-only strategies, [2] the dual strategy is best followed by the RDT-only and then the PCR-only strategies, [3] the RDT-only strategy is best followed by the dual and then the PCR-only strategies. The black circle indicates ReEBOV's reported sensitivity and specificity, while grey dots indicate other potential estimates highlighting the uncertainty in these parameters. The results assume that $\nu = 5$ patients seek care daily with $p = 90\%$ of them infected, health-care unit capacity is 30 beds, the reproduction number for those not seeking care is 1.7 (community reproduction number), on average results for PCR and RDT arrive in 2 days and 1 hour respectively, and the rate of nosocomial transmission is $\beta = 0.15$ per day.

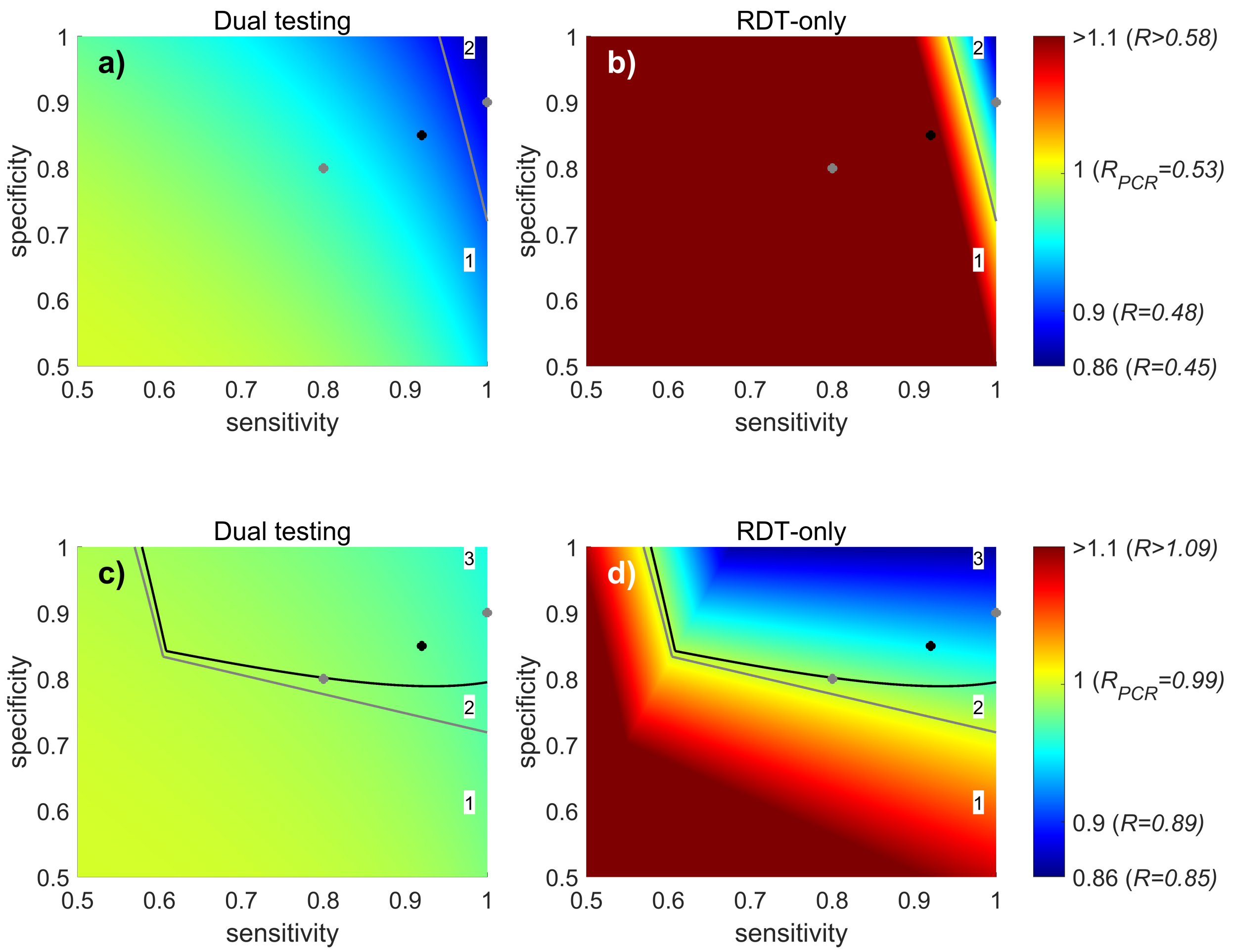

Figure SI.3: Same as Figure SI.2 during the peak of the epidemic (equivalent to Figure 4 in the main text). We assume that $\nu = 60$ patients seek care daily with $p = 70\%$ of them infected, health-care unit capacity is 200 beds, the reproduction number for those not seeking care is 1.7 (community reproduction number) and the rate of nosocomial transmission is $\beta = 0.15$ per day..

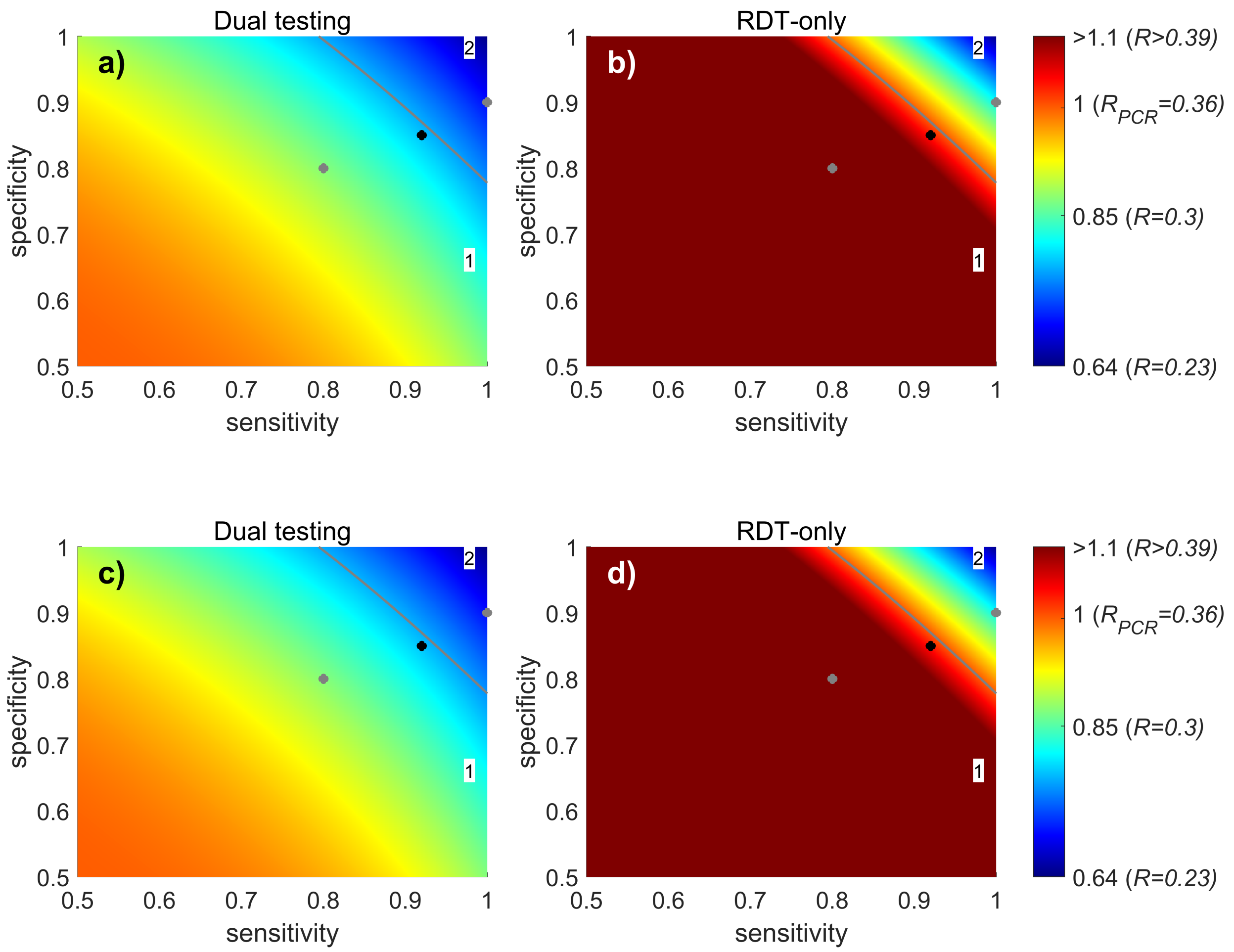

Figure SI.4: Same as Figure SI.2 when the epidemic is decreasing. We assume that $\nu = 60$ patients seek care daily with $p = 50\%$ of them infected, health-care unit capacity is 350 beds, the reproduction number for those not seeking care is 0.85 (community reproduction number) and the rate of nosocomial transmission is $\beta = 0.15$ per day.

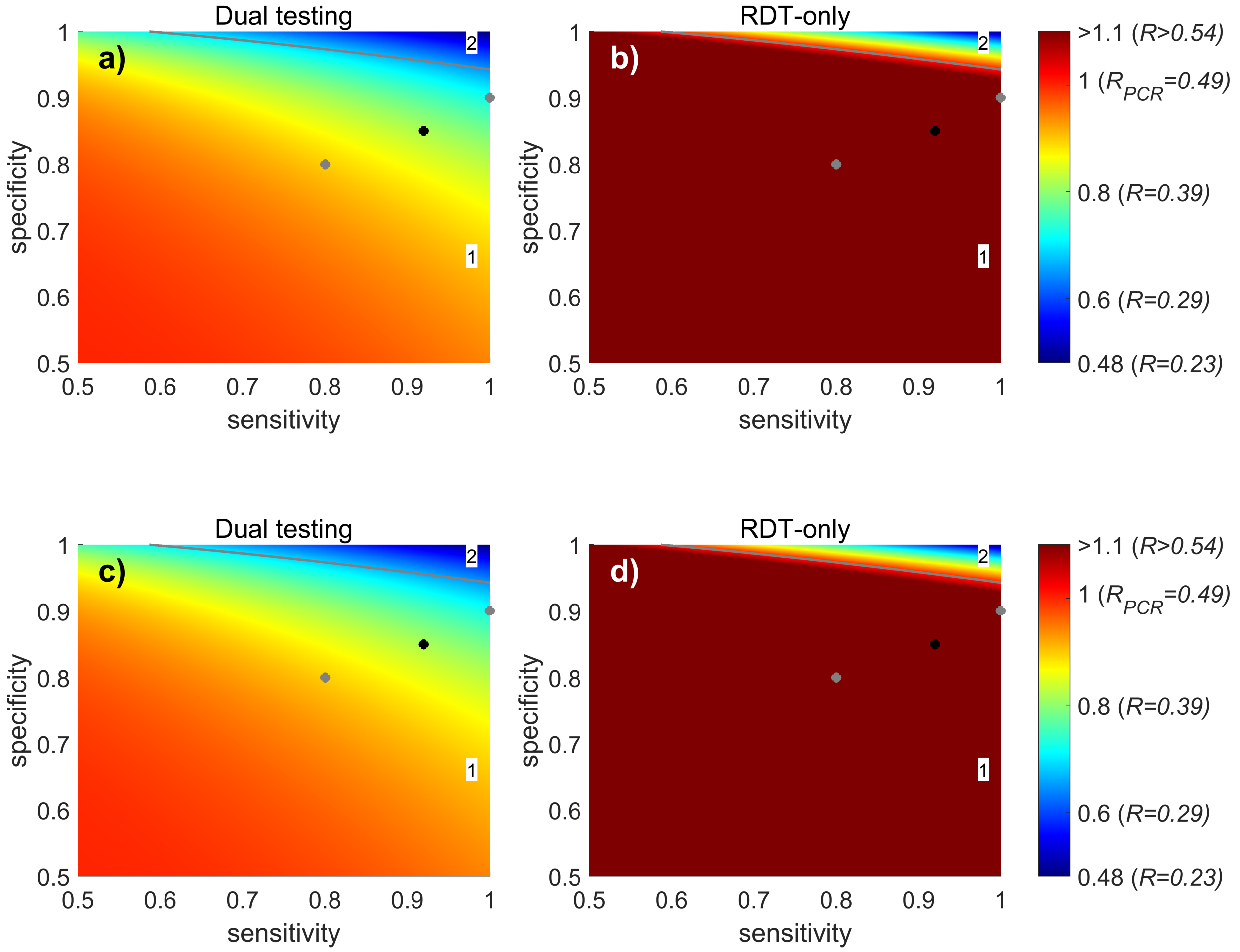

Figure SI.5: Same as Figure SI.2 when the epidemic is tailing-off. We assume that $\nu = 60$ patients seek care daily with $p = 10\%$ of them infected, health-care unit capacity is 350 beds, the reproduction number for those not seeking care is 0.85 (community reproduction number) and the rate of nosocomial transmission is $\beta = 0.15$ per day.

Stage	Bed limits	Testing strategy	ν	p	Community R	Bed capacity	R_{HU} - among patients seeking care	R_{HU} (relative to PCR)	Break down of contributions to the reproduction number: Pre-hospital	Nosocomial (discharged to community)	Nosocomial (send to confirmed)	Wrongly discharged infected	Infected who are turned away	Bed demand	Daily number of patients admitted
Early	No	PCR	5	0.9	1.7	-	0.48	1	0.45	0.02	-	-	-	33	5
		Dual					0.47	0.98	0.45	0.02	-	-	-	33	5
		RDT					0.56	1.18	0.45	0.00	0.01	0.10	-	30	5
	Yes	PCR	5	0.9	1.7	30	0.57	1	0.42	0.02	-	-	0.13	33	5
		Dual					0.56	0.99	0.42	0.01	-	-	0.13	33	5
		RDT					0.56	0.99	0.45	0.00	0.01	0.10	0.00	30	5
Peak	No	PCR	60	0.7	1.7	-	0.53	1	0.45	0.07	-	-	-	330	60
		Dual					0.49	0.92	0.45	0.03	-	-	-	330	60
		RDT					0.59	1.13	0.45	0.00	0.04	0.10	-	290	60
	Yes	PCR	60	0.7	1.7	200	0.99	1	0.27	0.05	-	-	0.67	330	36
		Dual					0.96	0.98	0.27	0.02	-	-	0.67	330	36
		RDT					0.94	0.95	0.31	0.00	0.03	0.07	0.53	290	41
Decreasing	No	PCR	60	0.5	0.85	-	0.36	1	0.23	0.13	-	-	-	270	60
		Dual					0.28	0.79	0.23	0.05	-	-	-	270	60
		RDT					0.37	1.03	0.23	0.00	0.09	0.05	-	226	60
	Yes	PCR	60	0.5	0.85	350	0.36	1	0.23	0.13	-	-	0.00	270	60
		Dual					0.28	0.79	0.23	0.05	-	-	0.00	270	60
		RDT					0.37	1.03	0.23	0.00	0.09	0.05	0.00	226	60
Tail	No	PCR	60	0.1	0.85	-	0.49	1	0.23	0.26	-	-	-	150	60
		Dual					0.40	0.82	0.23	0.17	-	-	-	150	60
		RDT					0.75	1.53	0.23	0.00	0.47	0.05	-	97	60
	Yes	PCR	60	0.1	0.85	350	0.49	1	0.23	0.26	-	-	0.00	150	60
		Dual					0.40	0.82	0.23	0.17	-	-	0.00	150	60
		RDT					0.75	1.53	0.23	0.00	0.47	0.05	0.00	97	60

Table SI.5: Reproduction at four stages of the epidemic according to testing strategy used. We present estimates of absolute reproduction number and relative to that using PCR testing, as well as individual contributions among various classes of patients. '-' indicates not-applicable due to model structure. We assume a rate of hospital infection of $\beta = 0.15$ per day (as in the main text).

Stage	Bed limits	Testing strategy	ν	p	Community R	Bed capacity	$\beta = 0.15$		$\beta = 0.1$		$\beta = 0.2$	
							R_{HU} - among patients seeking care	R_{HU} (relative to PCR)	R_{HU} - among patients seeking care	R_{HU} (relative to PCR)	R_{HU} - among patients seeking care	R_{HU} (relative to PCR)
Early	No	PCR					0.48	1	0.47	1	0.48	1
		Dual	5	0.9	1.7	-	0.47	0.98	0.46	0.99	0.47	0.98
		RDT					0.56	1.18	0.56	1.19	0.57	1.17
	Yes	PCR					0.57	1	0.56	1.00	0.58	1
		Dual	5	0.9	1.7	30	0.56	0.99	0.56	0.99	0.57	0.98
		RDT					0.56	0.99	0.56	0.99	0.57	0.98
Peak	No	PCR					0.53	1	0.51	1	0.55	1
		Dual	60	0.7	1.7	-	0.49	0.92	0.48	0.94	0.50	0.91
		RDT					0.59	1.13	0.58	1.16	0.60	1.10
	Yes	PCR					0.99	1	0.98	1	1.00	1
		Dual	60	0.7	1.7	200	0.96	0.98	0.96	0.98	0.97	0.97
		RDT					0.94	0.95	0.93	0.95	0.94	0.94
Decreasing	No	PCR					0.36	1	0.32	1	0.39	1
		Dual	60	0.5	0.85	-	0.28	0.79	0.26	0.83	0.30	0.75
		RDT					0.37	1.03	0.35	1.09	0.39	0.98
	Yes	PCR					0.36	1	0.32	1	0.39	1
		Dual	60	0.5	0.85	350	0.28	0.79	0.26	0.83	0.30	0.75
		RDT					0.37	1.03	0.35	1.09	0.39	0.98
Tail	No	PCR					0.49	1	0.40	1	0.57	1
		Dual	60	0.1	0.85	-	0.40	0.82	0.35	0.86	0.45	0.78
		RDT					0.75	1.53	0.61	1.52	0.87	1.51
	Yes	PCR					0.49	1	0.40	1	0.57	1
		Dual	60	0.1	0.85	350	0.40	0.82	0.35	0.86	0.45	0.78
		RDT					0.75	1.53	0.61	1.52	0.87	1.51

Table SI.6: Reproduction number and change relative to that using PCR testing at four stages of the epidemic according to testing strategy used. We present estimates assuming a rate of hospital infection of $\beta = 0.1$, $\beta = 0.15$, $\beta = 0.2$ per day.

2.4 Tools to obtain outcomes: parameter values and important caveats

Given the large temporal and spatial heterogeneities that would typically exist between health-care units, we provide tools to calculate the outcomes described above that could be used to assist in determining the optimal strategy that a specific health-care unit should use.

The assumed parameter values determine which strategy is preferred and must therefore be chosen carefully. Ideally a range of parameters should be tested to ensure the robustness of the results to the uncertainties in parameter values (and the tools can be used efficiently for that purpose).

The results given in the main text assume likely parameter values at the peak of the epidemic (see Table 2 in the main text), and are the default settings provided in the tools. An important parameter that is difficult to monitor is the rate of nosocomial transmission, β. A rough (and hopefully an upper bound) estimate may be obtained from the basic reproduction number R_0 and the average duration of infectiousness d_I, assuming $\beta = R_0/d_I$ ($\sim$ 0.15 per day for the Sierra Leone epidemic). We examined a lower and higher rate of nosocomial transmission (at 0.1 and 0.2 per day, see Table SI.6). The level of infection control within the health-care unit would determine whether β is higher or lower than this estimate. Therefore, some level of qualitative judgment must be made as to whether the local rate of nosocomial transmission is greater or lower than that in the community. Experience of nosocomial transmission in past outbreaks would suggest this value should not be reduced too much (i.e. should remain conservative) as health-care units typically will host a large number of infectious patients in confined spaces.

When interpreting the output of the tools, it is important to remember the following limitations.

- The model does not assess the onward transmission from infected patients who are discharged. For instance, they might have a disproportionally large impact early in the epidemic (see population model in the main text).
- In the patient perspective, the model outcomes rely on CFR only. However, patients are likely to base their decision on various factors including, but not limited to, their likely CFR. For instance, safeguarding their relatives from infections would likely be part of their 'perspective'.
- In the patient perspective, the model assumes every patient without Ebola arrives with the same condition. While the characteristics of their conditions (CFR and delay to final outcome) may be reset using the tool, heterogeneities among patients are not considered.
- The model does not track disease progression while waiting for test results within health-care units. However, some patients initially uninfected with Ebola may progress to the infectious stage (I) while waiting for test results, and additionally, some initially infected patients may die or recover during their wait for test results. Sensitivity analyses showed the two progressions, i.e. from exposed to infectious and infectious to death or recovery, balanced well and therefore the health-care-unit-level model assumptions (as used) are robust.
- The model does not account for imperfect sensitivity/specificity of PCR-based tests which may occur in field-conditions. Therefore the results should be interpreted as relative to PCR sensitivity/specificity.
- The model assumes a total 7 days stay in the health-care unit for all patients confirmed with Ebola. If the patients not infected with Ebola were to stay longer or shorter, then the bed occupancy would be affected under the RTD-only testing strategy. This could have an impact especially when most patients seeking care are not infected with Ebola (i.e. in the tail of the outbreak).

3 Population level model

We developed a compartmental population level transmission model to reproduce the dynamics of the ongoing epidemic in West Africa [28, 29] and used this model to assess the impact of different testing and hospitalisation strategies.

Model development proceeded in two stages, (i) describing the natural history of disease and matching this to the observed epidemic, and (ii) adding a meta-population structure to this model to incorporate hospitalisation and testing procedures.

3.1 Model structure

3.1.1 Transmission model

We adopt an SEIR-type model of Ebola transmission in the community:

$$\dot{S} = -\lambda S \tag{52}$$

$$\dot{E}_1 = \lambda S - \alpha_1 E_1 \tag{53}$$

$$\dot{E}_2 = \alpha_1 E_1 - \alpha_2 E_2 \tag{54}$$

$$\dot{I}_1 = \alpha_2 E_2 - \gamma_1 I_1 \tag{55}$$

$$\dot{I}_2 = \gamma_1 I_1 - \gamma_2 I_2 \tag{56}$$

$$\dot{I}_3 = (1-\pi)\gamma_2 I_2 - \gamma_3 I_3 \tag{57}$$

$$\dot{I}_4 = \gamma_3 I_3 - \gamma_4 I_4 \tag{58}$$

$$\dot{aD} = \pi\gamma_2 I_2 - \gamma_D aD \tag{59}$$

$$\dot{dD} = \gamma_D aD \tag{60}$$

$$\dot{dR} = \gamma_4 I_4 \tag{61}$$

Here, the usual conventions for naming the state variables apply: S is the number of susceptibles, E_1 and E_2 the exposed stages, I_1 to I_4 the infectious stages where those that will die move out of I_2 into the highly infectious pre-death stage aD before dying and moving into D, while survivors move through I_3 and I_4 into the recovered class R. Hospitalisation, once included in the model, will happen upon moving from the first infectious stage I_1 to I_2. The α_i are the rates of leaving the exposed stages E_i, $i = 1, 2$, while the γ_i are the rates of leaving the infectious stages I_i, $i = 1 \ldots 4, D$. The case fatality ratio, π, is defined as the proportion of patients with Ebola that die.

The force of infection is given by

$$\lambda = \beta \left(I_1 + I_2 + I_3 + I_4 + \beta_D aD\right) ,$$

where β is the overall transmission parameter, while β_D determines the transmissibility of the acute death stage relative to the other infectious stages, accounting for the very high viral load in extremely sick patients as well as transmission associated with the handling of corpses and funeral practices. The model is calibrated to yield a specified value of R_0 by setting $\beta = R_0/d_{\text{inf.eff}}$, where $d_{\text{inf.eff}}$ is the effective duration of infectiousness, given by

$$d_{\text{inf.eff}} = d_{I_1} + d_{I_2} + (1-\pi)\left(d_{I_3} + d_{I_4}\right) + \pi\beta_D d_{aD} ,$$

using the terminology d_X for the duration of stage X.

The multiple exposed and infectious compartments were used to generate cumulative durations of stay in the exposed and infectious compartments that approximately matched the observed incubation period and onset to death or health-care unit discharge distributions. For instance, the incubation period distribution generated by the model is given by a convolution of the two exponential distributions for the duration of the two exposed stages, E_1 and E_2. If these had the same rates, this would be a gamma distribution with shape parameter 2, for different rates in the two compartments no analytic description can be given, although the distribution can be approximated by a gamma distribution with non-integer shape parameter.

In order to evaluate these distributions, we simulate the clinical course of infection based on the parameter values specified above for 50,000 individuals and compared these empirical distributions to distributions reported in the literature [29].

3.1.2 Model with hospitalisation: both rapid and PCR tests

For taking into account the effect of hospitalisation on the population level transmission as well as evaluating different testing strategies we developed a meta-population model using the basic structure set out in the population level transmission model for each sub-population, with sub-populations for transmission in the community, and in each of four different health-care unit wards: a holding area where patients with suspected Ebola infection are admitted while awaiting lab-results, a low- and a high-risk holding wards and a confirmed ward where patients will be admitted once definitive lab-confirmation via PCR has been received (Figure SI.6). In contrast to a typical meta-population model we do not allow transmission between the different subpopulations; instead infection is spread through the system by people moving between the subpopulations. In the current outbreak, a system of suspected wards/holding centres and confirmed wards has been used widely. As above, we note those: holding wards and confirmed wards. The addition of the low- and high-risk holding wards is a suggested refinement to this system to leverage the benefits of the rapid test despite its lower sensitivity and specificity than the gold standard PCR. We assumed that the death-associated increased transmission risk described by parameter β_D plays a role in the community as well as the holding areas, but that safe handling of the deceased and safe and dignified burial practices are implemented effectively in confirmed wards such that the transmissibility in the acute death stage in these wards is identical to that in any other infectious stage, given by β.

In order to keep track of where people were infected, in each health-care unit ward we separately modelled those who are admitted as susceptible to Ebola and can therefore be infected while in the ward, and subsequently move through the different stages of infection (left hand side of the flow diagrams), and those who were infected with Ebola in the community and enter a health-care unit upon entering the second infectious stage I_2. Movement between health-care unit wards depends on the waiting times for the test results, and upon leaving one ward, people will move into the homologous compartment in the next ward or in the community.

We assumed that a person infected with Ebola will seek care with probability p_{HU}, such that the rate of health-care unit admission is proportional to the Ebola incidence in the community. We further assumed that the incidence of other diseases with compatible symptoms is constant throughout the epidemic, but that these patients' probability of seeking care is proportional to the cumulative number of Ebola deaths D_{tot} that have occurred in the population up to the current time such that the rate of non-Ebola admissions is given by κD_{tot} when health-care unit beds are available, leading to a low number of non-Ebola admissions initially, and saturating at a higher level as the epidemic slows down. As the incidence of both Ebola infection and other diseases with similar symptoms is low at the population level, we did not consider patients sick with another disease but concurrently infected but not yet symptomatic with Ebola. Hence we assume that patients infected with Ebola enter the health-care unit into the I_2 compartment, while those sick with another disease enter the health-care unit as susceptible S.

Discharge from a health-care unit from the holding area or the high or low-risk ward follows a negative PCR or RDT test depending on the testing strategy employed. Importantly, both these tests reflect the infection status upon admission to the holding area rather than the infection status at the time of discharge from the holding area or high or low risk wards. For those patients in the confirmed ward symptomatic with Ebola (I_1 compartment onwards, whether infected in the community or in the health-care unit), upon recovery (entering compartment R), patients need two consecutive negative PCR tests prior to discharge, so the average duration of stay in the R compartments in the confirmed ward is twice the duration of the PCR. Patients who entered the confirmed ward as susceptible or exposed to Ebola, but not yet symptomatic are assumed to take on average τ_{symp} days to recover from the symptoms for which they sought care and then need 2 consecutive negative PCR tests prior to discharge such that the average time spent in the S, E_1 and E_2 compartments of the confirmed ward is $\tau_{\mathrm{symp}} - \tau_2 + \tau_3$, unless they progress into the symptomatic and infectious stages of Ebola disease (compartments I_1 onwards). Here, τ_2 is the average waiting time for the PCR test results upon hospital admission which determines the delay from admission to the holding area to admission to the confirmed ward, and τ_3 is the average waiting time for the hospital discharge tests, typically

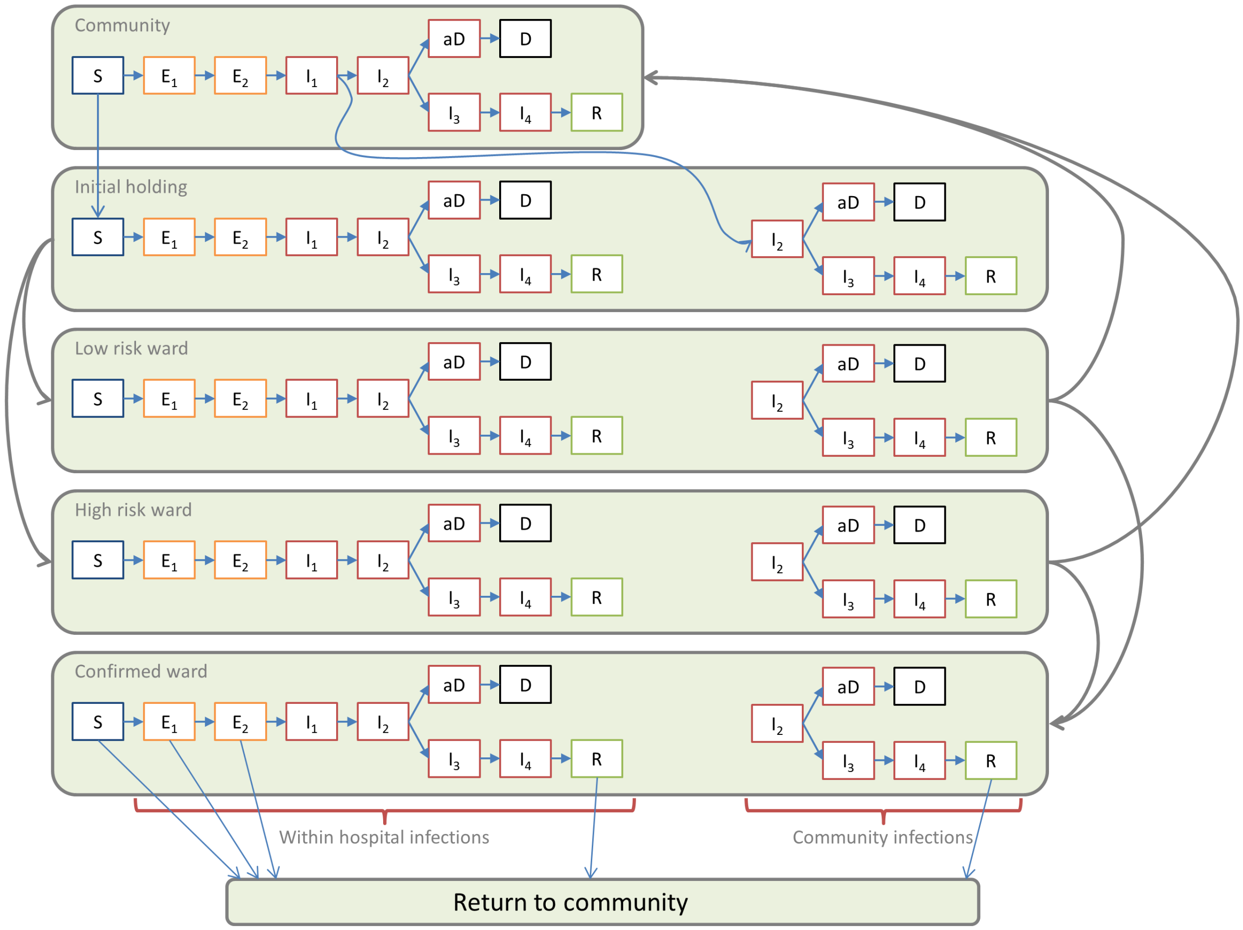

Figure SI.6: Flow diagram of the full transmission model with health-care unit structure.

set to $\tau_3 = 2\tau_2$.

Health-care unit beds are limited, and when bed capacity is reached and the rate of patients seeking care is higher than the rate at which health-care unit beds are vacated through either discharge or death, the effective rate of hospitalisation of patients both with and without Ebola infection is adjusted by a factor $p_{\text{HU.corr}}$ to ensure bed capacity constraints are not breached.

Specifically the model is described by the following equations:

The force of infection in the sub-populations (i.e. wards) is given by:

$$\lambda_{\text{com}} = \beta \left(I_{1,\text{com}} + I_{2,\text{com}} + I_{3,\text{com}} + I_{4,\text{com}} + \beta_{D,\text{com}} aD_{\text{com}} \right) / N_{\text{com}} \tag{62}$$

$$\begin{aligned} \lambda_{\text{hold}} = \beta \big(& I_{1,\text{hold}} + I_{2,\text{hold}} + I_{3,\text{hold}} + I_{4,\text{hold}} + \beta_{D,\text{com}} aD_{\text{hold}} \\ & + I_{2,\text{hold,icom}} + I_{3,\text{hold,icom}} + I_{4,\text{hold,icom}} + \beta_{D,\text{com}} aD_{\text{hold,icom}} \big) / N_{\text{hold}} \end{aligned} \tag{63}$$

$$\begin{aligned} \lambda_{\text{low}} = \beta \big(& I_{1,\text{low}} + I_{2,\text{low}} + I_{3,\text{low}} + I_{4,\text{low}} + \beta_{D,\text{com}} aD_{\text{low}} \\ & + I_{2,\text{low,icom}} + I_{3,\text{low,icom}} + I_{4,\text{low,icom}} + \beta_{D,\text{com}} aD_{\text{low,icom}} \big) / N_{\text{low}} \end{aligned} \tag{64}$$

$$\begin{aligned} \lambda_{\text{high}} = \beta \big(& I_{1,\text{high}} + I_{2,\text{high}} + I_{3,\text{high}} + I_{4,\text{high}} + \beta_{D,\text{com}} aD_{\text{high}} \\ & + I_{2,\text{high,icom}} + I_{3,\text{high,icom}} + I_{4,\text{high,icom}} + \beta_{D,\text{com}} aD_{\text{high,icom}} \big) / N_{\text{high}} \end{aligned} \tag{65}$$

$$\begin{aligned} \lambda_{\text{conf}} = \beta \big(& I_{1,\text{conf}} + I_{2,\text{conf}} + I_{3,\text{conf}} + I_{4,\text{conf}} + \beta_{D,\text{HU}} aD_{\text{conf}} \\ & + I_{2,\text{conf,icom}} + I_{3,\text{conf,icom}} + I_{4,\text{conf,icom}} + \beta_{D,\text{HU}} aD_{\text{conf,icom}} \big) / N_{\text{conf}} \end{aligned} \tag{66}$$

Transmission within the community:

$$\dot{S}_{\text{com}} = -\lambda_{\text{com}} S_{\text{com}} - \kappa D_{\text{tot}} S_{\text{com}} p_{\text{HU.corr}} + \eta_2 \text{spec}_2 \left(S_{\text{low}} + S_{\text{high}} \right) + \eta_4 S_{\text{conf}} \tag{67}$$

$$\dot{E}_{1,\text{com}} = \lambda_{\text{com}} S_{\text{com}} - \alpha_1 E_{1,\text{com}} + \eta_2 \text{spec}_2 \left(E_{1,\text{low}} + E_{1,\text{high}} \right) + \eta_4 E_{1,\text{conf}} \tag{68}$$

$$\dot{E}_{2,\text{com}} = \alpha_1 E_{1,\text{com}} - \alpha_2 E_{2,\text{com}} + \eta_2 \text{spec}_2 \left(E_{2,\text{low}} + E_{2,\text{high}} \right) + \eta_4 E_{2,\text{conf}} \tag{69}$$

$$\dot{I}_{1,\text{com}} = \alpha_2 E_{2,\text{com}} - \gamma_1 I_{1,\text{com}} + \eta_2 \, \text{spec}_2 \left(I_{1,\text{low}} + I_{1,\text{high}} \right) \tag{70}$$

$$\begin{aligned} \dot{I}_{2,\text{com}} = & \left(1 - p_{\text{HU}} p_{\text{HU.corr}} \right) \gamma_1 I_{1,\text{com}} - \gamma_2 I_{2,\text{com}} \\ & + \eta_2 \, \text{spec}_2 \left(I_{2,\text{low}} + I_{2,\text{high}} \right) + \eta_2 \left(1 - \text{sens}_2 \right) \left(I_{2,\text{low,icom}} + I_{2,\text{high,icom}} \right) \end{aligned} \tag{71}$$

$$\begin{aligned} \dot{I}_{3,\text{com}} = & \left(1 - \pi \right) \gamma_2 I2_{\text{com}} - \gamma_3 I_{3,\text{com}} \\ & + \eta_2 \, \text{spec}_2 \left(I_{3,\text{low}} + I_{3,\text{high}} \right) + \eta_2 \left(1 - \text{sens}_2 \right) \left(I_{3,\text{low,icom}} + I_{3,\text{high,icom}} \right) \end{aligned} \tag{72}$$

$$\dot{I}_{4,\text{com}} = \gamma_3 I_{3,\text{com}} - \gamma_4 I_{4,\text{com}} + \eta_2 \, \text{spec}_2 \left(I_{4,\text{low}} + I_{4,\text{high}} \right) + \eta_2 \left(1 - \text{sens}_2 \right) \left(I_{4,\text{low,icom}} + I_{4,\text{high,icom}} \right) \tag{73}$$

$$\begin{aligned} \dot{aD}_{\text{com}} = & \pi \gamma_2 I_{2,\text{com}} - \gamma_D aD_{\text{com}} \\ & + \eta_2 \, \text{spec}_2 \left(aD_{\text{low}} + aD_{\text{high}} \right) + \eta_2 \left(1 - \text{sens}_2 \right) \left(aD_{\text{low,icom}} + aD_{\text{high,icom}} \right) \end{aligned} \tag{74}$$

$$\dot{D}_{\text{com}} = \gamma_D aD_{\text{com}} \tag{75}$$

$$\begin{aligned} \dot{R}_{\text{com}} = & \gamma_4 I_{4,\text{com}} + \eta_2 \, \text{spec}_2 \left(R_{\text{low}} + R_{\text{high}} \right) \\ & + \eta_2 \left(1 - \text{sens}_2 \right) \left(R_{\text{low,icom}} + R_{\text{high,icom}} \right) + \eta_3 \left(R_{\text{conf}} + R_{\text{conf,icom}} \right) \end{aligned} \tag{76}$$

Transmission within the initial holding ward, infection within a health-care unit:

$$\dot{S}_{\text{hold}} = -\lambda_{\text{hold}} S_{\text{hold}} + \kappa D_{\text{tot}} S_{\text{com}} p_{\text{HU.corr}} - \eta_1 S_{\text{hold}} \tag{77}$$
$$\dot{E}_{1,\text{hold}} = \lambda_{\text{hold}} S_{\text{hold}} - (\alpha_1 + \eta_1) E_{1,\text{hold}} \tag{78}$$
$$\dot{E}_{2,\text{hold}} = \alpha_1 E_{1,\text{hold}} - (\alpha_2 + \eta_1) E_{2,\text{sus}} \tag{79}$$
$$\dot{I}_{1,\text{hold}} = \alpha_2 E_{2,\text{hold}} - (\gamma_1 + \eta_1) I_{1,\text{hold}} \tag{80}$$
$$\dot{I}_{2,\text{hold}} = \gamma_1 I_{1,\text{hold}} - (\gamma_2 + \eta_1) I_{2,\text{hold}} \tag{81}$$
$$\dot{I}_{3,\text{hold}} = (1-\pi)\gamma_2 I_{2,\text{hold}} - (\gamma_3 + \eta_1) I_{3,\text{hold}} \tag{82}$$
$$\dot{I}_{4,\text{hold}} = \gamma_3 I_{3,\text{hold}} - (\gamma_4 + \eta_1) I_{4,\text{hold}} \tag{83}$$
$$a\dot{D}_{\text{hold}} = \pi\gamma_2 I_{2,\text{hold}} - (\gamma_D + \eta_1) aD_{\text{hold}} \tag{84}$$
$$\dot{D}_{\text{hold}} = \gamma_D aD_{\text{hold}} \tag{85}$$
$$\dot{R}_{\text{hold}} = \gamma_4 I_{4,\text{hold}} - \eta_1 R_{\text{hold}} \tag{86}$$

Transmission within the initial holding ward, infection within the community:

$$\dot{I}_{2,\text{hold,icom}} = p_{\text{HU}} p_{\text{HU.corr}} \gamma_1 I_{1,\text{com}} - (\gamma_2 + \eta_1) I_{2,\text{hold,icom}} \tag{87}$$
$$\dot{I}_{3,\text{hold,icom}} = (1-\pi)\gamma_2 I_{2,\text{hold,icom}} - (\gamma_3 + \eta_1) I_{3,\text{hold,icom}} \tag{88}$$
$$\dot{I}_{4,\text{hold,icom}} = \gamma_3 I_{3,\text{hold,icom}} - (\gamma_4 + \eta_1) I_{4,\text{hold,icom}} \tag{89}$$
$$a\dot{D}_{\text{hold,icom}} = \pi\gamma_2 I_{2,\text{hold,icom}} - (\gamma_D + \eta_1) aD_{\text{hold,icom}} \tag{90}$$
$$\dot{D}_{\text{hold,icom}} = \gamma_D aD_{\text{hold,icom}} \tag{91}$$
$$\dot{R}_{\text{hold,icom}} = \gamma_4 I_{4,\text{hold,icom}} - \eta_1 R_{\text{hold,icom}} \tag{92}$$

Transmission within the low-risk ward, infection within a health-care unit:

$$\dot{S}_{\text{low}} = -\lambda_{\text{low}} S_{\text{low}} - \eta_2 S_{\text{low}} + \eta_1 \text{spec}_1 S_{\text{hold}} \tag{93}$$
$$\dot{E}_{1,\text{low}} = \lambda_{\text{low}} S_{\text{low}} - (\alpha_1 + \eta_2) E_{1,\text{low}} + \eta_1 \text{spec}_1 E_{1,\text{hold}} \tag{94}$$
$$\dot{E}_{2,\text{low}} = \alpha_1 E_{1,\text{low}} - (\alpha_2 + \eta_2) E_{2,\text{low}} + \eta_1 \text{spec}_1 E_{2,\text{hold}} \tag{95}$$
$$\dot{I}_{1,\text{low}} = \alpha_2 E_{2,\text{low}} - (\gamma_1 + \eta_2) I_{1,\text{low}} + \eta_1 \text{spec}_1 I_{1,\text{hold}} \tag{96}$$
$$\dot{I}_{2,\text{low}} = \gamma_1 I_{1,\text{low}} - (\gamma_2 + \eta_2) I_{2,\text{low}} + \eta_1 \text{spec}_1 I_{2,\text{hold}} \tag{97}$$
$$\dot{I}_{3,\text{low}} = (1-\pi)\gamma_2 I_{2,\text{low}} - (\gamma_3 + \eta_2) I_{3,\text{low}} + \eta_1 \text{spec}_1 I_{3,\text{hold}} \tag{98}$$
$$\dot{I}_{4,\text{low}} = \gamma_3 I_{3,\text{low}} - (\gamma_4 + \eta_2) I_{4,\text{low}} + \eta_1 \text{spec}_1 I_{4,\text{hold}} \tag{99}$$
$$a\dot{D}_{\text{low}} = \pi\gamma_2 I_{2,\text{low}} - (\gamma_D + \eta_2) aD_{\text{low}} + \eta_1 \text{spec}_1 aD_{\text{hold}} \tag{100}$$
$$\dot{D}_{\text{low}} = \gamma_D aD_{\text{low}} \tag{101}$$
$$\dot{R}_{\text{low}} = \gamma_4 I_{4,\text{low}} - \eta_2 R_{\text{low}} + \eta_1 \text{spec}_1 R_{\text{hold}} \tag{102}$$

Transmission within the low-risk ward, infection within the community:

$$\dot{I}_{2,\text{low,icom}} = -(\gamma_2 + \eta_2) I_{2,\text{low,icom}} + \eta_1 (1 - \text{sens}_1) I_{2,\text{hold,icom}} \tag{103}$$
$$\dot{I}_{3,\text{low,icom}} = (1-\pi)\gamma_2 I_{2,\text{low,icom}} - (\gamma_3 + \eta_2) I_{3,\text{low,icom}} + \eta_1 (1 - \text{sens}_1) I_{3,\text{hold,icom}} \tag{104}$$
$$\dot{I}_{4,\text{low,icom}} = \gamma_3 I_{3,\text{low,icom}} - (\gamma_4 + \eta_2) I_{4,\text{low,icom}} + \eta_1 (1 - \text{sens}_1) I_{4,\text{hold,icom}} \tag{105}$$
$$a\dot{D}_{\text{low,icom}} = \pi\gamma_2 I_{2,\text{low,icom}} - (\gamma_D + \eta_2) aD_{\text{low,icom}} + \eta_1 (1 - \text{sens}_1) aD_{\text{hold,icom}} \tag{106}$$
$$\dot{D}_{\text{low,icom}} = \gamma_D aD_{\text{low,icom}} \tag{107}$$
$$\dot{R}_{\text{low,icom}} = \gamma_4 I_{4,\text{low,icom}} - \eta_2 R_{\text{low,icom}} + \eta_1 (1 - \text{sens}_1) R_{\text{hold,icom}} \tag{108}$$

Transmission within the high-risk ward, infection within a health-care unit:

$$\dot{S}_{\text{high}} = -\lambda_{\text{high}} S_{\text{high}} - \eta_2 S_{\text{high}} + \eta_1 \left(1 - \text{spec}_1\right) S_{\text{hold}} \tag{109}$$

$$\dot{E}_{1,\text{high}} = \lambda_{\text{high}} S_{\text{high}} - \left(\alpha_1 + \eta_2\right) E_{1,\text{high}} + \eta_1 \left(1 - \text{spec}_1\right) E_{1,\text{hold}} \tag{110}$$

$$\dot{E}_{2,\text{high}} = \alpha_1 E_{1,\text{high}} - \left(\alpha_2 + \eta_2\right) E_{2,\text{high}} + \eta_1 \left(1 - \text{spec}_1\right) E_{2,\text{hold}} \tag{111}$$

$$\dot{I}_{1,\text{high}} = \alpha_2 E_{2,\text{high}} - \left(\gamma_1 + \eta_2\right) I_{1,\text{high}} + \eta_1 \left(1 - \text{spec}_1\right) I_{1,\text{hold}} \tag{112}$$

$$\dot{I}_{2,\text{high}} = \gamma_1 I_{1,\text{high}} - \left(\gamma_2 + \eta_2\right) I_{2,\text{high}} + \eta_1 \left(1 - \text{spec}_1\right) I_{2,\text{hold}} \tag{113}$$

$$\dot{I}_{3,\text{high}} = \left(1 - \pi\right) \gamma_2 I_{2,\text{high}} - \left(\gamma_3 + \eta_2\right) I_{3,\text{high}} + \eta_1 \left(1 - \text{spec}_1\right) I_{3,\text{hold}} \tag{114}$$

$$\dot{I}_{4,\text{high}} = \gamma_3 I_{3,\text{high}} - \left(\gamma_4 + \eta_2\right) I_{4,\text{high}} + \eta_1 \left(1 - \text{spec}_1\right) I_{4,\text{hold}} \tag{115}$$

$$\dot{aD}_{\text{high}} = \pi \gamma_2 I_{2,\text{high}} - \left(\gamma_D + \eta_2\right) aD_{\text{high}} + \eta_1 \left(1 - \text{spec}_1\right) aD_{\text{hold}} \tag{116}$$

$$\dot{D}_{\text{high}} = \gamma_D aD_{\text{high}} \tag{117}$$

$$\dot{R}_{\text{high}} = \gamma_4 I_{4,\text{high}} - \eta_2 R_{\text{high}} + \eta_1 \left(1 - \text{spec}_1\right) R_{\text{hold}} \tag{118}$$

Transmission within the high-risk ward, infection within the community:

$$\dot{I}_{2,\text{high,icom}} = -\left(\gamma_2 + \eta_2\right) I_{2,\text{high,icom}} + \eta_1 \text{sens}_1 I_{2,\text{hold,icom}} \tag{119}$$

$$\dot{I}_{3,\text{high,icom}} = \left(1 - \pi\right) \gamma_2 I_{2,\text{high,icom}} - \left(\gamma_3 + \eta_2\right) I_{3,\text{high,icom}} + \eta_1 \text{sens}_1 I_{3,\text{hold,icom}} \tag{120}$$

$$\dot{I}_{4,\text{high,icom}} = \gamma_3 I_{3,\text{high,icom}} - \left(\gamma_4 + \eta_2\right) I_{4,\text{high,icom}} + \eta_1 \text{sens}_1 I_{4,\text{hold,icom}} \tag{121}$$

$$\dot{aD}_{\text{high,icom}} = \pi \gamma_2 I_{2,\text{high,icom}} - \left(\gamma_D + \eta_2\right) aD_{\text{high,icom}} + \eta_1 \text{sens}_1 aD_{\text{hold,icom}} \tag{122}$$

$$\dot{D}_{\text{high,icom}} = \gamma_D aD_{\text{high,icom}} \tag{123}$$

$$\dot{R}_{\text{high,icom}} = \gamma_4 I_{4,\text{high,icom}} - \eta_2 R_{\text{high,icom}} + \eta_1 \text{sens}_1 R_{\text{hold,icom}} \tag{124}$$

Transmission within the confirmed ward, infection within a health-care unit:

$$\dot{S}_{\text{conf}} = -\lambda_{\text{conf}} S_{\text{conf}} + \eta_2 \left(1 - \text{spec}_2\right) \left(S_{\text{low}} + S_{\text{high}}\right) - \eta_4 S_{\text{conf}} \tag{125}$$

$$\dot{E}_{1,\text{conf}} = \lambda_{\text{conf}} S_{\text{conf}} - \alpha_1 E_{1,\text{conf}} + \eta_2 \left(1 - \text{spec}_2\right) \left(E_{1,\text{low}} + E_{1,\text{high}}\right) - \eta_4 E_{1,\text{conf}} \tag{126}$$

$$\dot{E}_{2,\text{conf}} = \alpha_1 E_{1,\text{conf}} - \alpha_2 E_{2,\text{conf}} + \eta_2 \left(1 - \text{spec}_2\right) \left(E_{2,\text{low}} + E_{2,\text{high}}\right) - \eta_4 E_{2,\text{conf}} \tag{127}$$

$$\dot{I}_{1,\text{conf}} = \alpha_2 E_{2,\text{conf}} - \gamma_1 I_{1,\text{conf}} + \eta_2 \left(1 - \text{spec}_2\right) \left(I_{1,\text{low}} + I_{1,\text{high}}\right) \tag{128}$$

$$\dot{I}_{2,\text{conf}} = \gamma_1 I_{1,\text{conf}} - \gamma_2 I_{2,\text{conf}} + \eta_2 \left(1 - \text{spec}_2\right) \left(I_{2,\text{low}} + I_{2,\text{high}}\right) \tag{129}$$

$$\dot{I}_{3,\text{conf}} = \left(1 - \pi\right) \gamma_2 I_{2,\text{conf}} - \gamma_3 I_{3,\text{conf}} + \eta_2 \left(1 - \text{spec}_2\right) \left(I_{3,\text{low}} + I_{3,\text{high}}\right) \tag{130}$$

$$\dot{I}_{4,\text{conf}} = \gamma_3 I_{3,\text{conf}} - \gamma_4 I_{4,\text{conf}} + \eta_2 \left(1 - \text{spec}_2\right) \left(I_{4,\text{low}} + I_{4,\text{high}}\right) \tag{131}$$

$$\dot{aD}_{\text{conf}} = \pi \gamma_2 I_{2,\text{conf}} - \gamma_D aD_{\text{conf}} + \eta_2 \left(1 - \text{spec}_2\right) \left(aD_{\text{low}} + aD_{\text{high}}\right) \tag{132}$$

$$\dot{D}_{\text{conf}} = \gamma_D aD_{\text{conf}} \tag{133}$$

$$\dot{R}_{\text{conf}} = \gamma_4 I_{4,\text{conf}} + \eta_2 \left(1 - \text{spec}_2\right) \left(R_{\text{low}} + R_{\text{high}}\right) - \eta_3 R_{\text{conf}} \tag{134}$$

Transmission within the confirmed ward, infection within the community:

$$\dot{I}_{2,\text{conf,icom}} = -\gamma_2 I_{2,\text{conf,icom}} + \eta_2 \text{sens}_2 \left(I_{2,\text{low,icom}} + I_{2,\text{high,icom}}\right) \tag{135}$$

$$\dot{I}_{3,\text{conf,icom}} = \left(1 - \pi\right) \gamma_2 I_{2,\text{conf,icom}} - \gamma_3 I_{3,\text{conf,icom}} + \eta_2 \text{sens}_2 \left(I_{3,\text{low,icom}} + I_{3,\text{high,icom}}\right) \tag{136}$$

$$\dot{I}_{4,\text{conf,icom}} = \gamma_3 I_{3,\text{conf,icom}} - \gamma_4 I_{4,\text{conf,icom}} + \eta_2 \text{sens}_2 \left(I_{4,\text{low,icom}} + I_{4,\text{high,icom}}\right) \tag{137}$$

$$\dot{aD}_{\text{conf,icom}} = \pi \gamma_2 I_{2,\text{conf,icom}} - \gamma_D aD_{\text{conf,icom}} + \eta_2 \text{sens}_2 \left(aD_{\text{low,icom}} + aD_{\text{high,icom}}\right) \tag{138}$$

$$\dot{D}_{\text{conf,icom}} = \gamma_D aD_{\text{conf,icom}} \tag{139}$$

$$\dot{R}_{\text{conf,icom}} = \gamma_4 I_{4,\text{conf,icom}} + \eta_2 \text{sens}_2 \left(R_{\text{low,icom}} + R_{\text{high,icom}}\right) - \eta_3 R_{\text{conf,icom}} \tag{140}$$

Here, the same state variables as in the simpler transmission model without hospitalisation are used, but subscripted with 'com', 'hold', 'low', 'high', 'conf' to indicate the subpopulation: the community, the

initial holding area, and the low-risk, high-risk and confirmed wards, respectively. The additional subscript 'icom' is used to denote patients that were infected in the community rather than within the health-care unit environment (right hand side of the flow diagrams in Figure SI.6). The rates of disease progression are the same as in the simpler model without health-care unit structure, while the rates η_i describe the rates of moving between the different wards. Test sensitivity and specificity sens_i and spec_i of the RDT ($i = 1$) and PCR ($i = 2$) determine the proportion of patients correctly referred to the confirmed ward or discharged back to the community.

3.1.3 2-ward model for the PCR-only and RDT-only strategies

The PCR-only and RDT-only strategies are modelled by a 2-ward model lacking the low- and high-risk wards in the full 4-ward model described above (Figure SI.7). The model structure for both is identical even though the parametrisation differs in terms of test duration and accuracy. The PCR-only strategy reflects best the strategy that has been in use throughout the current epidemic.

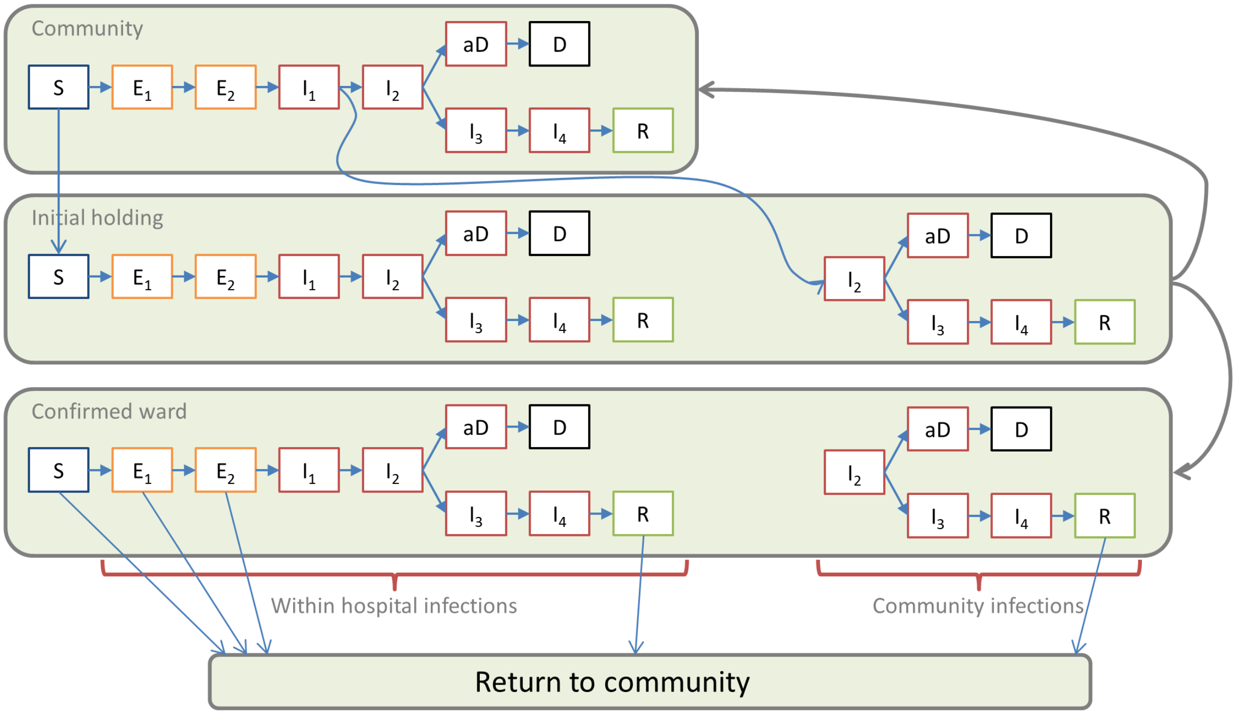

Figure SI.7: Flow diagram of the 2-ward transmission model for the PCR-only and RDT-only strategies.

The two-ward model can be obtained as a special case of the four-ward model by setting the waiting time for the second test τ_{test2} equal to that of the test employed in this strategy, while setting the waiting time for the first test $\tau_{\text{test1}} = \tau_{\text{test2}} - \epsilon$, ϵ small. This ensures that patients remain in the holding area while waiting for their test results and spending only a minimal amount of time transiting through the low- or high-risk ward. Furthermore, the sensitivity and specificity of the first test should be set to $\text{sens}_1 = \text{spec}_1 = 50\%$ to achieve random allocation rather than segregation of patients with and without Ebola into the low- and high-risk wards, and the sensitivity and specificity of the second test which determines whether a patient is discharged back to the community or admitted to the confirmed ward should be set to that of the test employed in this strategy. In the limit of 0 waiting time in the low- and high-risk wards $\epsilon \to 0$ the values sens_1 and spec_1 assumed for the first (in this strategy not performed) test do not actually play a role as there is no time for transmission in the segregated wards.

3.2 Model calibration

Model calibration proceeded in two steps: (i) calibration of the natural history parameters to match the individual level delays observed in the current outbreak, and (ii) calibration of parameters shaping the overall epidemic curve.

3.2.1 Calibration of the basic population level transmission model

For matching the observed delays we used the basic population level transmission model without hospitalisation. Table SI.7 shows the parameters calibrated here and the baseline values chosen, while Figure SI.8 shows how the delay distributions obtained from our model with these parameter values match those observed in the current outbreak [29].

Note that the value of $\beta_{D,\text{com}} = 16$ per day was chosen in order to achieve a similar level of transmission from patients who die and survive, accounting for the shorter duration of infectiousness in patients who die than survivors.

Table SI.7: Natural history parameters of disease progression, their descriptions and baseline values.

Parameter	Description	Relation	Value
d_{E_1}	duration of stage E_1		8.0
d_{E_2}	duration of stage E_2		2.5
d_{I_1}	duration of stage I_2		4.5
d_{I_2}	duration of stage I_2		3.0
d_{I_3}	duration of stage I_3		4.5
d_{I_4}	duration of stage I_4		3.5
d_{aD}	duration of acute death stage		0.5
$d_{I_{\text{tot}}}$	effective total duration of infectious stages	$d_{I_1} + d_{I_2} + (1-\pi)(d_{I_3} + d_{I_4}) + \pi\beta_{D,\text{com}} d_{aD}$	15.5
α_1	rate of leaving stage E_1	$1/d_{E_1}$	0.125
α_2	rate of leaving stage E_2	$1/d_{E_2}$	0.400
γ_1	rate of leaving stage I_2	$1/d_{I_1}$	0.222
γ_2	rate of leaving stage I_2	$1/d_{I_2}$	0.333
γ_3	rate of leaving stage I_3	$1/d_{I_3}$	0.222
γ_4	rate of leaving stage I_4	$1/d_{I_4}$	0.286
γ_D	rate of leaving stage aD	$1/d_{aD}$	2.000
π	case fatality rate		0.6
$\beta_{D,\text{com}}$	relative transmissibility of acute death stage		16
$\beta_{D,\text{HU}}$	relative transmissibility of actue death stage in confirmed ward		1

3.2.2 Calibration of the full transmission model with health-care unit structure

During the current outbreak the PCR-only testing strategy was in use. We therefore calibrate the overall transmission and control parameters to match the modelled incidence to incidence observed in Sierra Leone (patients with confirmed and probable Ebola infection), and evaluate the model calibrated thus for different testing strategies as counter-factual scenarios to assess the impact these strategies could have had on the scale of the epidemic.

Parameters of the test characteristics (duration, sensitivity and specificity) were kept fixed, although we evaluated 2 distinct scenarios with regards to test sensitivity and specificity: In our baseline scenario, we assumed that PCR has 100% sensitivity and specificity, and the RDT has 92% sensitivity of and 85% specificity as measured in the lab [3]. However, as both PCR and RDT test performance in the field is likely lower than in the lab, as a sensitivity analysis we also consider an alternative scenario where we assume 85% and 82% sensitivity and 95% and 80% specificity of the PCR and RDT, respectively. We assumed a more pronounced loss of test accuracy for the PCR than for the RDT due to the larger logistical challenges. We separately calibrated this alternative scenario of realistic test accuracy against the observed incidence. The assumed values for the test characteristics and the subsequent testing rates fed into the model are shown in Table SI.8.

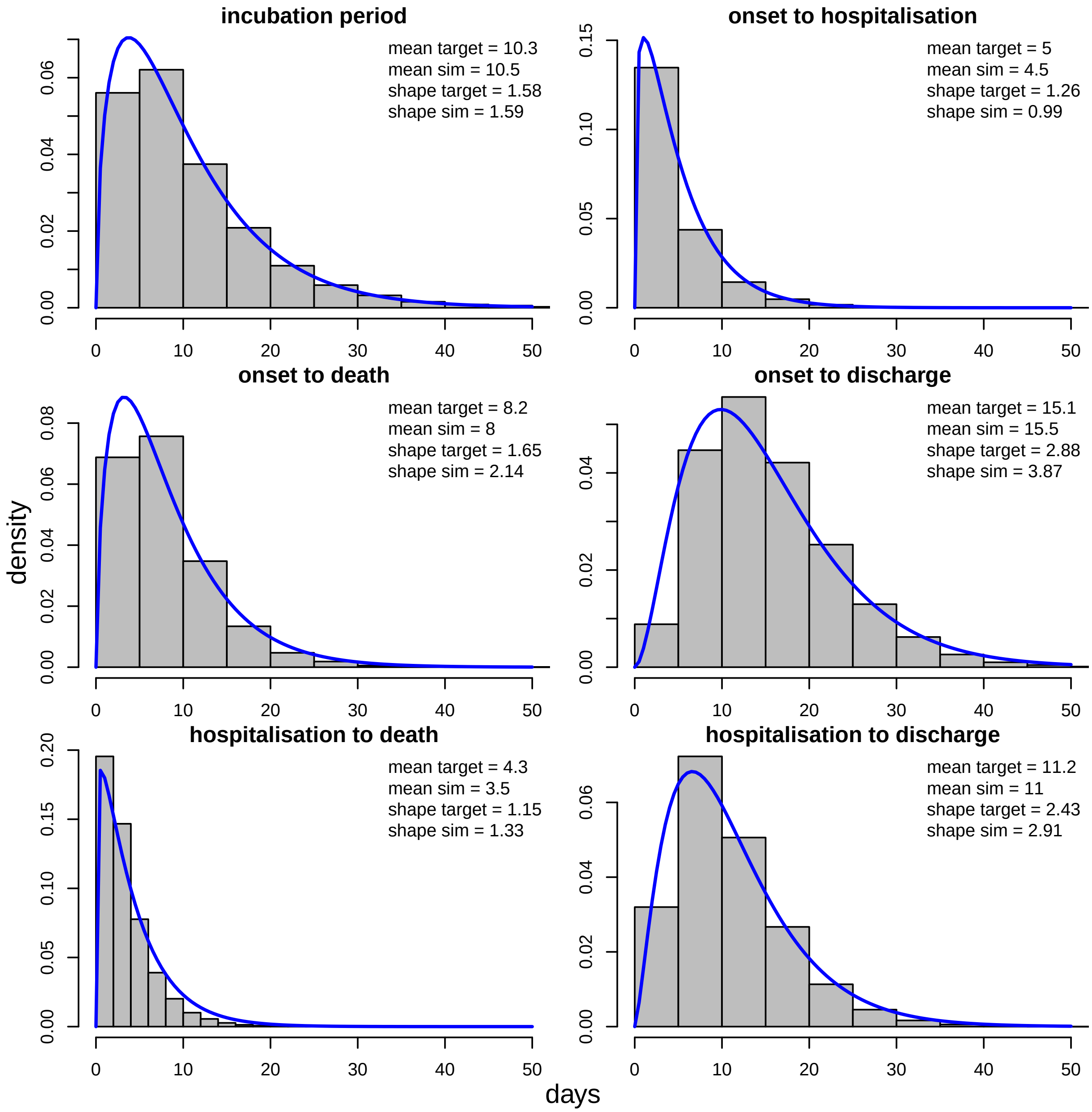

Figure SI.8: Comparison of the simulated delays distributions (histograms) and those fitted to the dataset (curves). The legends give the mean and shape parameters of gamma distributions fitted to the observed epidemic (mean and shape target) as well as to the model output (mean and shape sim) for each delay considered.

Table SI.8: Model parameters describing the test characteristics, their descriptions and assumed values for the scenario assuming perfect PCR performance and a more realistic lower test accuracy in the field.

Parameter	Description	Relation	Value.perfect	Value.real
sens_1	sensitivity of RDT		0.92	0.82
sens_2	sensitivity of PCR test		1.00	0.85
spec_1	specificity of RDT		0.85	0.80
spec_2	specificity of PCR test		1.00	0.95
τ_1	waiting time for RDT results in days		0.042	0.042
τ_2	waiting time for PCR test results in days		2	2
τ_3	waiting time for hospital discharge test in days	$2\tau_2$	4	4
τ_{symp}	duration of non-ebola symptoms (eg malaria) in days		3	3
τ_{sustest}	duration of stay in confirmed ward for non-ebola cases in days	$\tau_3 + \tau_{\text{symp}} - \tau_2$	5	5
η_1	rate of leaving the suspected ward per day	$1/\tau_1$	24.000	24.000
η_2	rate of leaving the high/low risk wards per day	$1/(\tau_2 - \tau_1)$	0.511	0.511
η_3	rate of leaving the confirmed ward for ebola cases per day	$1/\tau_3$	0.250	0.250
η_4	rate of leaving the confirmed ward for non-ebola cases per day	$1/\tau_{\text{sustest}}$	0.200	0.200

We used data on performance indicators of the response [30] to inform model parameters including the bed availability and the onset to hospitalisation delay throughout the epidemic, see Tables SI.9 and SI.10 for the time-dependent values assumed and their implementation dates. Note that the bed availability was based on published figures of available Ebola Treatment Unit (ETU) beds with an overall total of 60 beds added to account for pre-existing general health-care unit beds across the country.

Table SI.9: Number of health-care unit beds available throughout the epidemic.

Date	Beds
initial	60
01/06/2014	71
01/07/2014	121
06/09/2014	190
19/09/2014	225
22/09/2014	383
16/10/2014	406
20/11/2014	416
01/12/2014	577
11/12/2014	613
17/12/2014	675
22/12/2014	893
29/12/2014	956
06/01/2015	1106
13/01/2015	1267

Table SI.10: Mean onset to hospitalisation delay (in days) throughout the epidemic.

Date	Mean delay
initial	4.5
01/08/2014	3.8
01/09/2014	3.3
01/10/2014	3.4
01/11/2014	3.0
01/12/2014	2.6
01/01/2015	2.0
01/02/2015	2.0
01/03/2015	1.4
01/04/2015	1.2

The remaining model parameters were calibrated to achieve a good match of the model output to the reported weekly incidence of patients with confirmed and probable Ebola in Sierra Leone [31] between t_{start} on the 02 June 2014 and t_{end} on the 21 June 2015, see Table SI.11. We restricted the calibration period to this time frame as the deterministic model is unable to capture the stochastic fluctuations in the very early phase of the epidemic when case numbers were low. We started simulations at t_0 on the 28 April 2014 with $I_1(t_0)$ initial infectious cases, and $E_1(t_0) = R_0 I_1(t_0)$ initial exposed cases to have a more realistic distribution of cases between the different infected compartments resulting in a faster move towards a more stable distribution of cases among the infected compartments. We then calibrated the model parameters such that the model matched the observed incidence when there were sufficiently many cases for the deterministic assumptions to hold.

We assumed that the probability of a patient with Ebola seeking care increased throughout the epidemic, and allowed this value to change at 3 dates: the beginning of October, November and January. Similarly, we assumed that the death associated excess transmissibility described by the parameter β_D decreased throughout the epidemic through an increasing proportion of safe and dignified burials, and consequently allowed this parameter to decrease at the same time points.

Table SI.11: Epidemiological model parameters that were calibrated to match the observed incidence, their descriptions and baseline values under the scenarios of perfect and lower field based performance of PCR and RDT tests.

Parameter	Description	Relation	Implementation date	Value.perfect	Value.real
R_0	basic reproduction number		initial	1.7	1.8
I_0	initial number infectious		initial	35	35
β	transmission rate per day	$R_0/d_{I_{\text{tot}}}$	initial	0.11	0.11
κ	rate of non-ebola hospitalisations per day		initial	2.1e-09	1.9e-09
p_{HU}	probability of seeking care		initial	0.37	0.44
p_{HU}			01/10/2014	0.41	0.51
p_{HU}			01/11/2014	0.62	0.71
p_{HU}			01/01/2015	0.73	0.96
$\beta_{D,\text{com}}$			01/10/2014	15	15
$\beta_{D,\text{com}}$			01/11/2014	1.2	1.2
$\beta_{D,\text{com}}$			01/01/2015	1.1	1

3.3 Model output

We evaluated the impact of the various testing strategies by comparing various performance indicators of the counter-factual scenarios against those of the PCR-only testing strategy matched to the observed incidence. We focussed on the total epidemic size, here defined as the number of individuals infected between the 02 June 2014 and the 21 June 2015, the start and end dates used for the model calibration. This measure is highly correlated with the incidence at the peak as well as the incidence at the end date. We furthermore considered the total number of patients discharged from health-care units despite being actively infected with Ebola virus, due to either imperfect test accuracy or infection within the health-care unit setting after the sample for testing was drawn. Lastly we investigated the number of patients infected with Ebola turned away from health-care units due to bed capacity constraints. The magnitude of the latter two measures varied between the testing strategies and usefully illuminated the mechanisms giving rise to the final size observed under the different testing strategies.

3.3.1 Assuming perfect PCR and lab-determined RDT performance

The weekly incidence of patients with Ebola turned away from a health-care unit due to bed capacity constraints shows two peaks, the first and smaller around the beginning of September when rapidly increasing case numbers overwhelmed the small number of health-care unit beds available at the time, and a larger peak through November during the peak of the epidemic when the scale-up of bed capacity was not yet sufficient to keep pace with the increasing incidence (Figure SI.9, top). Bed capacity constraints have the largest impact in the PCR-only strategy as case numbers under this strategy are highest, reaching up to 250 cases per week through November. For both the RDT-only and dual testing strategy the peak in the number of patients turned away is lower and narrower, and for the fast PCR strategy only a small number of patients are turned away from the health-care unit during a single week, while near-perfect RDT performance at 99% sensitivity and specificity would have prevented any patients turned away.

The number of patients infected with Ebola discharged back to the community, either due to imperfect test accuracy or due to transmission within the health-care unit while awaiting test results is much more similar across the different testing strategies with a broad peak roughly following the peak in incidence (Figure SI.9, middle). At the assumed level of test accuracy for the currently available RDT, the fast PCR strategy does best in this metric as the shorter time spent within the holding area reduces within health-care unit transmission more than the segregation into low- and high-risk wards used in the dual testing strategy, particularly in the later stages of the epidemic when the proportion of patients with Ebola infection among those hospitalised is lower. However, a near-perfect RDT would be better still as the small number of patients misdiagnosed would be outweighed by the prevention of transmission while awaiting test results.

The proportion of patients with Ebola infection among those hospitalised decreases consistently throughout the epidemic with a steep drop from the peak onwards as incidence of patients with Ebola infection decreases, but awareness of the outbreak is still high leading to a constantly high rate of non-Ebola admissions (Figure SI.9, bottom). The patterns seen are similar across the different testing strategies with the exception of the near-perfect RDT-only strategy which shows considerably more efficient bed use with higher proportion of patients with Ebola infection in a health-care unit throughout the epidemic. For this scenario, 99% of patients witout Ebola have a negligible duration of stay in a health-care unit while awaiting the rapid test results, and the high specificity of the test means that only a very small number of these will be admitted to the confirmed ward, keeping the proportion of patients with Ebola among those hospitalised higher than under other RDT scenarios.

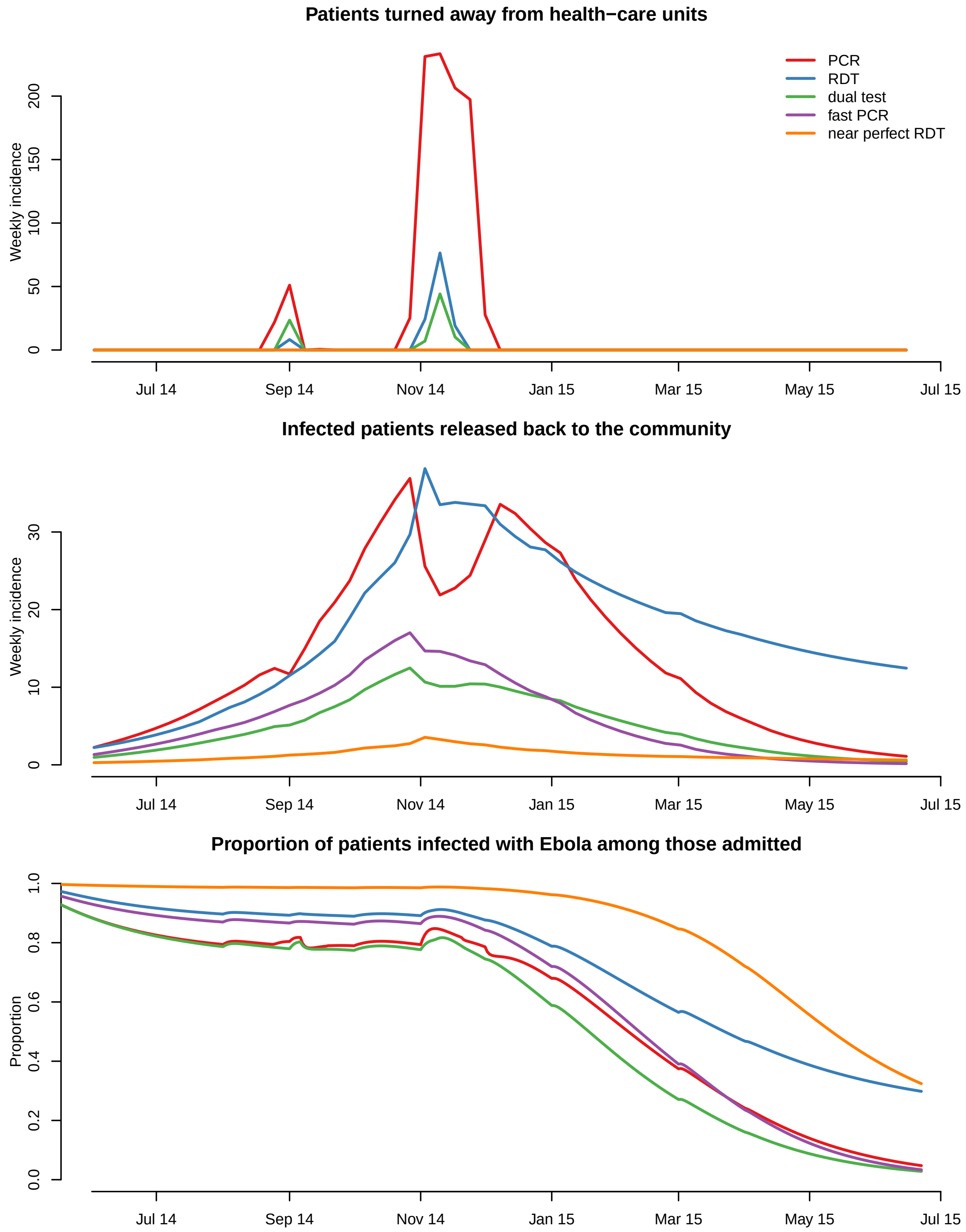

Figure SI.9: Weekly incidence of patients turned away from health-care units (top), patients infected with Ebola discharged back to the community (middle) and the proportion of patients infected with Ebola among those seeking care for suspected Ebola (bottom), assuming perfect PCR performance for the different testing strategies.

3.3.2 Assuming lower PCR and RDT performance in the field

The parameters used to calibrate the model with lower test sensitivity and specificity to the observed incidence are broadly similar to those assuming lab-based values for test sensitivity and specificity, although the rate of patients without Ebola seeking care is around 10% lower under the field bases scenario, while the probability a person infected with Ebola would seek care is higher by around 15 to 30% throughout the epidemic. The higher probability of people infected with Ebola seeking care and therefore being effectively isolated is counter-balanced by the fact that more patients with Ebola are discharged back to the community due to the lower test sensitivity of the PCR.

The incidence and bed use under the scenario assuming lower sensitivity and specificity of both PCR and RDT in the field than established in laboratory tests are very similar to those assuming perfect test sensitivity and specificity (Figure SI.10), as are the proportion of patients with Ebola among those hospitalised and the number of patients with Ebola turned away from hospitalisation due to bed capacity constraints (Figure SI.11, top and bottom), although even a 1-day turnaround for PCR results would not have been sufficient to completely avoid bed capacity constraints. Furthermore, the number of patients infected with Ebola discharged back to the community is around twice as high under the more pessimistic assumptions regarding test performance with less divergence between the different testing strategies as the difference in test performance between PCR and RDT is lower (Figure SI.11, middle).

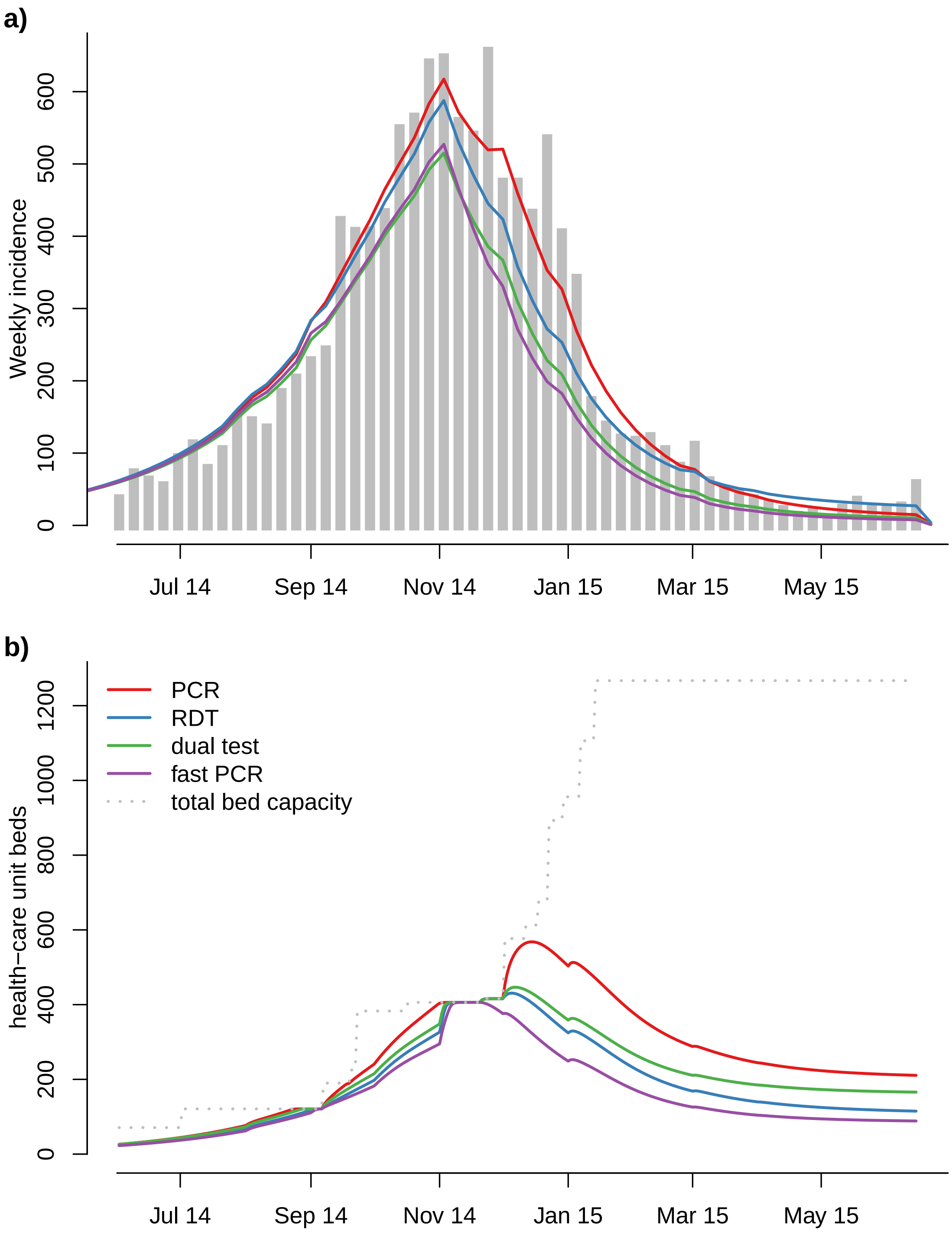

Figure SI.10: (a) Observed (grey bars) and expected (coloured lines) weekly incidence of confirmed and probable (CP) Ebola during the outbreak in Sierra Leone. The red line presents the expected incidence using the PCR-only strategy on which the model was calibrated. Other lines present the estimated incidence under distinct counter-factual scenarios: RDT-only (blue), dual testing strategy (green), PCR-only with faster test results delivered within 1 day (purple). (b) Bed capacity and usage throughout the epidemic in Sierra Leone. We assumed perfect accuracy for the PCR, while RDT accuracy was set to the officially reported sensitivity of 92% and specificity of 85%.

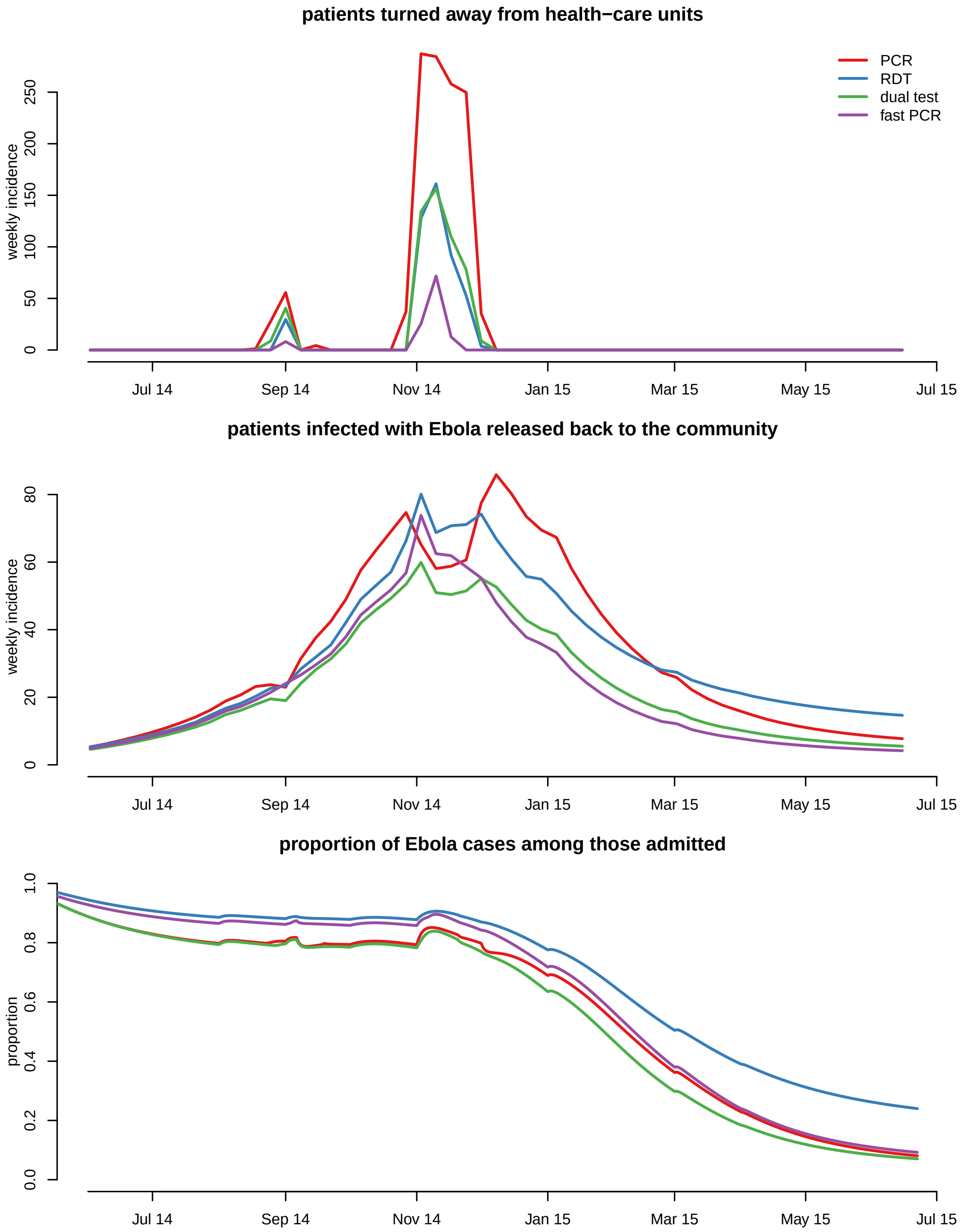

Figure SI.11: Weekly incidence of patients turned away from health-care units (top), patients infected with Ebola discharged back to the community (middle) and the proportion of patients with Ebola among those seeking care for suspected Ebola (bottom), assuming lower test accuracy in the field for the different testing strategies.

3.4 Sensitivity Analyses

3.4.1 Uncertainty in RDT characteristics

There is considerable uncertainty in the sensitivity and specificity of the currently licensed RDT, ReEBOV. Different values have been found in different studies [4, 5, 3] (Figure SI.12). While the confidence bounds of these estimate largely overlap, we evaluated the RDT-only and dual testing strategies using the alternative point estimates of sensitivity and specificity and found major impact on the effectiveness of the RDT-only or dual testing strategies (Figure SI.13).

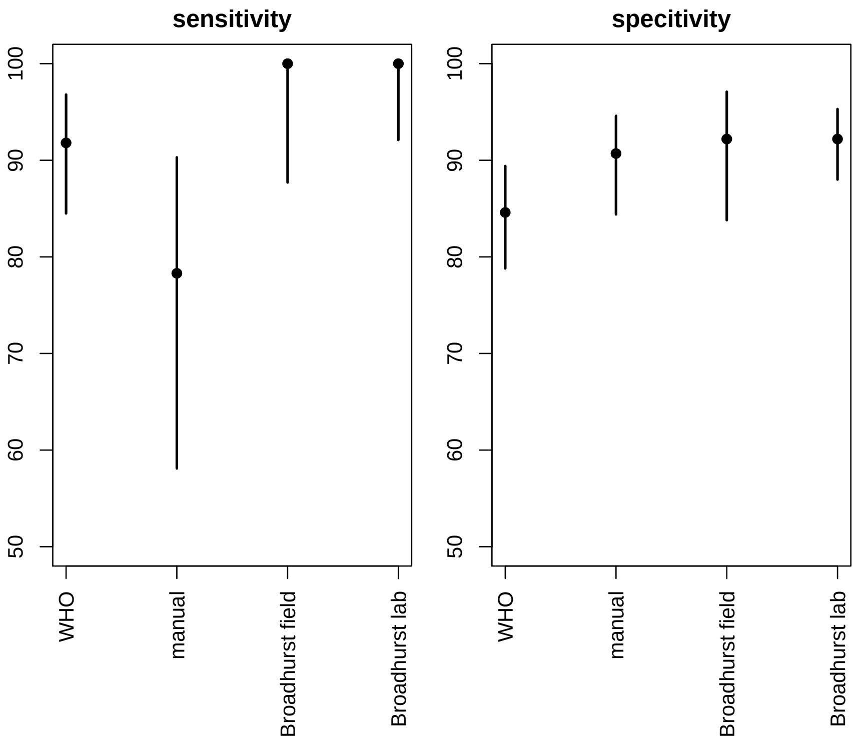

Figure SI.12: RDT sensitivity and specificity with 95% confidence bounds reported .

The highest sensitivity reported was 100% [5], and despite the slightly lower specificity of 92.2% this test resulted in a very similar epidemic size to the assumption of a near perfect RDT with 99% sensitivity and specificity, with a minimal improvement of the outcome if an RDT of these characteristics was used in a dual testing strategy.

The baseline test sensitivity and specificity of 91.8% and 84.6%, respectively as reported in the WHO report [3] result in an epidemic size very similar to the PCR-only scenario when using RDT-only, but a considerably reduced epidemic when incorporated into a dual testing strategy. However, with the low sensitivity reported in the official manual [4], the RDT-only strategy would have resulted in a considerably larger outbreak than what was observed. Still, in a dual strategy, even such a poor RDT would help to reduce the epidemic size to a similar level as with the WHO estimates of sensitivity and specificity we have used here as baseline values.

While the impact of the RDT-only strategy is highly dependent on the test characteristics, the impact of the dual strategy is much less dependent on the actual RDT characteristics, and the dual strategy is an improvement over the observed outbreak for all RDT characteristics considered here.

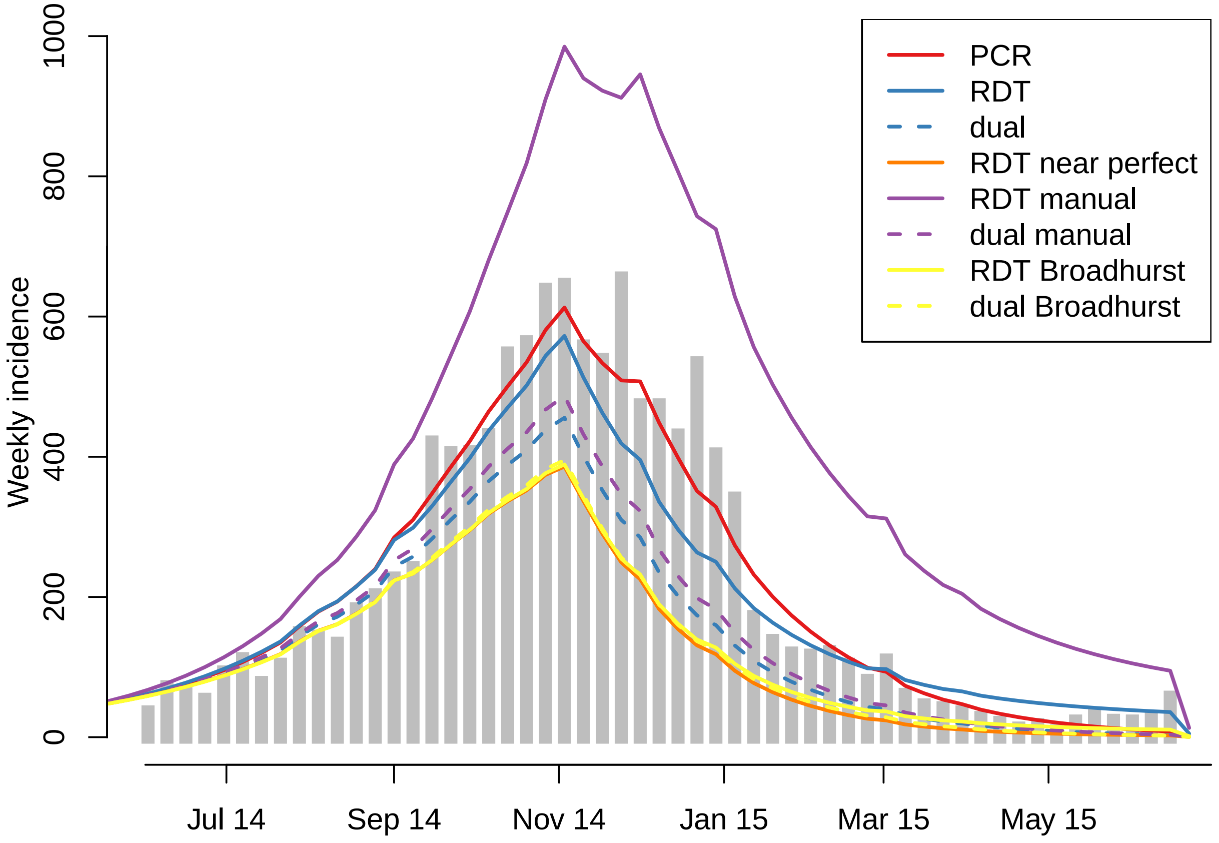

Figure SI.13: Observed (grey bars) and expected (coloured lines) weekly incidence of confirmed and probable (CP) Ebola during the outbreak in Sierra Leone. The red line presents the expected incidence using the PCR-only strategy on which the model was calibrated. Other solid lines present the estimated incidence under the RDT-only strategy and dashed lines the estimated incidence under the dual strategy assuming different test characteristics reported in the literature.

3.4.2 Sensitivity to the duration of symptoms in patients uninfected with Ebola

We have calibrated many of the model parameters to the observed outbreak dynamics, in particular those describing the duration of the epidemic stages for patients infected with Ebola . However, there is little data to inform the duration of symptoms for patients without Ebola who could be admitted to hospital due to diseases such as severe Lassa fever or malaria. We have assumed a baseline duration from hospitalisation to the clearance of symptoms for patients not infected with Ebola of $\tau_{\text{symp}} = 3$ days, which will result in an average hospital stay of 7 days under the baseline scenarios as upon clearance of symptoms two consecutive negative PCR tests are required prior to hospital discharge, a value similar to the average length of hospital stay for patients with Ebola, taking into account the different duration of hospitalisation for survivors and fatalities. Assuming that patients without Ebola would seek health-care after a similar delay following the onset of symptoms as patients with Ebola, this makes for a total duration of symptoms of 7.5 days which may be fairly long for patients with Malaria in treatment, but short for patients with severe Lassa fever, so appears a sensible compromise. However, owing to the lack of data to inform this parameter, here we investigate the effect that different assumptions for this parameter τ_{symp} have on the effectiveness of the different testing strategies.

Under perfect PCR assumptions, in the PCR-only and dual strategies no patients without Ebola infection are admitted to the confirmed ward, and therefore the duration of their symptoms does not change the dynamics. However, for the RDT-only strategy a longer duration of symptoms for patients without Ebola results in increased bed demand as well as increased opportunities for nosocomial transmission, making the RDT-only strategy less effective particularly in the tail of the epidemic. The magnitude of this effect can be seen in Figure SI.14. For the range of values investigated here the RDT-only strategy leads to a change in final size between 89% and 102% compared to the PCR-only strategy.

3.4.3 Sensitivity to parameter values

To assess the impact of the calibrated parameter values on the relative performance of the different testing strategies we performed one-way sensitivity analyses. First, we calculated the final size in the baseline strategy (PCR-only) using the calibrated parameter values. For each parameter, we separately established the parameter value for which the final size in this strategy was 5% more or less than the mean value keeping all other parameters at their calibrated values. We then calculated the final size for each strategy under each of these parameter sets and evaluated the % reduction in final size under each counterfactual scenario relative to the baseline PCR-only scenario.

The impact of each parameter in each testing strategy is shown in Figures SI.15 and SI.16, assuming perfect or realistic PCR sensitivity and specificity and the corresponding values of sensitivity and specificity of the RDT, respectively, while Figures SI.17 and SI.18 show the impact of varying the different model parameters over time.

The impact of parameters governing the whole epidemic period (R_0, I_0 and κ) roughly mirrors the shape of the epidemic curve (Figures SI.17 and SI.18). Parameters only valid for a limited period throughout the epidemic include $p_{\text{HU,i}}$ and $\beta_{\text{D.com,i}}$, $i = 0, 1, 2, 3$, applying to the periods up to 30th September 2014, 1st to 31st Oct 2014, 1st Nov to 31st Dec 2014 and from 1st Jan 2015 onwards, respectively. Note that we have fixed the value of $\beta_{\text{D.com,0}} = 16$ per day throughout, and varying parameters $\beta_{\text{D.com,2}}$ and $\beta_{\text{D.com,3}}$ between the most extreme values of 1 and 16 per day gave a difference in final size of less than 5% at the lower bound, so we did not include these parameters in the sensitivity analyses here. The other parameters only valid for a limited period have zero impact before coming into effect, but have a lasting though decreasing effect on the tail of the epidemic even when they are no longer active.

The parameter to which the relative incidence under the different testing strategies is most sensitive is κ which governs the flow of patients without Ebola into a health-care unit. Varying κ to achieve a 5% difference in cumulative incidence under the PCR only testing strategy has a much lower impact on the other strategies. For strategies employing an RDT (either alone or as part of the dual strategy), most of the patients without Ebola are sent home upon receipt of the initial test result or are segregated from the majority of the patients with Ebola while waiting for the PCR results, such that they are less likely to get infected as a result of their health-care unit visit and therefore the overall number of patients without Ebola seeking care is less important under these scenarios. The mechanism of reducing the impact of κ on the PCR-only strategies with different assumptions is different: when PCR results are obtained faster, bed limitation is much less of

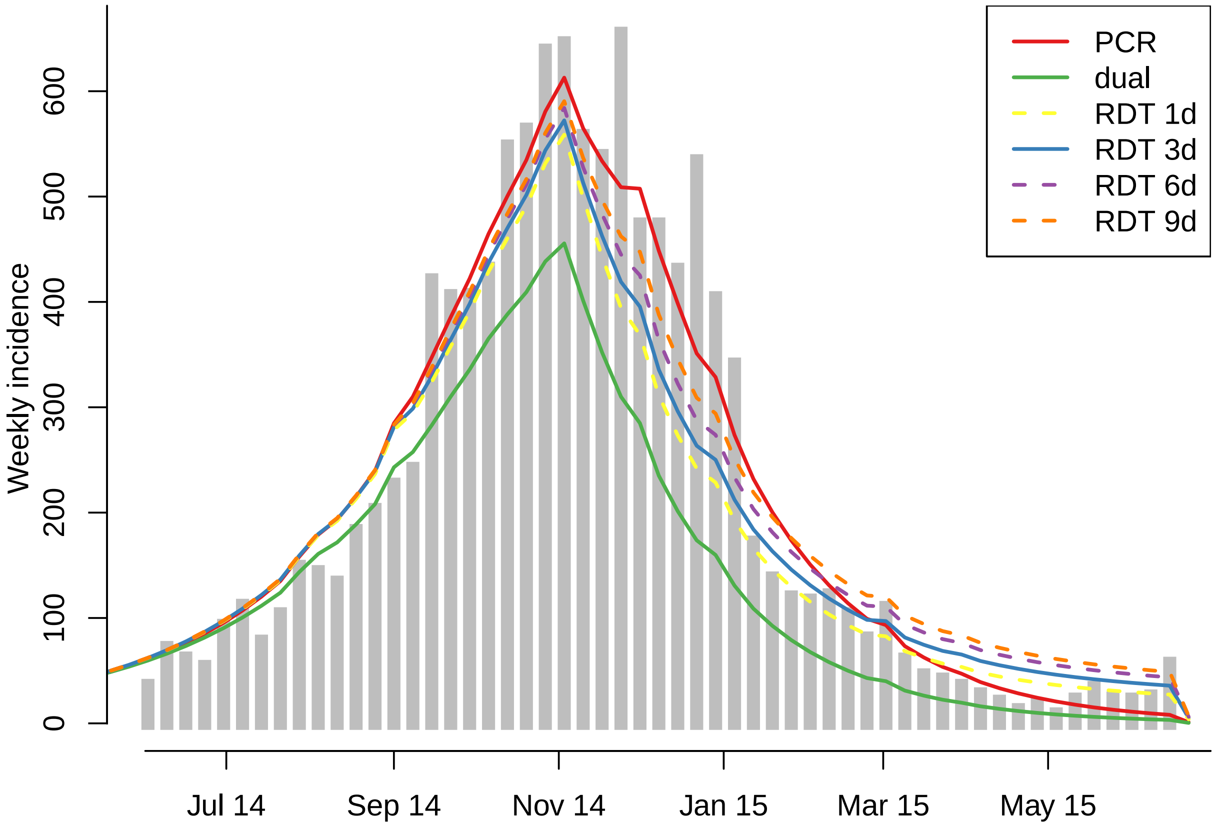

Figure SI.14: Observed (grey bars) and expected (coloured lines) weekly incidence of confirmed and probable (CP) Ebola during the outbreak in Sierra Leone. The red line presents the expected incidence using the PCR-only strategy on which the model was calibrated. Other solid lines present the estimated incidence under the dual RDT-only strategy with baseline assumptions of the duration of symptoms from hospitalisation for patients without Ebola, $\tau_{\text{symp}} = 3$ and dashed lines the estimated incidence under the RDT-only strategy assuming alternative values for τ_{symp}. Solid lines for the baseline scenarios, dashed lines for the sensitivity analyses for the RDT-only scenario.

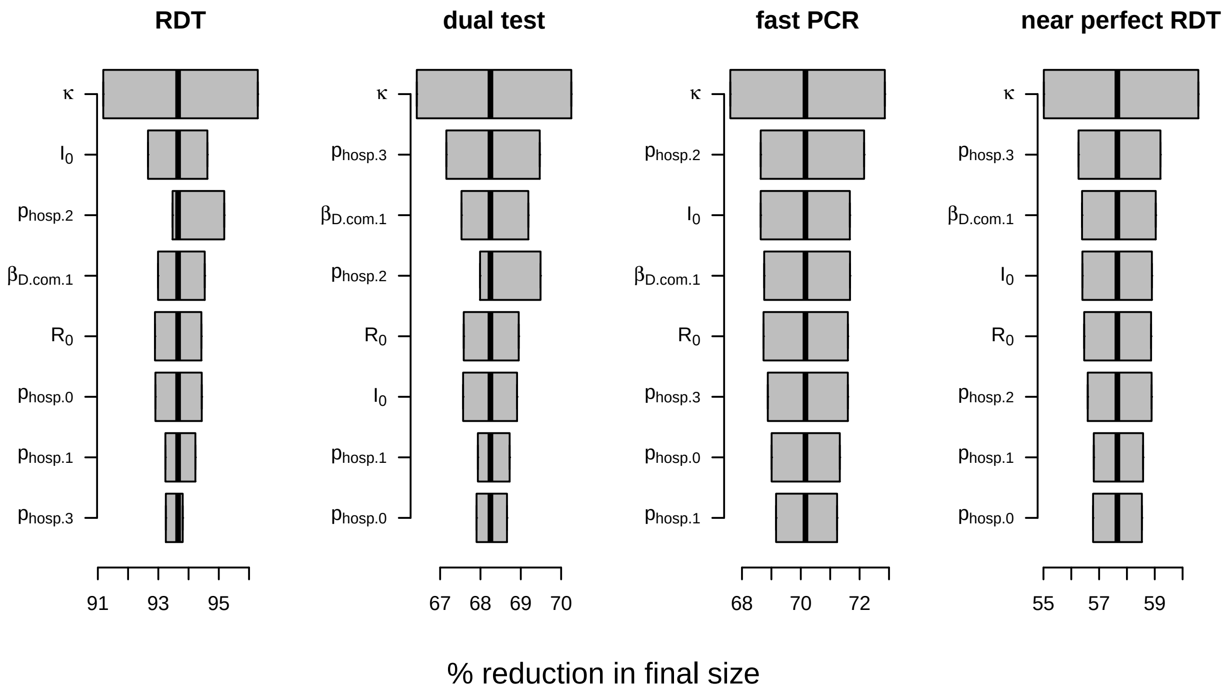

Figure SI.15: Tornado plot assessing sensitivity of the final epidemic size under the various counterfactual testing strategies relative to the baseline PCR-only strategy to the individual calibrated model parameters, assuming perfect PCR performance.

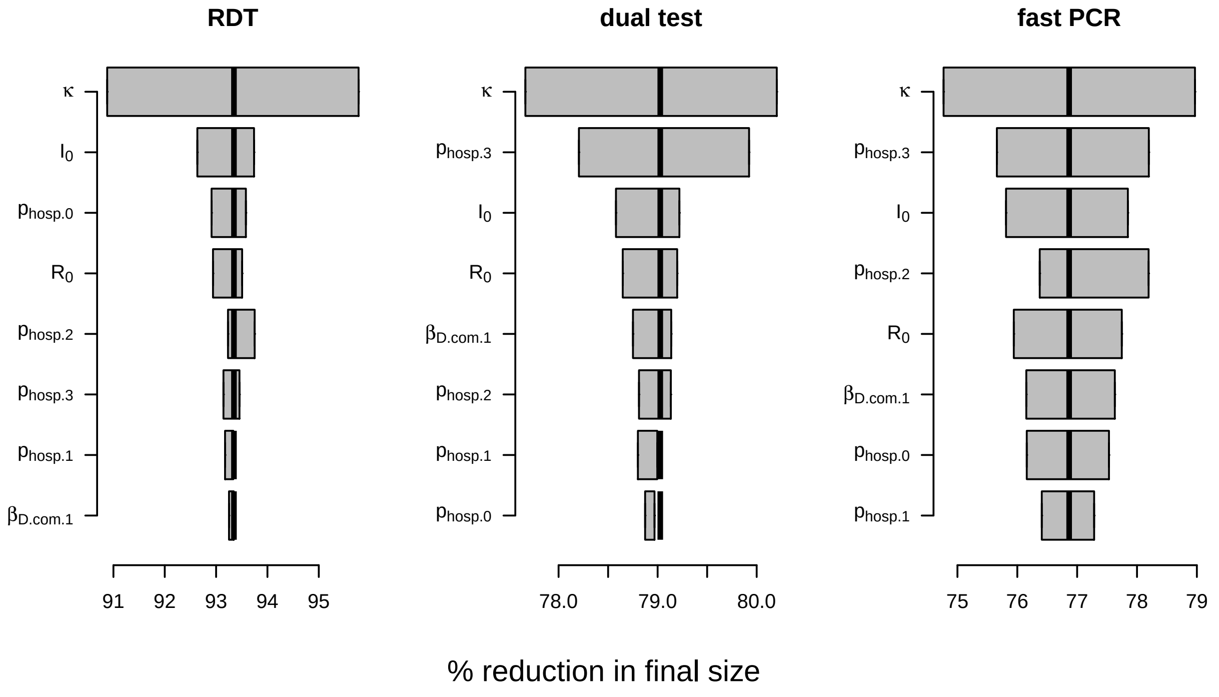

Figure SI.16: Tornado plot assessing sensitivity of the final epidemic size under the various counterfactual testing strategies relative to the baseline PCR-only strategy to the individual calibrated model parameters, assuming realistic PCR performance.

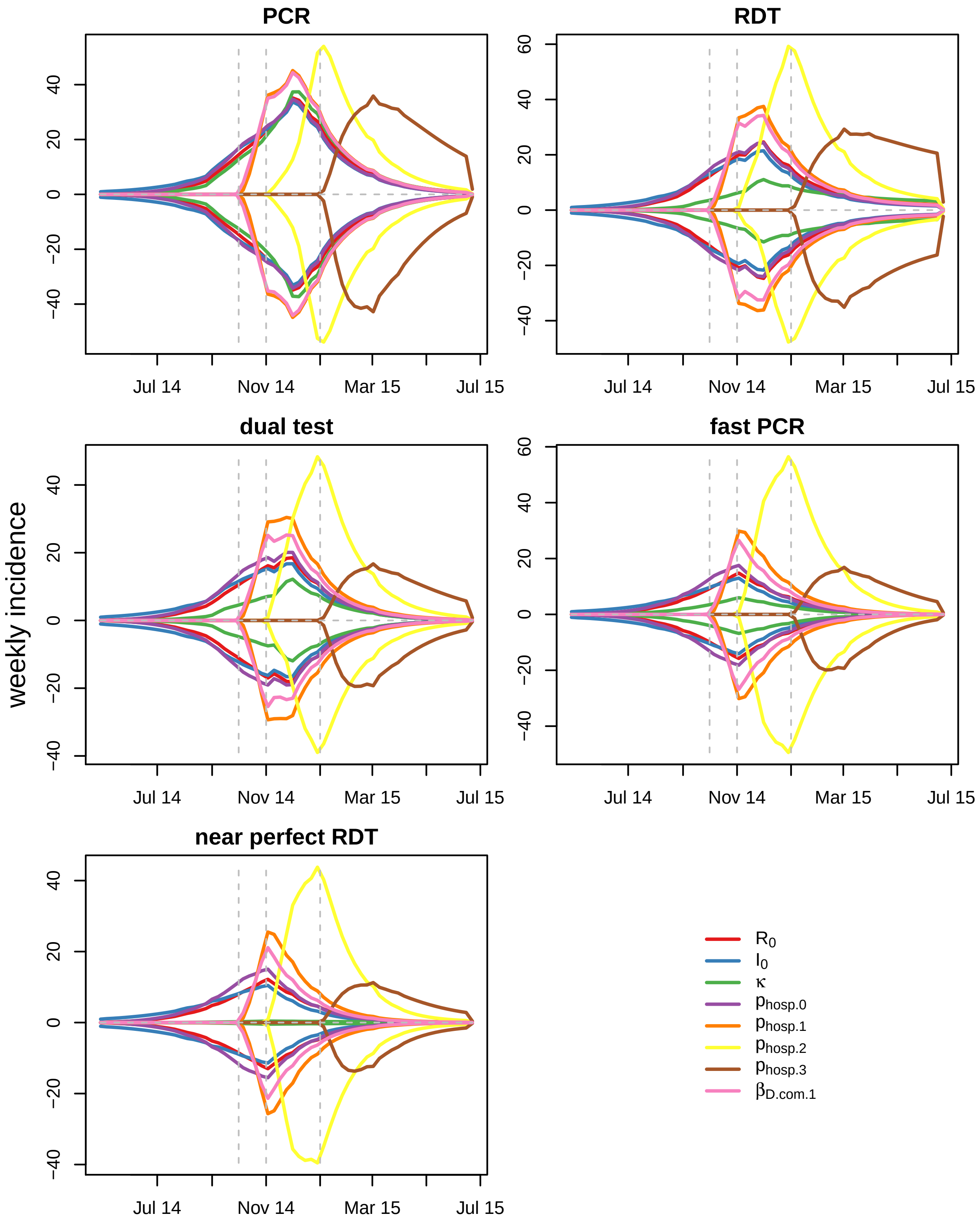

Figure SI.17: Difference in weekly incidence between the models evaluated for the parameters giving 5% larger or lower final size and the baseline parameter values for the different testing strategies assuming perfect PCR performance. Vertical dashed lines show the dates at which the probability of hospitalisation p_{HU} and the death-associated transmissibility in the community are allowed to vary, separating the initial period 0 from periods 1, 2, and 3.

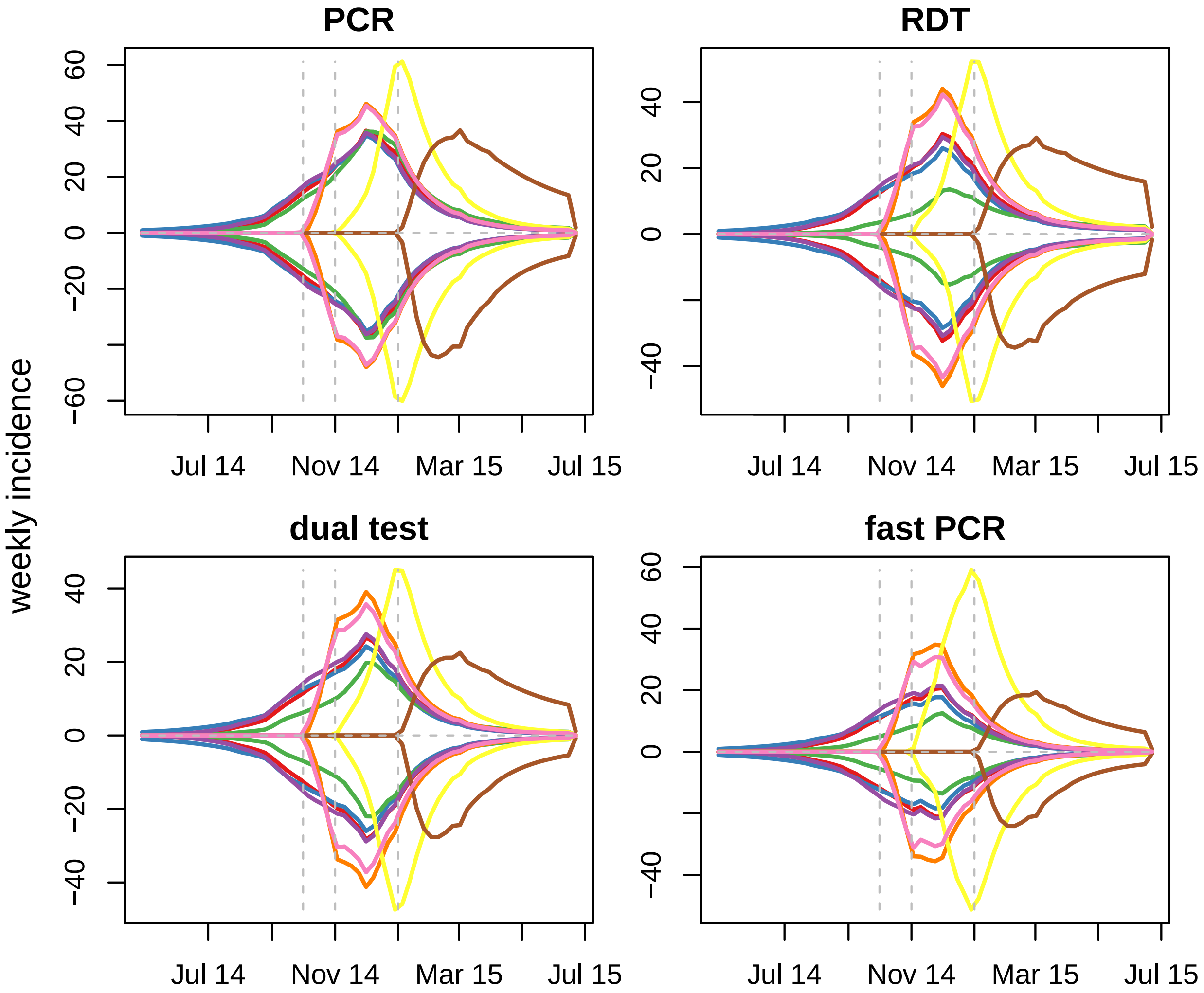

Figure SI.18: Difference in weekly incidence between the models evaluated for the parameters giving 5% larger or lower final size and the baseline parameter values for the different testing strategies assuming realistic PCR performance. Vertical dashed lines show the dates at which the probability of hospitalisation p_{HU} and the death-associated transmissibility in the community are allowed to vary, separating the initial period 0 from periods 1, 2, and 3. Line colours as in Figure SI.16

an issue and the patients without Ebola do not block up health-care unit beds that would urgently be needed for patients with Ebola. Therefore the number of patients without Ebola seeking care has less impact on the overall epidemic size in these scenarios than in the baseline PCR scenario.

References

[1] World Health Organization. *WHO Emergency Quality Assessment Mechanism for EVD IVDs. PUBLIC REPORT. Product: RealStar Filovirus Screen RT-PCR Kit 1.0*. Report. 2015. URL: http://www.who.int/diagnostics_laboratory/procurement/141125_evd_public_report_altona_v1.pdf.

[2] Altona Diagnostics. *RealStar Filovirus Screen RT-PCR Kit 1.0*. Report. 2014. URL: http://www.altona-diagnostics.com/tl_files/website/downloads/RealStar_Filovirus%20Screen%20RT-PCR%20Kit%2010_CE_WEB_2014-08-29.pdf.

[3] World Health Organization. *WHO Emergency Use Assessment and Listing for Ebola Virus Disease IVDs. PUBLIC REPORT. Product: ReEBOV Antigen Rapid Test Kit*. Report. 2015. URL: http://www.who.int/diagnostics_laboratory/procurement/150219_reebov_antigen_rapid_test_public_report.pdf?ua=1.

[4] Corgenix Inc. *ReEBOV Antigen Rapid Test (Ebolavirus VP40 Antigen Detection) Instructions for Use*. Report. 2015. URL: ftp://ftp.corgenix.com/Intra/ReEBOV/14005.pdf.

[5] Mara Jana Broadhurst et al. "ReEBOV Antigen Rapid Test kit for point-of-care and laboratory-based testing for Ebola virus disease: a field validation study". In: *The Lancet* (2015).

[6] World Health Organization. *WHO Emergency Use Assessment and Listing for EVD IVDs PUBLIC REPORT Product: Xpert Ebola Assay*. Report. 2015. URL: http://www.who.int/diagnostics_laboratory/procurement/150508_pr_expert_ebola_test_usa.pdf.

[7] Cepheid. *Cepheid Xpert Ebola Assay*. Report. 2015. URL: http://www.fda.gov/downloads/MedicalDevices/Safety/EmergencySituations/UCM439578.pdf.

[8] World Health Organization. *WHO Emergency Use Assessment and Listing Procedure for EVD IVDs. PUBLIC REPORT. Product: Liferiver - Ebola Virus (EBOV) Real Time RT-PCR Kit*. Report. 2015. URL: http://www.who.int/diagnostics_laboratory/procurement/150427_liferiver_china_public_report.pdf.

[9] Obelis S.A. & Shanghai ZJ Bio-Tech Co. Ltd. *LifeRiver Ebola Virus (EBOV) Real Time RT-PCR Kit User Manual*. Report. 2012. URL: http://www.biosb.com/wp-content/uploads/QR-0220-01-Ebola-Virus-EBOV-Real-Time-RT-PCR-Kit.pdf.

[10] Food and Drug Administration. *2014 Ebola Virus Emergency Use Authorizations*. Web Page. 2014-2015. URL: http://www.fda.gov/medicaldevices/safety/emergencysituations/ucm161496.htm#ebola.

[11] Naval Medical Research Center for The U.S. Department of Defense. *Ebola Zaire (EZ1) rRT-PCR (TaqMan) Assay Instruction Booklet*. Report. 2014.

[12] Centers for Disease Control and Prevention. *Ebola Virus NP Real-Time RT-PCR Assay Instructions for Use*. Report. 2014.

[13] Centers for Disease Control and Prevention. *Ebola Virus VP40 Real-Time RT-PCR Assay Instructions for Use*. Report. 2014.

[14] BioFire Defense LLC. *FilmArray BioThreat-E Instructions for Use*. Report. 2014.

[15] BioFire Defense LLC. *FilmArray NGDS BT-E Assay Instructions for Use*. Report. 2015.

[16] TIB MOLBIOL. *LightMix Ebola Zaire rRT-PCR Test Instructions for Use*. Report. 2014.

[17] OraSure Technologies Inc. *OraQuick Ebola Rapid Antigen Test*. Tech. rep. 2015.

[18] NF Walker et al. "Evaluation of a point-of-care blood test for identification of Ebola virus disease at Ebola holding units, Western Area, Sierra Leone, January to February 2015". In: *Euro Surveill* 20.12 (2015).

[19] Chun-Wan Yen et al. "Multicolored silver nanoparticles for multiplexed disease diagnostics: distinguishing dengue, yellow fever, and Ebola viruses". In: *Lab on a chip* 15.7 (2015), pp. 1638–1641.

[20] Anne Trafton. *Quick test for Ebola: Simple paper strip can diagnose Ebola and other fevers within 10 minutes*. Electronic Article. 2015. URL: http://newsoffice.mit.edu/2015/ten-minute-ebola-test-0224.

[21] Emily Wasserman. *French scientists roll out rapid diagnostic test for Ebola*. Electronic Article. 2014. URL: http://www.fiercediagnostics.com/story/french-scientists-roll-out-rapid-diagnostic-test-ebola/2014-10-23.

[22] CEA. *Technical validation of a diagnostic test Ebola in less than fifteen minutes in the field (in French)*. Web Page. 2014. URL: http://dsv.cea.fr/dsv/Pages/Actualites/presse/communiques-presse/2014/Virus-Ebola--validation-technique-d%E2%80%99un-test-de-diagnostic-en-moins-de-quinze-minutes-sur-le-terrain.aspx.

[23] J.-C. Manuguerra. "Molecular pathogen detection in the field, directly at the point of care". In: *Conference: Targeting Ebola at Institute Pasteur in Paris May 2015* (2015).

[24] Vanhomwegen, J. et al. "Molecular pathogen detection in the field, directly at the point of care". In: *in preparation* ().

[25] EM Leroy et al. "Diagnosis of Ebola haemorrhagic fever by RT-PCR in an epidemic setting". In: *Journal of medical virology* 60 (2000), pp. 463–7. ISSN: 0146-6615.

[26] Christian L Althaus. "Estimating the reproduction number of Ebola virus (EBOV) during the 2014 outbreak in West Africa". In: *PLoS Currents* 6 (2014).

[27] Chowell, Gerardo and Hengartner, Nick W and Castillo-Chavez, Carlos and Fenimore, Paul W and Hyman, JM. "The basic reproductive number of Ebola and the effects of public health measures: the cases of Congo and Uganda". In: *Journal of Theoretical Biology* 229.1 (2004), pp. 119–126.

[28] WHO Ebola Response Team. "Ebola virus disease in West Africa the first 9 months of the epidemic and forward projections". In: *N Engl J Med* 371.16 (2014), pp. 1481–95. DOI: 10.1056/NEJMoa1411100.

[29] WHO Ebola Response Team. "West African Ebola epidemic after one year slowing but not yet under control". In: *N Engl J Med.* 372.6 (2015), pp. 584–587. DOI: 10.1056/NEJMc1414992.

[30] World Health Organization. *Ebola Situation Reports - archive*. Online, accessed 6 June 2015. 2015. URL: \url{http://apps.who.int/ebola/en/ebola-situation-reports-archive}.

[31] World Health Organization. *Ebola data and statistics*. Online, accessed 6 June 2015. 2015. URL: \url{http://apps.who.int/gho/data/node.ebola-sitrep}.
